# Supplementary material for: Quantitative proteomic analysis of extracellular vesicles in response to baculovirus infection of a Trichoplusia ni cell line
Source: PLoS One. 2023 Jan 30;18(1):e0281060. doi: 10.1371/journal.pone.0281060 (PMC9886248; doi:10.1371/journal.pone.0281060)
Supplement: S1 Raw images — (PDF) [file pone.0281060.s001.pdf]

1. Western blot Hsp90

Marker* uninfected infected

130 kDa
95 kDa
72 kDa

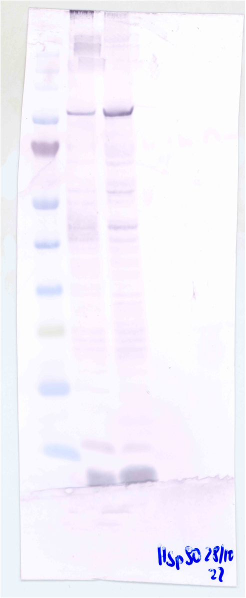

2. Western blot Hsp70

Marker* uninfected infected

130 kDa
95 kDa
72 kDa

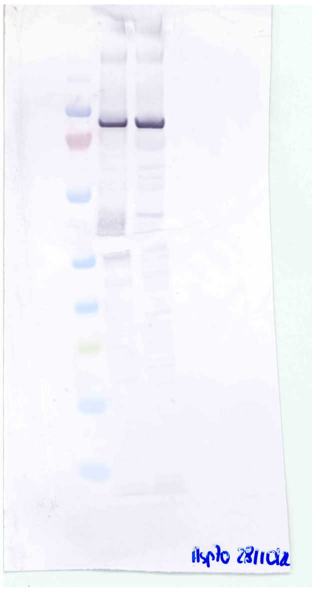

* Color prestained protein standard broad range (New England Biolabs)

Note that, compared to the applied protein standard, bands appeared slightly higher than the expected sizes. According to the manufacturer, apparent molecular weight values of the protein standard depend on gel types and percentages. The dye attached to the proteins, might carry a charge resulting in a charge alteration of the proteins, ultimately altering the mobility of the proteins, what can lead to slight differences in observed migration patterns.
