# Supplementary material for: Quantitative proteomic analysis of extracellular vesicles in response to baculovirus infection of a Trichoplusia ni cell line
Source: PLoS One. 2023 Jan 30;18(1):e0281060. doi: 10.1371/journal.pone.0281060 (PMC9886248; doi:10.1371/journal.pone.0281060)
Supplement: S3 File — (DOCX) [file pone.0281060.s004.docx]

**KEGG Mapper Search Result**

 [**tnl01100**](https://www.genome.jp/kegg-bin/show_pathway?166859291342340/tnl01100.args) **Metabolic pathways - Trichoplusia ni (cabbage looper) (**[**238**](javascript:display('tnl01100'))**)**

[tnl:113491632](https://www.genome.jp/entry/tnl:113491632) [K01514](https://www.genome.jp/entry/K01514) exopolyphosphatase PRUNE1

[tnl:113491873](https://www.genome.jp/entry/tnl:113491873) [K00600](https://www.genome.jp/entry/K00600) serine hydroxymethyltransferase, cytosolic isoform X1

[tnl:113492142](https://www.genome.jp/entry/tnl:113492142) [K03331](https://www.genome.jp/entry/K03331) L-xylulose reductase-like

[tnl:113492358](https://www.genome.jp/entry/tnl:113492358) [K00658](https://www.genome.jp/entry/K00658) dihydrolipoyllysine-residue succinyltransferase component of 2-oxoglutarate dehydrogenase complex, mitochondrial-like

[tnl:113492488](https://www.genome.jp/entry/tnl:113492488) [K01800](https://www.genome.jp/entry/K01800) probable maleylacetoacetate isomerase 2 isoform X1

[tnl:113492544](https://www.genome.jp/entry/tnl:113492544) [K00940](https://www.genome.jp/entry/K00940) nucleoside diphosphate kinase

[tnl:113492551](https://www.genome.jp/entry/tnl:113492551) [K01432](https://www.genome.jp/entry/K01432) kynurenine formamidase isoform X1

[tnl:113492555](https://www.genome.jp/entry/tnl:113492555) [K21032](https://www.genome.jp/entry/K21032) beta-1,4-glucuronyltransferase 1

[tnl:113492561](https://www.genome.jp/entry/tnl:113492561) [K18081](https://www.genome.jp/entry/K18081) myotubularin-related protein 2

[tnl:113492628](https://www.genome.jp/entry/tnl:113492628) [K08738](https://www.genome.jp/entry/K08738) cytochrome c

[tnl:113492630](https://www.genome.jp/entry/tnl:113492630) [K00029](https://www.genome.jp/entry/K00029) LOW QUALITY PROTEIN: NADP-dependent malic enzyme-like

[tnl:113492641](https://www.genome.jp/entry/tnl:113492641) [K01070](https://www.genome.jp/entry/K01070) S-formylglutathione hydrolase isoform X1

[tnl:113492664](https://www.genome.jp/entry/tnl:113492664) [K08660](https://www.genome.jp/entry/K08660) cytosolic non-specific dipeptidase

[tnl:113492686](https://www.genome.jp/entry/tnl:113492686) [K15730](https://www.genome.jp/entry/K15730) uncharacterized protein CG16817-like

[tnl:113492710](https://www.genome.jp/entry/tnl:113492710) [K00031](https://www.genome.jp/entry/K00031) isocitrate dehydrogenase [NADP] cytoplasmic-like

[tnl:113492726](https://www.genome.jp/entry/tnl:113492726) [K11204](https://www.genome.jp/entry/K11204) glutamate--cysteine ligase catalytic subunit

[tnl:113492751](https://www.genome.jp/entry/tnl:113492751) [K02144](https://www.genome.jp/entry/K02144) V-type proton ATPase subunit H isoform X1

[tnl:113492846](https://www.genome.jp/entry/tnl:113492846) [K14410](https://www.genome.jp/entry/K14410) prostatic acid phosphatase-like isoform X1

[tnl:113492848](https://www.genome.jp/entry/tnl:113492848) [K11153](https://www.genome.jp/entry/K11153) retinol dehydrogenase 13-like

[tnl:113492937](https://www.genome.jp/entry/tnl:113492937) [K14454](https://www.genome.jp/entry/K14454) aspartate aminotransferase, cytoplasmic

[tnl:113493022](https://www.genome.jp/entry/tnl:113493022) [K00657](https://www.genome.jp/entry/K00657) diamine acetyltransferase 2-like

[tnl:113493061](https://www.genome.jp/entry/tnl:113493061) [K01897](https://www.genome.jp/entry/K01897) long-chain-fatty-acid--CoA ligase 4 isoform X1

[tnl:113493155](https://www.genome.jp/entry/tnl:113493155) [K13755](https://www.genome.jp/entry/K13755) calcium/calmodulin-dependent 3',5'-cyclic nucleotide phosphodiesterase 1 isoform X1

[tnl:113493425](https://www.genome.jp/entry/tnl:113493425) [K01904](https://www.genome.jp/entry/K01904) luciferin 4-monooxygenase-like

[tnl:113493465](https://www.genome.jp/entry/tnl:113493465) [K00079](https://www.genome.jp/entry/K00079) carbonyl reductase [NADPH] 1-like

[tnl:113493473](https://www.genome.jp/entry/tnl:113493473) [K02146](https://www.genome.jp/entry/K02146) V-type proton ATPase subunit d

[tnl:113493478](https://www.genome.jp/entry/tnl:113493478) [K01251](https://www.genome.jp/entry/K01251) adenosylhomocysteinase

[tnl:113493488](https://www.genome.jp/entry/tnl:113493488) [K07515](https://www.genome.jp/entry/K07515) trifunctional enzyme subunit alpha, mitochondrial

[tnl:113493566](https://www.genome.jp/entry/tnl:113493566) [K08678](https://www.genome.jp/entry/K08678) UDP-glucuronic acid decarboxylase 1

[tnl:113493809](https://www.genome.jp/entry/tnl:113493809) [K13566](https://www.genome.jp/entry/K13566) omega-amidase NIT2-like isoform X1

[tnl:113493828](https://www.genome.jp/entry/tnl:113493828) [K00948](https://www.genome.jp/entry/K00948) ribose-phosphate pyrophosphokinase 2

[tnl:113493839](https://www.genome.jp/entry/tnl:113493839) [K00920](https://www.genome.jp/entry/K00920) phosphatidylinositol 5-phosphate 4-kinase type-2 alpha isoform X1

[tnl:113493924](https://www.genome.jp/entry/tnl:113493924) [K01803](https://www.genome.jp/entry/K01803) triosephosphate isomerase

[tnl:113493984](https://www.genome.jp/entry/tnl:113493984) [K00710](https://www.genome.jp/entry/K00710) polypeptide N-acetylgalactosaminyltransferase 35A-like isoform X1

[tnl:113494140](https://www.genome.jp/entry/tnl:113494140) [K01469](https://www.genome.jp/entry/K01469) 5-oxoprolinase

[tnl:113494165](https://www.genome.jp/entry/tnl:113494165) [K00966](https://www.genome.jp/entry/K00966) mannose-1-phosphate guanyltransferase alpha-A

[tnl:113494198](https://www.genome.jp/entry/tnl:113494198) [K11205](https://www.genome.jp/entry/K11205) glutamate--cysteine ligase regulatory subunit isoform X1

[tnl:113494305](https://www.genome.jp/entry/tnl:113494305) [K21456](https://www.genome.jp/entry/K21456) glutathione synthetase-like isoform X1

[tnl:113494314](https://www.genome.jp/entry/tnl:113494314) [K00364](https://www.genome.jp/entry/K00364) GMP reductase 1-like

[tnl:113494316](https://www.genome.jp/entry/tnl:113494316) [K01810](https://www.genome.jp/entry/K01810) glucose-6-phosphate isomerase

[tnl:113494320](https://www.genome.jp/entry/tnl:113494320) [K10532](https://www.genome.jp/entry/K10532) heparan-alpha-glucosaminide N-acetyltransferase-like

[tnl:113494349](https://www.genome.jp/entry/tnl:113494349) [K07509](https://www.genome.jp/entry/K07509) LOW QUALITY PROTEIN: trifunctional enzyme subunit beta, mitochondrial-like

[tnl:113494362](https://www.genome.jp/entry/tnl:113494362) [K07253](https://www.genome.jp/entry/K07253) macrophage migration inhibitory factor-like isoform X1

[tnl:113494468](https://www.genome.jp/entry/tnl:113494468) [K00079](https://www.genome.jp/entry/K00079) carbonyl reductase [NADPH] 3-like

[tnl:113494495](https://www.genome.jp/entry/tnl:113494495) [K00927](https://www.genome.jp/entry/K00927) phosphoglycerate kinase

[tnl:113494548](https://www.genome.jp/entry/tnl:113494548) [K00816](https://www.genome.jp/entry/K00816) kynurenine--oxoglutarate transaminase 3 isoform X1

[tnl:113494579](https://www.genome.jp/entry/tnl:113494579) [K00764](https://www.genome.jp/entry/K00764) amidophosphoribosyltransferase-like isoform X1

[tnl:113494584](https://www.genome.jp/entry/tnl:113494584) [K01587](https://www.genome.jp/entry/K01587) multifunctional protein ADE2

[tnl:113494590](https://www.genome.jp/entry/tnl:113494590) [K00876](https://www.genome.jp/entry/K00876) probable uridine-cytidine kinase isoform X1

[tnl:113494620](https://www.genome.jp/entry/tnl:113494620) [K01698](https://www.genome.jp/entry/K01698) delta-aminolevulinic acid dehydratase

[tnl:113494681](https://www.genome.jp/entry/tnl:113494681) [K02147](https://www.genome.jp/entry/K02147) V-type proton ATPase subunit B

[tnl:113494704](https://www.genome.jp/entry/tnl:113494704) [K01069](https://www.genome.jp/entry/K01069) hydroxyacylglutathione hydrolase, mitochondrial isoform X1

[tnl:113494755](https://www.genome.jp/entry/tnl:113494755) [K12304](https://www.genome.jp/entry/K12304) soluble calcium-activated nucleotidase 1

[tnl:113494797](https://www.genome.jp/entry/tnl:113494797) [K01756](https://www.genome.jp/entry/K01756) adenylosuccinate lyase-like

[tnl:113494878](https://www.genome.jp/entry/tnl:113494878) [K21798](https://www.genome.jp/entry/K21798) phosphatidylinositide phosphatase SAC2-like isoform X1

[tnl:113494907](https://www.genome.jp/entry/tnl:113494907) [K00799](https://www.genome.jp/entry/K00799) glutathione S-transferase 1-like

[tnl:113495149](https://www.genome.jp/entry/tnl:113495149) [K06210](https://www.genome.jp/entry/K06210) nicotinamide/nicotinic acid mononucleotide adenylyltransferase 1

[tnl:113495260](https://www.genome.jp/entry/tnl:113495260) [K01953](https://www.genome.jp/entry/K01953) asparagine synthetase [glutamine-hydrolyzing]

[tnl:113495376](https://www.genome.jp/entry/tnl:113495376) [K01196](https://www.genome.jp/entry/K01196) glycogen debranching enzyme

[tnl:113495499](https://www.genome.jp/entry/tnl:113495499) [K01836](https://www.genome.jp/entry/K01836) phosphoacetylglucosamine mutase

[tnl:113495603](https://www.genome.jp/entry/tnl:113495603) [K01679](https://www.genome.jp/entry/K01679) fumarate hydratase, mitochondrial-like isoform X1

[tnl:113495619](https://www.genome.jp/entry/tnl:113495619) [K11188](https://www.genome.jp/entry/K11188) peroxiredoxin-6

[tnl:113495745](https://www.genome.jp/entry/tnl:113495745) [K13647](https://www.genome.jp/entry/K13647) procollagen-lysine,2-oxoglutarate 5-dioxygenase isoform X1

[tnl:113495760](https://www.genome.jp/entry/tnl:113495760) [K03781](https://www.genome.jp/entry/K03781) catalase

[tnl:113496055](https://www.genome.jp/entry/tnl:113496055) [K03783](https://www.genome.jp/entry/K03783) purine nucleoside phosphorylase-like isoform X1

[tnl:113496116](https://www.genome.jp/entry/tnl:113496116) [K02145](https://www.genome.jp/entry/K02145) V-type proton ATPase catalytic subunit A

[tnl:113496121](https://www.genome.jp/entry/tnl:113496121) [K01915](https://www.genome.jp/entry/K01915) glutamine synthetase 2 cytoplasmic-like isoform X1

[tnl:113496175](https://www.genome.jp/entry/tnl:113496175) [K14410](https://www.genome.jp/entry/K14410) prostatic acid phosphatase isoform X1

[tnl:113496177](https://www.genome.jp/entry/tnl:113496177) [K00058](https://www.genome.jp/entry/K00058) D-3-phosphoglycerate dehydrogenase

[tnl:113496232](https://www.genome.jp/entry/tnl:113496232) [K02149](https://www.genome.jp/entry/K02149) V-type proton ATPase subunit D

[tnl:113496310](https://www.genome.jp/entry/tnl:113496310) [K00850](https://www.genome.jp/entry/K00850) ATP-dependent 6-phosphofructokinase isoform X1

[tnl:113496346](https://www.genome.jp/entry/tnl:113496346) [K12667](https://www.genome.jp/entry/K12667) dolichyl-diphosphooligosaccharide--protein glycosyltransferase subunit 2

[tnl:113496347](https://www.genome.jp/entry/tnl:113496347) [K10808](https://www.genome.jp/entry/K10808) ribonucleoside-diphosphate reductase subunit M2

[tnl:113496536](https://www.genome.jp/entry/tnl:113496536) [K15730](https://www.genome.jp/entry/K15730) uncharacterized protein CG16817-like

[tnl:113496554](https://www.genome.jp/entry/tnl:113496554) [K02377](https://www.genome.jp/entry/K02377) GDP-L-fucose synthase isoform X1

[tnl:113496570](https://www.genome.jp/entry/tnl:113496570) [K00942](https://www.genome.jp/entry/K00942) guanylate kinase isoform X1

[tnl:113496731](https://www.genome.jp/entry/tnl:113496731) [K00799](https://www.genome.jp/entry/K00799) uncharacterized protein LOC113496731

[tnl:113496805](https://www.genome.jp/entry/tnl:113496805) [K00286](https://www.genome.jp/entry/K00286) pyrroline-5-carboxylate reductase-like

[tnl:113496813](https://www.genome.jp/entry/tnl:113496813) [K00939](https://www.genome.jp/entry/K00939) adenylate kinase

[tnl:113496832](https://www.genome.jp/entry/tnl:113496832) [K00261](https://www.genome.jp/entry/K00261) glutamate dehydrogenase, mitochondrial

[tnl:113496966](https://www.genome.jp/entry/tnl:113496966) [K17285](https://www.genome.jp/entry/K17285) methanethiol oxidase

[tnl:113496976](https://www.genome.jp/entry/tnl:113496976) [K00750](https://www.genome.jp/entry/K00750) uncharacterized protein LOC113496976 isoform X1

[tnl:113497008](https://www.genome.jp/entry/tnl:113497008) [K00799](https://www.genome.jp/entry/K00799) glutathione S-transferase 1-1-like

[tnl:113497009](https://www.genome.jp/entry/tnl:113497009) [K00799](https://www.genome.jp/entry/K00799) glutathione S-transferase 1-1-like isoform X1

[tnl:113497121](https://www.genome.jp/entry/tnl:113497121) [K10807](https://www.genome.jp/entry/K10807) ribonucleoside-diphosphate reductase large subunit

[tnl:113497125](https://www.genome.jp/entry/tnl:113497125) [K05858](https://www.genome.jp/entry/K05858) 1-phosphatidylinositol 4,5-bisphosphate phosphodiesterase classes I and II

[tnl:113497126](https://www.genome.jp/entry/tnl:113497126) [K00654](https://www.genome.jp/entry/K00654) serine palmitoyltransferase 2

[tnl:113497192](https://www.genome.jp/entry/tnl:113497192) [K00049](https://www.genome.jp/entry/K00049) glyoxylate reductase/hydroxypyruvate reductase-like

[tnl:113497263](https://www.genome.jp/entry/tnl:113497263) [K04097](https://www.genome.jp/entry/K04097) glutathione S-transferase 2-like

[tnl:113497272](https://www.genome.jp/entry/tnl:113497272) [K20279](https://www.genome.jp/entry/K20279) synaptojanin-1

[tnl:113497274](https://www.genome.jp/entry/tnl:113497274) [K00710](https://www.genome.jp/entry/K00710) N-acetylgalactosaminyltransferase 7

[tnl:113497289](https://www.genome.jp/entry/tnl:113497289) [K12373](https://www.genome.jp/entry/K12373) chitooligosaccharidolytic beta-N-acetylglucosaminidase

[tnl:113497302](https://www.genome.jp/entry/tnl:113497302) [K00072](https://www.genome.jp/entry/K00072) sepiapterin reductase

[tnl:113497383](https://www.genome.jp/entry/tnl:113497383) [K00472](https://www.genome.jp/entry/K00472) prolyl 4-hydroxylase subunit alpha-1-like

[tnl:113497424](https://www.genome.jp/entry/tnl:113497424) [K00797](https://www.genome.jp/entry/K00797) spermidine synthase

[tnl:113497645](https://www.genome.jp/entry/tnl:113497645) [K00507](https://www.genome.jp/entry/K00507) acyl-CoA Delta(11) desaturase-like

[tnl:113497652](https://www.genome.jp/entry/tnl:113497652) [K00688](https://www.genome.jp/entry/K00688) glycogen phosphorylase

[tnl:113497664](https://www.genome.jp/entry/tnl:113497664) [K01110](https://www.genome.jp/entry/K01110) phosphatidylinositol 3,4,5-trisphosphate 3-phosphatase and dual-specificity protein phosphatase PTEN

[tnl:113497732](https://www.genome.jp/entry/tnl:113497732) [K00106](https://www.genome.jp/entry/K00106) xanthine dehydrogenase

[tnl:113497787](https://www.genome.jp/entry/tnl:113497787) [K17108](https://www.genome.jp/entry/K17108) non-lysosomal glucosylceramidase

[tnl:113497832](https://www.genome.jp/entry/tnl:113497832) [K00025](https://www.genome.jp/entry/K00025) malate dehydrogenase, cytoplasmic

[tnl:113497839](https://www.genome.jp/entry/tnl:113497839) [K12316](https://www.genome.jp/entry/K12316) lysosomal alpha-glucosidase-like

[tnl:113497863](https://www.genome.jp/entry/tnl:113497863) [K00121](https://www.genome.jp/entry/K00121) alcohol dehydrogenase class-3

[tnl:113497936](https://www.genome.jp/entry/tnl:113497936) [K00079](https://www.genome.jp/entry/K00079) carbonyl reductase [NADPH] 1-like

[tnl:113497947](https://www.genome.jp/entry/tnl:113497947) [K00844](https://www.genome.jp/entry/K00844) hexokinase type 2 isoform X1

[tnl:113497953](https://www.genome.jp/entry/tnl:113497953) [K01081](https://www.genome.jp/entry/K01081) cytosolic purine 5'-nucleotidase isoform X1

[tnl:113498020](https://www.genome.jp/entry/tnl:113498020) [K00626](https://www.genome.jp/entry/K00626) acetyl-CoA acetyltransferase, mitochondrial

[tnl:113498047](https://www.genome.jp/entry/tnl:113498047) [K15759](https://www.genome.jp/entry/K15759) putative inositol monophosphatase 3

[tnl:113498344](https://www.genome.jp/entry/tnl:113498344) [K00665](https://www.genome.jp/entry/K00665) fatty acid synthase isoform X1

[tnl:113498369](https://www.genome.jp/entry/tnl:113498369) [K00814](https://www.genome.jp/entry/K00814) alanine aminotransferase 1-like isoform X1

[tnl:113498448](https://www.genome.jp/entry/tnl:113498448) [K14163](https://www.genome.jp/entry/K14163) bifunctional glutamate/proline--tRNA ligase isoform X1

[tnl:113498462](https://www.genome.jp/entry/tnl:113498462) [K00275](https://www.genome.jp/entry/K00275) pyridoxine-5'-phosphate oxidase-like

[tnl:113498502](https://www.genome.jp/entry/tnl:113498502) [K16794](https://www.genome.jp/entry/K16794) LOW QUALITY PROTEIN: lissencephaly-1 homolog

[tnl:113498503](https://www.genome.jp/entry/tnl:113498503) [K16794](https://www.genome.jp/entry/K16794) LOW QUALITY PROTEIN: lissencephaly-1 homolog

[tnl:113498563](https://www.genome.jp/entry/tnl:113498563) [K01512](https://www.genome.jp/entry/K01512) acylphosphatase-1-like

[tnl:113498597](https://www.genome.jp/entry/tnl:113498597) [K00693](https://www.genome.jp/entry/K00693) glycogen [starch] synthase

[tnl:113498601](https://www.genome.jp/entry/tnl:113498601) [K12373](https://www.genome.jp/entry/K12373) beta-hexosaminidase subunit beta-like isoform X1

[tnl:113498801](https://www.genome.jp/entry/tnl:113498801) [K13988](https://www.genome.jp/entry/K13988) ADP-ribose pyrophosphatase, mitochondrial

[tnl:113498903](https://www.genome.jp/entry/tnl:113498903) [K11262](https://www.genome.jp/entry/K11262) acetyl-CoA carboxylase isoform X1

[tnl:113498917](https://www.genome.jp/entry/tnl:113498917) [K00710](https://www.genome.jp/entry/K00710) putative polypeptide N-acetylgalactosaminyltransferase 9 isoform X1

[tnl:113498973](https://www.genome.jp/entry/tnl:113498973) [K01106](https://www.genome.jp/entry/K01106) uncharacterized protein LOC113498973 isoform X1

[tnl:113499004](https://www.genome.jp/entry/tnl:113499004) [K01689](https://www.genome.jp/entry/K01689) enolase

[tnl:113499034](https://www.genome.jp/entry/tnl:113499034) [K00868](https://www.genome.jp/entry/K00868) pyridoxal kinase

[tnl:113499077](https://www.genome.jp/entry/tnl:113499077) [K01231](https://www.genome.jp/entry/K01231) alpha-mannosidase 2 isoform X1

[tnl:113499175](https://www.genome.jp/entry/tnl:113499175) [K01487](https://www.genome.jp/entry/K01487) guanine deaminase-like

[tnl:113499192](https://www.genome.jp/entry/tnl:113499192) [K10524](https://www.genome.jp/entry/K10524) nicotinamide riboside kinase 2

[tnl:113499205](https://www.genome.jp/entry/tnl:113499205) [K01816](https://www.genome.jp/entry/K01816) putative hydroxypyruvate isomerase

[tnl:113499273](https://www.genome.jp/entry/tnl:113499273) [K00772](https://www.genome.jp/entry/K00772) S-methyl-5'-thioadenosine phosphorylase-like

[tnl:113499295](https://www.genome.jp/entry/tnl:113499295) [K13711](https://www.genome.jp/entry/K13711) phosphatidylinositol 4-kinase type 2-alpha-like isoform X1

[tnl:113499305](https://www.genome.jp/entry/tnl:113499305) [K01487](https://www.genome.jp/entry/K01487) LOW QUALITY PROTEIN: guanine deaminase-like

[tnl:113499314](https://www.genome.jp/entry/tnl:113499314) [K01555](https://www.genome.jp/entry/K01555) fumarylacetoacetase

[tnl:113499351](https://www.genome.jp/entry/tnl:113499351) [K01951](https://www.genome.jp/entry/K01951) GMP synthase [glutamine-hydrolyzing] isoform X1

[tnl:113499353](https://www.genome.jp/entry/tnl:113499353) [K00861](https://www.genome.jp/entry/K00861) riboflavin kinase

[tnl:113499397](https://www.genome.jp/entry/tnl:113499397) [K02150](https://www.genome.jp/entry/K02150) V-type proton ATPase subunit E

[tnl:113499532](https://www.genome.jp/entry/tnl:113499532) [K01597](https://www.genome.jp/entry/K01597) diphosphomevalonate decarboxylase-like

[tnl:113499552](https://www.genome.jp/entry/tnl:113499552) [K00799](https://www.genome.jp/entry/K00799) uncharacterized protein LOC113499552

[tnl:113499695](https://www.genome.jp/entry/tnl:113499695) [K01520](https://www.genome.jp/entry/K01520) deoxyuridine 5'-triphosphate nucleotidohydrolase

[tnl:113499721](https://www.genome.jp/entry/tnl:113499721) [K00615](https://www.genome.jp/entry/K00615) transketolase-like protein 2

[tnl:113500107](https://www.genome.jp/entry/tnl:113500107) [K00864](https://www.genome.jp/entry/K00864) glycerol kinase

[tnl:113500261](https://www.genome.jp/entry/tnl:113500261) [K01053](https://www.genome.jp/entry/K01053) regucalcin-like isoform X1

[tnl:113500327](https://www.genome.jp/entry/tnl:113500327) [K02564](https://www.genome.jp/entry/K02564) glucosamine-6-phosphate isomerase isoform X1

[tnl:113500423](https://www.genome.jp/entry/tnl:113500423) [K00873](https://www.genome.jp/entry/K00873) pyruvate kinase-like isoform X1

[tnl:113500508](https://www.genome.jp/entry/tnl:113500508) [K02151](https://www.genome.jp/entry/K02151) V-type proton ATPase subunit F

[tnl:113500574](https://www.genome.jp/entry/tnl:113500574) [K00831](https://www.genome.jp/entry/K00831) probable phosphoserine aminotransferase

[tnl:113500605](https://www.genome.jp/entry/tnl:113500605) [K02154](https://www.genome.jp/entry/K02154) V-type proton ATPase 116 kDa subunit a

[tnl:113500626](https://www.genome.jp/entry/tnl:113500626) [K08963](https://www.genome.jp/entry/K08963) methylthioribose-1-phosphate isomerase

[tnl:113500912](https://www.genome.jp/entry/tnl:113500912) [K00602](https://www.genome.jp/entry/K00602) bifunctional purine biosynthesis protein PURH-like

[tnl:113500921](https://www.genome.jp/entry/tnl:113500921) [K00128](https://www.genome.jp/entry/K00128) retinal dehydrogenase 1-like

[tnl:113500928](https://www.genome.jp/entry/tnl:113500928) [K00789](https://www.genome.jp/entry/K00789) S-adenosylmethionine synthase isoform X1

[tnl:113501060](https://www.genome.jp/entry/tnl:113501060) [K00036](https://www.genome.jp/entry/K00036) glucose-6-phosphate 1-dehydrogenase

[tnl:113501099](https://www.genome.jp/entry/tnl:113501099) [K00310](https://www.genome.jp/entry/K00310) pyrimidodiazepine synthase-like

[tnl:113501133](https://www.genome.jp/entry/tnl:113501133) [K00967](https://www.genome.jp/entry/K00967) ethanolamine-phosphate cytidylyltransferase isoform X1

[tnl:113501180](https://www.genome.jp/entry/tnl:113501180) [K01099](https://www.genome.jp/entry/K01099) type II inositol 1,4,5-trisphosphate 5-phosphatase

[tnl:113501183](https://www.genome.jp/entry/tnl:113501183) [K00547](https://www.genome.jp/entry/K00547) uncharacterized protein LOC113501183

[tnl:113501275](https://www.genome.jp/entry/tnl:113501275) [K19269](https://www.genome.jp/entry/K19269) glycerol-3-phosphate phosphatase-like

[tnl:113501491](https://www.genome.jp/entry/tnl:113501491) [K01519](https://www.genome.jp/entry/K01519) inosine triphosphate pyrophosphatase

[tnl:113501500](https://www.genome.jp/entry/tnl:113501500) [K08041](https://www.genome.jp/entry/K08041) Ca(2+)/calmodulin-responsive adenylate cyclase-like

[tnl:113501642](https://www.genome.jp/entry/tnl:113501642) [K07023](https://www.genome.jp/entry/K07023) HD domain-containing protein 2

[tnl:113501660](https://www.genome.jp/entry/tnl:113501660) [K00963](https://www.genome.jp/entry/K00963) UTP--glucose-1-phosphate uridylyltransferase isoform X1

[tnl:113501798](https://www.genome.jp/entry/tnl:113501798) [K18532](https://www.genome.jp/entry/K18532) adenylate kinase isoenzyme 6 homolog

[tnl:113501843](https://www.genome.jp/entry/tnl:113501843) [K00682](https://www.genome.jp/entry/K00682) gamma-glutamylcyclotransferase-like isoform X1

[tnl:113501879](https://www.genome.jp/entry/tnl:113501879) [K14394](https://www.genome.jp/entry/K14394) low molecular weight phosphotyrosine protein phosphatase 1-like

[tnl:113502164](https://www.genome.jp/entry/tnl:113502164) [K01758](https://www.genome.jp/entry/K01758) cystathionine gamma-lyase-like

[tnl:113502186](https://www.genome.jp/entry/tnl:113502186) [K00763](https://www.genome.jp/entry/K00763) nicotinate phosphoribosyltransferase isoform X1

[tnl:113502268](https://www.genome.jp/entry/tnl:113502268) [K00802](https://www.genome.jp/entry/K00802) spermine synthase

[tnl:113502333](https://www.genome.jp/entry/tnl:113502333) [K03661](https://www.genome.jp/entry/K03661) V-type proton ATPase 21 kDa proteolipid subunit

[tnl:113502400](https://www.genome.jp/entry/tnl:113502400) [K13811](https://www.genome.jp/entry/K13811) bifunctional 3'-phosphoadenosine 5'-phosphosulfate synthase isoform X1

[tnl:113502690](https://www.genome.jp/entry/tnl:113502690) [K01952](https://www.genome.jp/entry/K01952) phosphoribosylformylglycinamidine synthase-like

[tnl:113502955](https://www.genome.jp/entry/tnl:113502955) [K15376](https://www.genome.jp/entry/K15376) gephyrin

[tnl:113503107](https://www.genome.jp/entry/tnl:113503107) [K01057](https://www.genome.jp/entry/K01057) 6-phosphogluconolactonase

[tnl:113503154](https://www.genome.jp/entry/tnl:113503154) [K02148](https://www.genome.jp/entry/K02148) V-type proton ATPase subunit C

[tnl:113503426](https://www.genome.jp/entry/tnl:113503426) [K13403](https://www.genome.jp/entry/K13403) bifunctional methylenetetrahydrofolate dehydrogenase/cyclohydrolase, mitochondrial isoform X1

[tnl:113503573](https://www.genome.jp/entry/tnl:113503573) [K00128](https://www.genome.jp/entry/K00128) aldehyde dehydrogenase, mitochondrial

[tnl:113503650](https://www.genome.jp/entry/tnl:113503650) [K01077](https://www.genome.jp/entry/K01077) alkaline phosphatase-like

[tnl:113503659](https://www.genome.jp/entry/tnl:113503659) [K01835](https://www.genome.jp/entry/K01835) phosphoglucomutase

[tnl:113503681](https://www.genome.jp/entry/tnl:113503681) [K00856](https://www.genome.jp/entry/K00856) adenosine kinase isoform X1

[tnl:113503711](https://www.genome.jp/entry/tnl:113503711) [K01784](https://www.genome.jp/entry/K01784) UDP-glucose 4-epimerase-like

[tnl:113503730](https://www.genome.jp/entry/tnl:113503730) [K01711](https://www.genome.jp/entry/K01711) GDP-mannose 4,6 dehydratase

[tnl:113503987](https://www.genome.jp/entry/tnl:113503987) [K05858](https://www.genome.jp/entry/K05858) 1-phosphatidylinositol 4,5-bisphosphate phosphodiesterase isoform X1

[tnl:113504172](https://www.genome.jp/entry/tnl:113504172) [K00287](https://www.genome.jp/entry/K00287) dihydrofolate reductase isoform X1

[tnl:113504203](https://www.genome.jp/entry/tnl:113504203) [K01904](https://www.genome.jp/entry/K01904) 4-coumarate--CoA ligase 1-like

[tnl:113504269](https://www.genome.jp/entry/tnl:113504269) [K00889](https://www.genome.jp/entry/K00889) phosphatidylinositol 4-phosphate 5-kinase type-1 alpha-like isoform X1

[tnl:113504438](https://www.genome.jp/entry/tnl:113504438) [K00031](https://www.genome.jp/entry/K00031) isocitrate dehydrogenase [NADP] cytoplasmic isoform X1

[tnl:113504454](https://www.genome.jp/entry/tnl:113504454) [K12670](https://www.genome.jp/entry/K12670) dolichyl-diphosphooligosaccharide--protein glycosyltransferase 48 kDa subunit

[tnl:113504558](https://www.genome.jp/entry/tnl:113504558) [K11540](https://www.genome.jp/entry/K11540) CAD protein isoform X1

[tnl:113504592](https://www.genome.jp/entry/tnl:113504592) [K17725](https://www.genome.jp/entry/K17725) uncharacterized protein LOC113504592

[tnl:113504622](https://www.genome.jp/entry/tnl:113504622) [K01769](https://www.genome.jp/entry/K01769) receptor-type guanylate cyclase Gyc76C-like isoform X1

[tnl:113504708](https://www.genome.jp/entry/tnl:113504708) [K01082](https://www.genome.jp/entry/K01082) 3'(2'),5'-bisphosphate nucleotidase 1-like isoform X1

[tnl:113504794](https://www.genome.jp/entry/tnl:113504794) [K01490](https://www.genome.jp/entry/K01490) AMP deaminase 2-like isoform X1

[tnl:113504856](https://www.genome.jp/entry/tnl:113504856) [K01939](https://www.genome.jp/entry/K01939) adenylosuccinate synthetase-like

[tnl:113504874](https://www.genome.jp/entry/tnl:113504874) [K01254](https://www.genome.jp/entry/K01254) leukotriene A-4 hydrolase isoform X1

[tnl:113504890](https://www.genome.jp/entry/tnl:113504890) [K01697](https://www.genome.jp/entry/K01697) cystathionine beta-synthase-like

[tnl:113504959](https://www.genome.jp/entry/tnl:113504959) [K05901](https://www.genome.jp/entry/K05901) flavin reductase (NADPH)

[tnl:113505041](https://www.genome.jp/entry/tnl:113505041) [K01116](https://www.genome.jp/entry/K01116) 1-phosphatidylinositol 4,5-bisphosphate phosphodiesterase gamma-1

[tnl:113505102](https://www.genome.jp/entry/tnl:113505102) [K05546](https://www.genome.jp/entry/K05546) neutral alpha-glucosidase AB-like isoform X1

[tnl:113505248](https://www.genome.jp/entry/tnl:113505248) [K01518](https://www.genome.jp/entry/K01518) bis(5'-nucleosyl)-tetraphosphatase [asymmetrical]

[tnl:113505283](https://www.genome.jp/entry/tnl:113505283) [K20730](https://www.genome.jp/entry/K20730) probable beta-hexosaminidase fdl

[tnl:113505327](https://www.genome.jp/entry/tnl:113505327) [K13800](https://www.genome.jp/entry/K13800) UMP-CMP kinase

[tnl:113505357](https://www.genome.jp/entry/tnl:113505357) [K00357](https://www.genome.jp/entry/K00357) dihydropteridine reductase

[tnl:113505484](https://www.genome.jp/entry/tnl:113505484) [K03662](https://www.genome.jp/entry/K03662) V-type proton ATPase subunit S1

[tnl:113505697](https://www.genome.jp/entry/tnl:113505697) [K01648](https://www.genome.jp/entry/K01648) ATP-citrate synthase

[tnl:113505855](https://www.genome.jp/entry/tnl:113505855) [K01809](https://www.genome.jp/entry/K01809) mannose-6-phosphate isomerase

[tnl:113506141](https://www.genome.jp/entry/tnl:113506141) [K07964](https://www.genome.jp/entry/K07964) heparanase-like

[tnl:113506144](https://www.genome.jp/entry/tnl:113506144) [K01255](https://www.genome.jp/entry/K01255) putative aminopeptidase W07G4.4

[tnl:113506203](https://www.genome.jp/entry/tnl:113506203) [K07509](https://www.genome.jp/entry/K07509) trifunctional enzyme subunit beta, mitochondrial-like

[tnl:113506207](https://www.genome.jp/entry/tnl:113506207) [K02152](https://www.genome.jp/entry/K02152) V-type proton ATPase subunit G

[tnl:113506529](https://www.genome.jp/entry/tnl:113506529) [K01834](https://www.genome.jp/entry/K01834) phosphoglycerate mutase 2-like

[tnl:113506768](https://www.genome.jp/entry/tnl:113506768) [K05546](https://www.genome.jp/entry/K05546) LOW QUALITY PROTEIN: neutral alpha-glucosidase AB-like

[tnl:113506824](https://www.genome.jp/entry/tnl:113506824) [K15734](https://www.genome.jp/entry/K15734) short-chain dehydrogenase/reductase family 16C member 6-like

[tnl:113506886](https://www.genome.jp/entry/tnl:113506886) [K01952](https://www.genome.jp/entry/K01952) LOW QUALITY PROTEIN: phosphoribosylformylglycinamidine synthase-like

[tnl:113506948](https://www.genome.jp/entry/tnl:113506948) [K00016](https://www.genome.jp/entry/K00016) L-lactate dehydrogenase-like isoform X1

[tnl:113507103](https://www.genome.jp/entry/tnl:113507103) [K00799](https://www.genome.jp/entry/K00799) glutathione S-transferase 1-1-like

[tnl:113507140](https://www.genome.jp/entry/tnl:113507140) [K00049](https://www.genome.jp/entry/K00049) LOW QUALITY PROTEIN: glyoxylate reductase/hydroxypyruvate reductase-like

[tnl:113507470](https://www.genome.jp/entry/tnl:113507470) [K01874](https://www.genome.jp/entry/K01874) methionine--tRNA ligase, cytoplasmic

[tnl:113507591](https://www.genome.jp/entry/tnl:113507591) [K15717](https://www.genome.jp/entry/K15717) prostamide/prostaglandin F synthase-like isoform X1

[tnl:113507614](https://www.genome.jp/entry/tnl:113507614) [K00923](https://www.genome.jp/entry/K00923) phosphatidylinositol 4-phosphate 3-kinase C2 domain-containing subunit alpha isoform X1

[tnl:113507645](https://www.genome.jp/entry/tnl:113507645) [K11214](https://www.genome.jp/entry/K11214) sedoheptulokinase-like

[tnl:113507725](https://www.genome.jp/entry/tnl:113507725) [K01937](https://www.genome.jp/entry/K01937) CTP synthase

[tnl:113507735](https://www.genome.jp/entry/tnl:113507735) [K15734](https://www.genome.jp/entry/K15734) short-chain dehydrogenase/reductase family 16C member 6-like isoform X1

[tnl:113507745](https://www.genome.jp/entry/tnl:113507745) [K00710](https://www.genome.jp/entry/K00710) polypeptide N-acetylgalactosaminyltransferase 5

[tnl:113507884](https://www.genome.jp/entry/tnl:113507884) [K00249](https://www.genome.jp/entry/K00249) probable medium-chain specific acyl-CoA dehydrogenase, mitochondrial isoform X1

[tnl:113507902](https://www.genome.jp/entry/tnl:113507902) [K00968](https://www.genome.jp/entry/K00968) choline-phosphate cytidylyltransferase B-like isoform X1

[tnl:113507917](https://www.genome.jp/entry/tnl:113507917) [K00012](https://www.genome.jp/entry/K00012) UDP-glucose 6-dehydrogenase

[tnl:113507969](https://www.genome.jp/entry/tnl:113507969) [K01939](https://www.genome.jp/entry/K01939) LOW QUALITY PROTEIN: adenylosuccinate synthetase-like

[tnl:113507970](https://www.genome.jp/entry/tnl:113507970) [K08049](https://www.genome.jp/entry/K08049) adenylate cyclase type 9

[tnl:113508032](https://www.genome.jp/entry/tnl:113508032) [K01082](https://www.genome.jp/entry/K01082) 3'(2'),5'-bisphosphate nucleotidase 1-like isoform X1

[tnl:113508050](https://www.genome.jp/entry/tnl:113508050) [K00799](https://www.genome.jp/entry/K00799) glutathione S-transferase 1-1-like

[tnl:113508098](https://www.genome.jp/entry/tnl:113508098) [K17497](https://www.genome.jp/entry/K17497) phosphomannomutase 2

[tnl:113508324](https://www.genome.jp/entry/tnl:113508324) [K00616](https://www.genome.jp/entry/K00616) probable transaldolase

[tnl:113508342](https://www.genome.jp/entry/tnl:113508342) [K01807](https://www.genome.jp/entry/K01807) ribose-5-phosphate isomerase

[tnl:113508348](https://www.genome.jp/entry/tnl:113508348) [K01599](https://www.genome.jp/entry/K01599) uroporphyrinogen decarboxylase

[tnl:113508384](https://www.genome.jp/entry/tnl:113508384) [K01886](https://www.genome.jp/entry/K01886) probable glutamine--tRNA ligase

[tnl:113508501](https://www.genome.jp/entry/tnl:113508501) [K00560](https://www.genome.jp/entry/K00560) thymidylate synthase

[tnl:113508532](https://www.genome.jp/entry/tnl:113508532) [K00088](https://www.genome.jp/entry/K00088) inosine-5'-monophosphate dehydrogenase

[tnl:113508579](https://www.genome.jp/entry/tnl:113508579) [K24242](https://www.genome.jp/entry/K24242) 7-methylguanosine phosphate-specific 5'-nucleotidase

[tnl:113508734](https://www.genome.jp/entry/tnl:113508734) [K01194](https://www.genome.jp/entry/K01194) uncharacterized protein LOC113508734

[tnl:113508887](https://www.genome.jp/entry/tnl:113508887) [K00700](https://www.genome.jp/entry/K00700) 1,4-alpha-glucan-branching enzyme

[tnl:113509001](https://www.genome.jp/entry/tnl:113509001) [K00134](https://www.genome.jp/entry/K00134) glyceraldehyde-3-phosphate dehydrogenase

 [**tnl04144**](https://www.genome.jp/kegg-bin/show_pathway?166859291342340/tnl04144.args) **Endocytosis - Trichoplusia ni (cabbage looper) (**[**86**](javascript:display('tnl04144'))**)**

[tnl:113491861](https://www.genome.jp/entry/tnl:113491861) [K12195](https://www.genome.jp/entry/K12195) charged multivesicular body protein 6-A

[tnl:113491887](https://www.genome.jp/entry/tnl:113491887) [K04678](https://www.genome.jp/entry/K04678) E3 ubiquitin-protein ligase SMURF2

[tnl:113491902](https://www.genome.jp/entry/tnl:113491902) [K12182](https://www.genome.jp/entry/K12182) hepatocyte growth factor-regulated tyrosine kinase substrate

[tnl:113492238](https://www.genome.jp/entry/tnl:113492238) [K12471](https://www.genome.jp/entry/K12471) epsin-1

[tnl:113492259](https://www.genome.jp/entry/tnl:113492259) [K15053](https://www.genome.jp/entry/K15053) charged multivesicular body protein 7 isoform X1

[tnl:113492527](https://www.genome.jp/entry/tnl:113492527) [K01528](https://www.genome.jp/entry/K01528) LOW QUALITY PROTEIN: dynamin-like

[tnl:113492607](https://www.genome.jp/entry/tnl:113492607) [K07897](https://www.genome.jp/entry/K07897) ras-related protein Rab-7a

[tnl:113493049](https://www.genome.jp/entry/tnl:113493049) [K23612](https://www.genome.jp/entry/K23612) neural Wiskott-Aldrich syndrome protein-like

[tnl:113493088](https://www.genome.jp/entry/tnl:113493088) [K07939](https://www.genome.jp/entry/K07939) ADP-ribosylation factor 2

[tnl:113493098](https://www.genome.jp/entry/tnl:113493098) [K04705](https://www.genome.jp/entry/K04705) signal transducing adapter molecule 1

[tnl:113493341](https://www.genome.jp/entry/tnl:113493341) [K12200](https://www.genome.jp/entry/K12200) programmed cell death 6-interacting protein

[tnl:113493786](https://www.genome.jp/entry/tnl:113493786) [K07876](https://www.genome.jp/entry/K07876) ras-related protein Rab-35

[tnl:113493842](https://www.genome.jp/entry/tnl:113493842) [K04646](https://www.genome.jp/entry/K04646) clathrin heavy chain isoform X1

[tnl:113494124](https://www.genome.jp/entry/tnl:113494124) [K06093](https://www.genome.jp/entry/K06093) partitioning defective protein 6-like

[tnl:113494261](https://www.genome.jp/entry/tnl:113494261) [K12486](https://www.genome.jp/entry/K12486) LOW QUALITY PROTEIN: stromal membrane-associated protein 1-like

[tnl:113494390](https://www.genome.jp/entry/tnl:113494390) [K18468](https://www.genome.jp/entry/K18468) vacuolar protein sorting-associated protein 35 isoform X1

[tnl:113494425](https://www.genome.jp/entry/tnl:113494425) [K12184](https://www.genome.jp/entry/K12184) vacuolar protein sorting-associated protein 28 homolog

[tnl:113494446](https://www.genome.jp/entry/tnl:113494446) [K12185](https://www.genome.jp/entry/K12185) vacuolar protein sorting-associated protein 37C

[tnl:113494603](https://www.genome.jp/entry/tnl:113494603) [K12186](https://www.genome.jp/entry/K12186) uncharacterized protein LOC113494603

[tnl:113494939](https://www.genome.jp/entry/tnl:113494939) [K12193](https://www.genome.jp/entry/K12193) charged multivesicular body protein 3

[tnl:113495053](https://www.genome.jp/entry/tnl:113495053) [K12199](https://www.genome.jp/entry/K12199) vacuolar protein sorting-associated protein VTA1 homolog

[tnl:113495152](https://www.genome.jp/entry/tnl:113495152) [K08291](https://www.genome.jp/entry/K08291) G protein-coupled receptor kinase 2 isoform X1

[tnl:113495560](https://www.genome.jp/entry/tnl:113495560) [K03283](https://www.genome.jp/entry/K03283) heat shock protein 68-like

[tnl:113495620](https://www.genome.jp/entry/tnl:113495620) [K04393](https://www.genome.jp/entry/K04393) cdc42 homolog

[tnl:113495991](https://www.genome.jp/entry/tnl:113495991) [K12479](https://www.genome.jp/entry/K12479) vacuolar protein sorting-associated protein 45

[tnl:113496267](https://www.genome.jp/entry/tnl:113496267) [K03283](https://www.genome.jp/entry/K03283) heat shock protein 68-like

[tnl:113496317](https://www.genome.jp/entry/tnl:113496317) [K12488](https://www.genome.jp/entry/K12488) arfGAP with SH3 domain, ANK repeat and PH domain-containing protein isoform X1

[tnl:113496575](https://www.genome.jp/entry/tnl:113496575) [K12196](https://www.genome.jp/entry/K12196) vacuolar protein sorting-associated protein 4A-like

[tnl:113496612](https://www.genome.jp/entry/tnl:113496612) [K12484](https://www.genome.jp/entry/K12484) rab11 family-interacting protein 2-like

[tnl:113496854](https://www.genome.jp/entry/tnl:113496854) [K07904](https://www.genome.jp/entry/K07904) ras-related protein Rab-11A

[tnl:113497265](https://www.genome.jp/entry/tnl:113497265) [K05704](https://www.genome.jp/entry/K05704) tyrosine-protein kinase Src64B isoform X1

[tnl:113498012](https://www.genome.jp/entry/tnl:113498012) [K18442](https://www.genome.jp/entry/K18442) brefeldin A-inhibited guanine nucleotide-exchange protein 1-like isoform X1

[tnl:113498046](https://www.genome.jp/entry/tnl:113498046) [K05757](https://www.genome.jp/entry/K05757) actin-related protein 2/3 complex subunit 1A-B-like

[tnl:113498318](https://www.genome.jp/entry/tnl:113498318) [K07903](https://www.genome.jp/entry/K07903) ras-related protein Rab-10

[tnl:113498581](https://www.genome.jp/entry/tnl:113498581) [K11824](https://www.genome.jp/entry/K11824) AP-2 complex subunit alpha

[tnl:113498602](https://www.genome.jp/entry/tnl:113498602) [K11826](https://www.genome.jp/entry/K11826) AP-2 complex subunit mu

[tnl:113498610](https://www.genome.jp/entry/tnl:113498610) [K12489](https://www.genome.jp/entry/K12489) arf-GAP with coiled-coil, ANK repeat and PH domain-containing protein 2

[tnl:113498774](https://www.genome.jp/entry/tnl:113498774) [K12185](https://www.genome.jp/entry/K12185) vacuolar protein sorting-associated protein 37A

[tnl:113498854](https://www.genome.jp/entry/tnl:113498854) [K07937](https://www.genome.jp/entry/K07937) ADP-ribosylation factor 1

[tnl:113499032](https://www.genome.jp/entry/tnl:113499032) [K00910](https://www.genome.jp/entry/K00910) G protein-coupled receptor kinase 1 isoform X1

[tnl:113499115](https://www.genome.jp/entry/tnl:113499115) [K12192](https://www.genome.jp/entry/K12192) charged multivesicular body protein 2b isoform X1

[tnl:113499233](https://www.genome.jp/entry/tnl:113499233) [K10365](https://www.genome.jp/entry/K10365) F-actin-capping protein subunit beta-like

[tnl:113499278](https://www.genome.jp/entry/tnl:113499278) [K12194](https://www.genome.jp/entry/K12194) charged multivesicular body protein 4b

[tnl:113499725](https://www.genome.jp/entry/tnl:113499725) [K12483](https://www.genome.jp/entry/K12483) EH domain-containing protein 3

[tnl:113499878](https://www.genome.jp/entry/tnl:113499878) [K17918](https://www.genome.jp/entry/K17918) sorting nexin-12

[tnl:113499893](https://www.genome.jp/entry/tnl:113499893) [K12188](https://www.genome.jp/entry/K12188) vacuolar-sorting protein SNF8

[tnl:113500324](https://www.genome.jp/entry/tnl:113500324) [K12493](https://www.genome.jp/entry/K12493) ADP-ribosylation factor GTPase-activating protein 3

[tnl:113500328](https://www.genome.jp/entry/tnl:113500328) [K12191](https://www.genome.jp/entry/K12191) charged multivesicular body protein 2a

[tnl:113500413](https://www.genome.jp/entry/tnl:113500413) [K12562](https://www.genome.jp/entry/K12562) myc box-dependent-interacting protein 1 isoform X1

[tnl:113500418](https://www.genome.jp/entry/tnl:113500418) [K12198](https://www.genome.jp/entry/K12198) charged multivesicular body protein 5

[tnl:113500540](https://www.genome.jp/entry/tnl:113500540) [K17919](https://www.genome.jp/entry/K17919) sorting nexin-4-like

[tnl:113500727](https://www.genome.jp/entry/tnl:113500727) [K11866](https://www.genome.jp/entry/K11866) STAM-binding protein-like A

[tnl:113500805](https://www.genome.jp/entry/tnl:113500805) [K12494](https://www.genome.jp/entry/K12494) PH and SEC7 domain-containing protein 1

[tnl:113501046](https://www.genome.jp/entry/tnl:113501046) [K12485](https://www.genome.jp/entry/K12485) rab11 family-interacting protein 4 isoform X1

[tnl:113501223](https://www.genome.jp/entry/tnl:113501223) [K12472](https://www.genome.jp/entry/K12472) epidermal growth factor receptor substrate 15-like 1

[tnl:113501473](https://www.genome.jp/entry/tnl:113501473) [K05754](https://www.genome.jp/entry/K05754) actin-related protein 2/3 complex subunit 5-B

[tnl:113501562](https://www.genome.jp/entry/tnl:113501562) [K05633](https://www.genome.jp/entry/K05633) E3 ubiquitin-protein ligase Su(dx)

[tnl:113502027](https://www.genome.jp/entry/tnl:113502027) [K04439](https://www.genome.jp/entry/K04439) arrestin red cell isoform X1

[tnl:113502068](https://www.genome.jp/entry/tnl:113502068) [K07941](https://www.genome.jp/entry/K07941) ADP-ribosylation factor 6

[tnl:113502180](https://www.genome.jp/entry/tnl:113502180) [K12480](https://www.genome.jp/entry/K12480) rab GTPase-binding effector protein 1 isoform X1

[tnl:113502258](https://www.genome.jp/entry/tnl:113502258) [K17920](https://www.genome.jp/entry/K17920) sorting nexin-6

[tnl:113502273](https://www.genome.jp/entry/tnl:113502273) [K12197](https://www.genome.jp/entry/K12197) charged multivesicular body protein 1b isoform X1

[tnl:113502320](https://www.genome.jp/entry/tnl:113502320) [K10364](https://www.genome.jp/entry/K10364) F-actin-capping protein subunit alpha

[tnl:113503427](https://www.genome.jp/entry/tnl:113503427) [K04513](https://www.genome.jp/entry/K04513) ras-like GTP-binding protein Rho1 isoform X1

[tnl:113503473](https://www.genome.jp/entry/tnl:113503473) [K11247](https://www.genome.jp/entry/K11247) endophilin-A isoform X1

[tnl:113503760](https://www.genome.jp/entry/tnl:113503760) [K12475](https://www.genome.jp/entry/K12475) protein disabled-like isoform X1

[tnl:113503799](https://www.genome.jp/entry/tnl:113503799) [K12492](https://www.genome.jp/entry/K12492) ADP-ribosylation factor GTPase-activating protein 1-like

[tnl:113503941](https://www.genome.jp/entry/tnl:113503941) [K12492](https://www.genome.jp/entry/K12492) uncharacterized protein LOC113503941

[tnl:113504269](https://www.genome.jp/entry/tnl:113504269) [K00889](https://www.genome.jp/entry/K00889) phosphatidylinositol 4-phosphate 5-kinase type-1 alpha-like isoform X1

[tnl:113504416](https://www.genome.jp/entry/tnl:113504416) [K18584](https://www.genome.jp/entry/K18584) actin-related protein 3

[tnl:113504452](https://www.genome.jp/entry/tnl:113504452) [K12487](https://www.genome.jp/entry/K12487) ARF GTPase-activating protein GIT2

[tnl:113504608](https://www.genome.jp/entry/tnl:113504608) [K10396](https://www.genome.jp/entry/K10396) kinesin heavy chain

[tnl:113504885](https://www.genome.jp/entry/tnl:113504885) [K17917](https://www.genome.jp/entry/K17917) sorting nexin-2 isoform X1

[tnl:113505027](https://www.genome.jp/entry/tnl:113505027) [K07901](https://www.genome.jp/entry/K07901) ras-related protein Rab-8A isoform X1

[tnl:113505141](https://www.genome.jp/entry/tnl:113505141) [K18466](https://www.genome.jp/entry/K18466) vacuolar protein sorting-associated protein 26B-like

[tnl:113505180](https://www.genome.jp/entry/tnl:113505180) [K04707](https://www.genome.jp/entry/K04707) E3 ubiquitin-protein ligase CBL-B-B isoform X1

[tnl:113505189](https://www.genome.jp/entry/tnl:113505189) [K04674](https://www.genome.jp/entry/K04674) TGF-beta receptor type-1-like isoform X1

[tnl:113505197](https://www.genome.jp/entry/tnl:113505197) [K17260](https://www.genome.jp/entry/K17260) actin-related protein 2

[tnl:113505472](https://www.genome.jp/entry/tnl:113505472) [K12183](https://www.genome.jp/entry/K12183) tumor susceptibility gene 101 protein

[tnl:113506351](https://www.genome.jp/entry/tnl:113506351) [K04237](https://www.genome.jp/entry/K04237) partitioning defective 3 homolog isoform X1

[tnl:113506423](https://www.genome.jp/entry/tnl:113506423) [K03283](https://www.genome.jp/entry/K03283) heat shock 70 kDa protein cognate 4

[tnl:113506525](https://www.genome.jp/entry/tnl:113506525) [K18467](https://www.genome.jp/entry/K18467) vacuolar protein sorting-associated protein 29

[tnl:113506940](https://www.genome.jp/entry/tnl:113506940) [K18442](https://www.genome.jp/entry/K18442) LOW QUALITY PROTEIN: brefeldin A-inhibited guanine nucleotide-exchange protein 1-like

[tnl:113506942](https://www.genome.jp/entry/tnl:113506942) [K05757](https://www.genome.jp/entry/K05757) LOW QUALITY PROTEIN: actin-related protein 2/3 complex subunit 1A-B-like

[tnl:113507118](https://www.genome.jp/entry/tnl:113507118) [K01528](https://www.genome.jp/entry/K01528) LOW QUALITY PROTEIN: dynamin-like

[tnl:113508447](https://www.genome.jp/entry/tnl:113508447) [K12190](https://www.genome.jp/entry/K12190) vacuolar protein-sorting-associated protein 36

 [**tnl03010**](https://www.genome.jp/kegg-bin/show_pathway?166859291342340/tnl03010.args) **Ribosome - Trichoplusia ni (cabbage looper) (**[**74**](javascript:display('tnl03010'))**)**

[tnl:113492011](https://www.genome.jp/entry/tnl:113492011) [K02998](https://www.genome.jp/entry/K02998) 40S ribosomal protein SA

[tnl:113492046](https://www.genome.jp/entry/tnl:113492046) [K02905](https://www.genome.jp/entry/K02905) 60S ribosomal protein L29

[tnl:113492511](https://www.genome.jp/entry/tnl:113492511) [K02932](https://www.genome.jp/entry/K02932) 60S ribosomal protein L5

[tnl:113492600](https://www.genome.jp/entry/tnl:113492600) [K02989](https://www.genome.jp/entry/K02989) 40S ribosomal protein S5

[tnl:113492673](https://www.genome.jp/entry/tnl:113492673) [K02908](https://www.genome.jp/entry/K02908) 60S ribosomal protein L30

[tnl:113492691](https://www.genome.jp/entry/tnl:113492691) [K02938](https://www.genome.jp/entry/K02938) 60S ribosomal protein L8

[tnl:113492822](https://www.genome.jp/entry/tnl:113492822) [K02868](https://www.genome.jp/entry/K02868) 60S ribosomal protein L11

[tnl:113492881](https://www.genome.jp/entry/tnl:113492881) [K02995](https://www.genome.jp/entry/K02995) 40S ribosomal protein S8

[tnl:113492909](https://www.genome.jp/entry/tnl:113492909) [K02865](https://www.genome.jp/entry/K02865) 60S ribosomal protein L10a

[tnl:113492912](https://www.genome.jp/entry/tnl:113492912) [K02942](https://www.genome.jp/entry/K02942) 60S acidic ribosomal protein P1

[tnl:113492956](https://www.genome.jp/entry/tnl:113492956) [K02941](https://www.genome.jp/entry/K02941) 60S acidic ribosomal protein P0

[tnl:113493012](https://www.genome.jp/entry/tnl:113493012) [K02917](https://www.genome.jp/entry/K02917) 60S ribosomal protein L35a

[tnl:113493481](https://www.genome.jp/entry/tnl:113493481) [K02964](https://www.genome.jp/entry/K02964) 40S ribosomal protein S18

[tnl:113493830](https://www.genome.jp/entry/tnl:113493830) [K02920](https://www.genome.jp/entry/K02920) 60S ribosomal protein L36

[tnl:113494180](https://www.genome.jp/entry/tnl:113494180) [K02937](https://www.genome.jp/entry/K02937) 60S ribosomal protein L7

[tnl:113494209](https://www.genome.jp/entry/tnl:113494209) [K02993](https://www.genome.jp/entry/K02993) 40S ribosomal protein S7

[tnl:113494440](https://www.genome.jp/entry/tnl:113494440) [K02969](https://www.genome.jp/entry/K02969) 40S ribosomal protein S20

[tnl:113494564](https://www.genome.jp/entry/tnl:113494564) [K02936](https://www.genome.jp/entry/K02936) 60S ribosomal protein L7a

[tnl:113494599](https://www.genome.jp/entry/tnl:113494599) [K02973](https://www.genome.jp/entry/K02973) 40S ribosomal protein S23

[tnl:113494829](https://www.genome.jp/entry/tnl:113494829) [K02974](https://www.genome.jp/entry/K02974) 40S ribosomal protein S24

[tnl:113495020](https://www.genome.jp/entry/tnl:113495020) [K02891](https://www.genome.jp/entry/K02891) 60S ribosomal protein L22-like

[tnl:113495374](https://www.genome.jp/entry/tnl:113495374) [K02987](https://www.genome.jp/entry/K02987) 40S ribosomal protein S4

[tnl:113496638](https://www.genome.jp/entry/tnl:113496638) [K02885](https://www.genome.jp/entry/K02885) 60S ribosomal protein L19

[tnl:113496656](https://www.genome.jp/entry/tnl:113496656) [K02875](https://www.genome.jp/entry/K02875) 60S ribosomal protein L14

[tnl:113497028](https://www.genome.jp/entry/tnl:113497028) [K02873](https://www.genome.jp/entry/K02873) 60S ribosomal protein L13

[tnl:113497181](https://www.genome.jp/entry/tnl:113497181) [K02929](https://www.genome.jp/entry/K02929) 60S ribosomal protein L44

[tnl:113497792](https://www.genome.jp/entry/tnl:113497792) [K02940](https://www.genome.jp/entry/K02940) 60S ribosomal protein L9

[tnl:113498059](https://www.genome.jp/entry/tnl:113498059) [K02979](https://www.genome.jp/entry/K02979) 40S ribosomal protein S28

[tnl:113498267](https://www.genome.jp/entry/tnl:113498267) [K02882](https://www.genome.jp/entry/K02882) 60S ribosomal protein L18a

[tnl:113498677](https://www.genome.jp/entry/tnl:113498677) [K02984](https://www.genome.jp/entry/K02984) 40S ribosomal protein S3a

[tnl:113499276](https://www.genome.jp/entry/tnl:113499276) [K02949](https://www.genome.jp/entry/K02949) 40S ribosomal protein S11 isoform X1

[tnl:113499356](https://www.genome.jp/entry/tnl:113499356) [K02978](https://www.genome.jp/entry/K02978) 40S ribosomal protein S27

[tnl:113499812](https://www.genome.jp/entry/tnl:113499812) [K02903](https://www.genome.jp/entry/K02903) 60S ribosomal protein L28

[tnl:113500060](https://www.genome.jp/entry/tnl:113500060) [K02953](https://www.genome.jp/entry/K02953) 40S ribosomal protein S13

[tnl:113500369](https://www.genome.jp/entry/tnl:113500369) [K02960](https://www.genome.jp/entry/K02960) 40S ribosomal protein S16 isoform X1

[tnl:113500782](https://www.genome.jp/entry/tnl:113500782) [K02955](https://www.genome.jp/entry/K02955) 40S ribosomal protein S14

[tnl:113500785](https://www.genome.jp/entry/tnl:113500785) [K02896](https://www.genome.jp/entry/K02896) 60S ribosomal protein L24

[tnl:113501556](https://www.genome.jp/entry/tnl:113501556) [K02918](https://www.genome.jp/entry/K02918) 60S ribosomal protein L35

[tnl:113502023](https://www.genome.jp/entry/tnl:113502023) [K02997](https://www.genome.jp/entry/K02997) 40S ribosomal protein S9

[tnl:113502118](https://www.genome.jp/entry/tnl:113502118) [K02901](https://www.genome.jp/entry/K02901) 60S ribosomal protein L27-like

[tnl:113502613](https://www.genome.jp/entry/tnl:113502613) [K02922](https://www.genome.jp/entry/K02922) 60S ribosomal protein L37

[tnl:113502867](https://www.genome.jp/entry/tnl:113502867) [K02943](https://www.genome.jp/entry/K02943) 60S acidic ribosomal protein P2-like

[tnl:113502978](https://www.genome.jp/entry/tnl:113502978) [K02985](https://www.genome.jp/entry/K02985) 40S ribosomal protein S3

[tnl:113503104](https://www.genome.jp/entry/tnl:113503104) [K02934](https://www.genome.jp/entry/K02934) 60S ribosomal protein L6

[tnl:113503306](https://www.genome.jp/entry/tnl:113503306) [K02991](https://www.genome.jp/entry/K02991) 40S ribosomal protein S6

[tnl:113503318](https://www.genome.jp/entry/tnl:113503318) [K02880](https://www.genome.jp/entry/K02880) 60S ribosomal protein L17

[tnl:113503424](https://www.genome.jp/entry/tnl:113503424) [K02958](https://www.genome.jp/entry/K02958) 40S ribosomal protein S15

[tnl:113503678](https://www.genome.jp/entry/tnl:113503678) [K02915](https://www.genome.jp/entry/K02915) 60S ribosomal protein L34-like

[tnl:113503792](https://www.genome.jp/entry/tnl:113503792) [K02976](https://www.genome.jp/entry/K02976) 40S ribosomal protein S26

[tnl:113504069](https://www.genome.jp/entry/tnl:113504069) [K02894](https://www.genome.jp/entry/K02894) 60S ribosomal protein L23

[tnl:113504531](https://www.genome.jp/entry/tnl:113504531) [K02866](https://www.genome.jp/entry/K02866) 60S ribosomal protein L10

[tnl:113505380](https://www.genome.jp/entry/tnl:113505380) [K02983](https://www.genome.jp/entry/K02983) ubiquitin-like protein FUBI

[tnl:113505482](https://www.genome.jp/entry/tnl:113505482) [K02870](https://www.genome.jp/entry/K02870) 60S ribosomal protein L12

[tnl:113505587](https://www.genome.jp/entry/tnl:113505587) [K02912](https://www.genome.jp/entry/K02912) 60S ribosomal protein L32

[tnl:113505787](https://www.genome.jp/entry/tnl:113505787) [K02877](https://www.genome.jp/entry/K02877) 60S ribosomal protein L15

[tnl:113505833](https://www.genome.jp/entry/tnl:113505833) [K02898](https://www.genome.jp/entry/K02898) 60S ribosomal protein L26

[tnl:113506374](https://www.genome.jp/entry/tnl:113506374) [K02977](https://www.genome.jp/entry/K02977) ubiquitin-40S ribosomal protein S27a

[tnl:113506555](https://www.genome.jp/entry/tnl:113506555) [K02962](https://www.genome.jp/entry/K02962) 40S ribosomal protein S17

[tnl:113506697](https://www.genome.jp/entry/tnl:113506697) [K02980](https://www.genome.jp/entry/K02980) 40S ribosomal protein S29

[tnl:113506748](https://www.genome.jp/entry/tnl:113506748) [K02975](https://www.genome.jp/entry/K02975) 40S ribosomal protein S25

[tnl:113506956](https://www.genome.jp/entry/tnl:113506956) [K02925](https://www.genome.jp/entry/K02925) 60S ribosomal protein L3

[tnl:113507000](https://www.genome.jp/entry/tnl:113507000) [K02893](https://www.genome.jp/entry/K02893) 60S ribosomal protein L23a

[tnl:113507148](https://www.genome.jp/entry/tnl:113507148) [K02957](https://www.genome.jp/entry/K02957) 40S ribosomal protein S15Aa

[tnl:113507345](https://www.genome.jp/entry/tnl:113507345) [K02981](https://www.genome.jp/entry/K02981) 40S ribosomal protein S2

[tnl:113507539](https://www.genome.jp/entry/tnl:113507539) [K02951](https://www.genome.jp/entry/K02951) 40S ribosomal protein S12-like

[tnl:113507742](https://www.genome.jp/entry/tnl:113507742) [K02921](https://www.genome.jp/entry/K02921) 60S ribosomal protein L37a

[tnl:113507777](https://www.genome.jp/entry/tnl:113507777) [K02872](https://www.genome.jp/entry/K02872) 60S ribosomal protein L13a

[tnl:113508086](https://www.genome.jp/entry/tnl:113508086) [K02923](https://www.genome.jp/entry/K02923) 60S ribosomal protein L38

[tnl:113508227](https://www.genome.jp/entry/tnl:113508227) [K02947](https://www.genome.jp/entry/K02947) 40S ribosomal protein S10

[tnl:113508390](https://www.genome.jp/entry/tnl:113508390) [K02924](https://www.genome.jp/entry/K02924) 60S ribosomal protein L39

[tnl:113508400](https://www.genome.jp/entry/tnl:113508400) [K02910](https://www.genome.jp/entry/K02910) 60S ribosomal protein L31

[tnl:113508567](https://www.genome.jp/entry/tnl:113508567) [K02889](https://www.genome.jp/entry/K02889) 60S ribosomal protein L21

[tnl:113508582](https://www.genome.jp/entry/tnl:113508582) [K02900](https://www.genome.jp/entry/K02900) 60S ribosomal protein L27a

[tnl:113508986](https://www.genome.jp/entry/tnl:113508986) [K02930](https://www.genome.jp/entry/K02930) 60S ribosomal protein L4

 [**tnl04141**](https://www.genome.jp/kegg-bin/show_pathway?166859291342340/tnl04141.args) **Protein processing in endoplasmic reticulum - Trichoplusia ni (cabbage looper) (**[**50**](javascript:display('tnl04141'))**)**

[tnl:113492644](https://www.genome.jp/entry/tnl:113492644) [K09525](https://www.genome.jp/entry/K09525) dnaJ homolog subfamily C member 5

[tnl:113493815](https://www.genome.jp/entry/tnl:113493815) [K14016](https://www.genome.jp/entry/K14016) ubiquitin fusion degradation protein 1 homolog isoform X1

[tnl:113494236](https://www.genome.jp/entry/tnl:113494236) [K03237](https://www.genome.jp/entry/K03237) eukaryotic translation initiation factor 2 subunit 1

[tnl:113494585](https://www.genome.jp/entry/tnl:113494585) [K04431](https://www.genome.jp/entry/K04431) dual specificity mitogen-activated protein kinase kinase 7-like isoform X1

[tnl:113494618](https://www.genome.jp/entry/tnl:113494618) [K09490](https://www.genome.jp/entry/K09490) endoplasmic reticulum chaperone BiP

[tnl:113494695](https://www.genome.jp/entry/tnl:113494695) [K09561](https://www.genome.jp/entry/K09561) E3 ubiquitin-protein ligase CHIP

[tnl:113494870](https://www.genome.jp/entry/tnl:113494870) [K14015](https://www.genome.jp/entry/K14015) nuclear protein localization protein 4 homolog isoform X1

[tnl:113495560](https://www.genome.jp/entry/tnl:113495560) [K03283](https://www.genome.jp/entry/K03283) heat shock protein 68-like

[tnl:113495605](https://www.genome.jp/entry/tnl:113495605) [K09562](https://www.genome.jp/entry/K09562) hsp70-binding protein 1

[tnl:113495987](https://www.genome.jp/entry/tnl:113495987) [K14012](https://www.genome.jp/entry/K14012) NSFL1 cofactor p47

[tnl:113496266](https://www.genome.jp/entry/tnl:113496266) [K04079](https://www.genome.jp/entry/K04079) heat shock protein 83

[tnl:113496267](https://www.genome.jp/entry/tnl:113496267) [K03283](https://www.genome.jp/entry/K03283) heat shock protein 68-like

[tnl:113496346](https://www.genome.jp/entry/tnl:113496346) [K12667](https://www.genome.jp/entry/K12667) dolichyl-diphosphooligosaccharide--protein glycosyltransferase subunit 2

[tnl:113497282](https://www.genome.jp/entry/tnl:113497282) [K24348](https://www.genome.jp/entry/K24348) UBX domain-containing protein 1

[tnl:113497745](https://www.genome.jp/entry/tnl:113497745) [K06689](https://www.genome.jp/entry/K06689) ubiquitin-conjugating enzyme E2-17 kDa

[tnl:113497802](https://www.genome.jp/entry/tnl:113497802) [K14005](https://www.genome.jp/entry/K14005) protein transport protein Sec31A isoform X1

[tnl:113498138](https://www.genome.jp/entry/tnl:113498138) [K09542](https://www.genome.jp/entry/K09542) protein lethal(2)essential for life-like

[tnl:113498362](https://www.genome.jp/entry/tnl:113498362) [K14007](https://www.genome.jp/entry/K14007) protein transport protein Sec24C isoform X1

[tnl:113499144](https://www.genome.jp/entry/tnl:113499144) [K14011](https://www.genome.jp/entry/K14011) UBX domain-containing protein 6 isoform X1

[tnl:113500159](https://www.genome.jp/entry/tnl:113500159) [K10080](https://www.genome.jp/entry/K10080) protein ERGIC-53

[tnl:113500490](https://www.genome.jp/entry/tnl:113500490) [K09502](https://www.genome.jp/entry/K09502) dnaJ homolog subfamily A member 1

[tnl:113500546](https://www.genome.jp/entry/tnl:113500546) [K13719](https://www.genome.jp/entry/K13719) ubiquitin thioesterase OTU1

[tnl:113500780](https://www.genome.jp/entry/tnl:113500780) [K07953](https://www.genome.jp/entry/K07953) GTP-binding protein SAR1b

[tnl:113501015](https://www.genome.jp/entry/tnl:113501015) [K09503](https://www.genome.jp/entry/K09503) dnaJ homolog subfamily A member 2-like

[tnl:113501048](https://www.genome.jp/entry/tnl:113501048) [K09580](https://www.genome.jp/entry/K09580) protein disulfide-isomerase

[tnl:113501793](https://www.genome.jp/entry/tnl:113501793) [K08056](https://www.genome.jp/entry/K08056) protein disulfide-isomerase A3

[tnl:113502117](https://www.genome.jp/entry/tnl:113502117) [K14009](https://www.genome.jp/entry/K14009) B-cell receptor-associated protein 31

[tnl:113502223](https://www.genome.jp/entry/tnl:113502223) [K14014](https://www.genome.jp/entry/K14014) small VCP/p97-interacting protein isoform X1

[tnl:113502294](https://www.genome.jp/entry/tnl:113502294) [K08057](https://www.genome.jp/entry/K08057) calreticulin

[tnl:113502317](https://www.genome.jp/entry/tnl:113502317) [K14018](https://www.genome.jp/entry/K14018) phospholipase A-2-activating protein

[tnl:113502801](https://www.genome.jp/entry/tnl:113502801) [K10575](https://www.genome.jp/entry/K10575) ubiquitin-conjugating enzyme E2 G1-like isoform X1

[tnl:113503585](https://www.genome.jp/entry/tnl:113503585) [K04523](https://www.genome.jp/entry/K04523) ubiquilin-1 isoform X1

[tnl:113503670](https://www.genome.jp/entry/tnl:113503670) [K04440](https://www.genome.jp/entry/K04440) stress-activated protein kinase JNK isoform X1

[tnl:113504322](https://www.genome.jp/entry/tnl:113504322) [K24348](https://www.genome.jp/entry/K24348) UBX domain-containing protein 4

[tnl:113504454](https://www.genome.jp/entry/tnl:113504454) [K12670](https://www.genome.jp/entry/K12670) dolichyl-diphosphooligosaccharide--protein glycosyltransferase 48 kDa subunit

[tnl:113504818](https://www.genome.jp/entry/tnl:113504818) [K14006](https://www.genome.jp/entry/K14006) protein transport protein Sec23A isoform X1

[tnl:113504836](https://www.genome.jp/entry/tnl:113504836) [K14000](https://www.genome.jp/entry/K14000) ribosome-binding protein 1-like isoform X1

[tnl:113504919](https://www.genome.jp/entry/tnl:113504919) [K14026](https://www.genome.jp/entry/K14026) protein sel-1 homolog 1 isoform X1

[tnl:113505023](https://www.genome.jp/entry/tnl:113505023) [K14004](https://www.genome.jp/entry/K14004) protein SEC13 homolog

[tnl:113505102](https://www.genome.jp/entry/tnl:113505102) [K05546](https://www.genome.jp/entry/K05546) neutral alpha-glucosidase AB-like isoform X1

[tnl:113505278](https://www.genome.jp/entry/tnl:113505278) [K10636](https://www.genome.jp/entry/K10636) E3 ubiquitin-protein ligase AMFR-like

[tnl:113505390](https://www.genome.jp/entry/tnl:113505390) [K13525](https://www.genome.jp/entry/K13525) transitional endoplasmic reticulum ATPase TER94

[tnl:113506423](https://www.genome.jp/entry/tnl:113506423) [K03283](https://www.genome.jp/entry/K03283) heat shock 70 kDa protein cognate 4

[tnl:113506541](https://www.genome.jp/entry/tnl:113506541) [K10575](https://www.genome.jp/entry/K10575) ubiquitin-conjugating enzyme E2 G1-like

[tnl:113506768](https://www.genome.jp/entry/tnl:113506768) [K05546](https://www.genome.jp/entry/K05546) LOW QUALITY PROTEIN: neutral alpha-glucosidase AB-like

[tnl:113507644](https://www.genome.jp/entry/tnl:113507644) [K10597](https://www.genome.jp/entry/K10597) ubiquitin conjugation factor E4 B

[tnl:113508021](https://www.genome.jp/entry/tnl:113508021) [K10839](https://www.genome.jp/entry/K10839) UV excision repair protein RAD23 homolog A

[tnl:113508064](https://www.genome.jp/entry/tnl:113508064) [K09486](https://www.genome.jp/entry/K09486) hypoxia up-regulated protein 1

[tnl:113508299](https://www.genome.jp/entry/tnl:113508299) [K03094](https://www.genome.jp/entry/K03094) S-phase kinase-associated protein 1

[tnl:113508548](https://www.genome.jp/entry/tnl:113508548) [K08054](https://www.genome.jp/entry/K08054) calnexin

 [**tnl01240**](https://www.genome.jp/kegg-bin/show_pathway?166859291342340/tnl01240.args) **Biosynthesis of cofactors - Trichoplusia ni (cabbage looper) (**[**40**](javascript:display('tnl01240'))**)**

[tnl:113491873](https://www.genome.jp/entry/tnl:113491873) [K00600](https://www.genome.jp/entry/K00600) serine hydroxymethyltransferase, cytosolic isoform X1

[tnl:113492544](https://www.genome.jp/entry/tnl:113492544) [K00940](https://www.genome.jp/entry/K00940) nucleoside diphosphate kinase

[tnl:113492551](https://www.genome.jp/entry/tnl:113492551) [K01432](https://www.genome.jp/entry/K01432) kynurenine formamidase isoform X1

[tnl:113492726](https://www.genome.jp/entry/tnl:113492726) [K11204](https://www.genome.jp/entry/K11204) glutamate--cysteine ligase catalytic subunit

[tnl:113492848](https://www.genome.jp/entry/tnl:113492848) [K11153](https://www.genome.jp/entry/K11153) retinol dehydrogenase 13-like

[tnl:113494165](https://www.genome.jp/entry/tnl:113494165) [K00966](https://www.genome.jp/entry/K00966) mannose-1-phosphate guanyltransferase alpha-A

[tnl:113494198](https://www.genome.jp/entry/tnl:113494198) [K11205](https://www.genome.jp/entry/K11205) glutamate--cysteine ligase regulatory subunit isoform X1

[tnl:113494305](https://www.genome.jp/entry/tnl:113494305) [K21456](https://www.genome.jp/entry/K21456) glutathione synthetase-like isoform X1

[tnl:113494620](https://www.genome.jp/entry/tnl:113494620) [K01698](https://www.genome.jp/entry/K01698) delta-aminolevulinic acid dehydratase

[tnl:113494797](https://www.genome.jp/entry/tnl:113494797) [K01756](https://www.genome.jp/entry/K01756) adenylosuccinate lyase-like

[tnl:113495149](https://www.genome.jp/entry/tnl:113495149) [K06210](https://www.genome.jp/entry/K06210) nicotinamide/nicotinic acid mononucleotide adenylyltransferase 1

[tnl:113496813](https://www.genome.jp/entry/tnl:113496813) [K00939](https://www.genome.jp/entry/K00939) adenylate kinase

[tnl:113497302](https://www.genome.jp/entry/tnl:113497302) [K00072](https://www.genome.jp/entry/K00072) sepiapterin reductase

[tnl:113498448](https://www.genome.jp/entry/tnl:113498448) [K14163](https://www.genome.jp/entry/K14163) bifunctional glutamate/proline--tRNA ligase isoform X1

[tnl:113498462](https://www.genome.jp/entry/tnl:113498462) [K00275](https://www.genome.jp/entry/K00275) pyridoxine-5'-phosphate oxidase-like

[tnl:113499034](https://www.genome.jp/entry/tnl:113499034) [K00868](https://www.genome.jp/entry/K00868) pyridoxal kinase

[tnl:113499353](https://www.genome.jp/entry/tnl:113499353) [K00861](https://www.genome.jp/entry/K00861) riboflavin kinase

[tnl:113500261](https://www.genome.jp/entry/tnl:113500261) [K01053](https://www.genome.jp/entry/K01053) regucalcin-like isoform X1

[tnl:113500574](https://www.genome.jp/entry/tnl:113500574) [K00831](https://www.genome.jp/entry/K00831) probable phosphoserine aminotransferase

[tnl:113500921](https://www.genome.jp/entry/tnl:113500921) [K00128](https://www.genome.jp/entry/K00128) retinal dehydrogenase 1-like

[tnl:113500928](https://www.genome.jp/entry/tnl:113500928) [K00789](https://www.genome.jp/entry/K00789) S-adenosylmethionine synthase isoform X1

[tnl:113501660](https://www.genome.jp/entry/tnl:113501660) [K00963](https://www.genome.jp/entry/K00963) UTP--glucose-1-phosphate uridylyltransferase isoform X1

[tnl:113501798](https://www.genome.jp/entry/tnl:113501798) [K18532](https://www.genome.jp/entry/K18532) adenylate kinase isoenzyme 6 homolog

[tnl:113502186](https://www.genome.jp/entry/tnl:113502186) [K00763](https://www.genome.jp/entry/K00763) nicotinate phosphoribosyltransferase isoform X1

[tnl:113502955](https://www.genome.jp/entry/tnl:113502955) [K15376](https://www.genome.jp/entry/K15376) gephyrin

[tnl:113503426](https://www.genome.jp/entry/tnl:113503426) [K13403](https://www.genome.jp/entry/K13403) bifunctional methylenetetrahydrofolate dehydrogenase/cyclohydrolase, mitochondrial isoform X1

[tnl:113503573](https://www.genome.jp/entry/tnl:113503573) [K00128](https://www.genome.jp/entry/K00128) aldehyde dehydrogenase, mitochondrial

[tnl:113503650](https://www.genome.jp/entry/tnl:113503650) [K01077](https://www.genome.jp/entry/K01077) alkaline phosphatase-like

[tnl:113504172](https://www.genome.jp/entry/tnl:113504172) [K00287](https://www.genome.jp/entry/K00287) dihydrofolate reductase isoform X1

[tnl:113504558](https://www.genome.jp/entry/tnl:113504558) [K11540](https://www.genome.jp/entry/K11540) CAD protein isoform X1

[tnl:113504856](https://www.genome.jp/entry/tnl:113504856) [K01939](https://www.genome.jp/entry/K01939) adenylosuccinate synthetase-like

[tnl:113505327](https://www.genome.jp/entry/tnl:113505327) [K13800](https://www.genome.jp/entry/K13800) UMP-CMP kinase

[tnl:113505855](https://www.genome.jp/entry/tnl:113505855) [K01809](https://www.genome.jp/entry/K01809) mannose-6-phosphate isomerase

[tnl:113506824](https://www.genome.jp/entry/tnl:113506824) [K15734](https://www.genome.jp/entry/K15734) short-chain dehydrogenase/reductase family 16C member 6-like

[tnl:113507725](https://www.genome.jp/entry/tnl:113507725) [K01937](https://www.genome.jp/entry/K01937) CTP synthase

[tnl:113507735](https://www.genome.jp/entry/tnl:113507735) [K15734](https://www.genome.jp/entry/K15734) short-chain dehydrogenase/reductase family 16C member 6-like isoform X1

[tnl:113507917](https://www.genome.jp/entry/tnl:113507917) [K00012](https://www.genome.jp/entry/K00012) UDP-glucose 6-dehydrogenase

[tnl:113507969](https://www.genome.jp/entry/tnl:113507969) [K01939](https://www.genome.jp/entry/K01939) LOW QUALITY PROTEIN: adenylosuccinate synthetase-like

[tnl:113508098](https://www.genome.jp/entry/tnl:113508098) [K17497](https://www.genome.jp/entry/K17497) phosphomannomutase 2

[tnl:113508348](https://www.genome.jp/entry/tnl:113508348) [K01599](https://www.genome.jp/entry/K01599) uroporphyrinogen decarboxylase

 [**tnl04150**](https://www.genome.jp/kegg-bin/show_pathway?166859291342340/tnl04150.args) **mTOR signaling pathway - Trichoplusia ni (cabbage looper) (**[**39**](javascript:display('tnl04150'))**)**

[tnl:113491757](https://www.genome.jp/entry/tnl:113491757) [K04371](https://www.genome.jp/entry/K04371) mitogen-activated protein kinase ERK-A

[tnl:113492015](https://www.genome.jp/entry/tnl:113492015) [K04364](https://www.genome.jp/entry/K04364) protein enhancer of sevenless 2B

[tnl:113492491](https://www.genome.jp/entry/tnl:113492491) [K06519](https://www.genome.jp/entry/K06519) uncharacterized protein LOC113492491 isoform X1

[tnl:113492751](https://www.genome.jp/entry/tnl:113492751) [K02144](https://www.genome.jp/entry/K02144) V-type proton ATPase subunit H isoform X1

[tnl:113493331](https://www.genome.jp/entry/tnl:113493331) [K13780](https://www.genome.jp/entry/K13780) large neutral amino acids transporter small subunit 1

[tnl:113493432](https://www.genome.jp/entry/tnl:113493432) [K03259](https://www.genome.jp/entry/K03259) eukaryotic translation initiation factor 4E type 2

[tnl:113493450](https://www.genome.jp/entry/tnl:113493450) [K08272](https://www.genome.jp/entry/K08272) protein Mo25

[tnl:113493668](https://www.genome.jp/entry/tnl:113493668) [K04365](https://www.genome.jp/entry/K04365) raf homolog serine/threonine-protein kinase Raf isoform X1

[tnl:113493805](https://www.genome.jp/entry/tnl:113493805) [K07827](https://www.genome.jp/entry/K07827) GTPase HRas

[tnl:113494528](https://www.genome.jp/entry/tnl:113494528) [K20410](https://www.genome.jp/entry/K20410) stress-activated map kinase-interacting protein 1

[tnl:113494681](https://www.genome.jp/entry/tnl:113494681) [K02147](https://www.genome.jp/entry/K02147) V-type proton ATPase subunit B

[tnl:113494981](https://www.genome.jp/entry/tnl:113494981) [K08267](https://www.genome.jp/entry/K08267) rapamycin-insensitive companion of mTOR

[tnl:113495052](https://www.genome.jp/entry/tnl:113495052) [K07208](https://www.genome.jp/entry/K07208) GTP-binding protein Rheb homolog

[tnl:113495656](https://www.genome.jp/entry/tnl:113495656) [K04456](https://www.genome.jp/entry/K04456) RAC serine/threonine-protein kinase

[tnl:113496116](https://www.genome.jp/entry/tnl:113496116) [K02145](https://www.genome.jp/entry/K02145) V-type proton ATPase catalytic subunit A

[tnl:113496232](https://www.genome.jp/entry/tnl:113496232) [K02149](https://www.genome.jp/entry/K02149) V-type proton ATPase subunit D

[tnl:113497664](https://www.genome.jp/entry/tnl:113497664) [K01110](https://www.genome.jp/entry/K01110) phosphatidylinositol 3,4,5-trisphosphate 3-phosphatase and dual-specificity protein phosphatase PTEN

[tnl:113497716](https://www.genome.jp/entry/tnl:113497716) [K08271](https://www.genome.jp/entry/K08271) STE20-related kinase adapter protein alpha isoform X1

[tnl:113497899](https://www.genome.jp/entry/tnl:113497899) [K04373](https://www.genome.jp/entry/K04373) ribosomal protein S6 kinase 2 beta isoform X1

[tnl:113498219](https://www.genome.jp/entry/tnl:113498219) [K02375](https://www.genome.jp/entry/K02375) frizzled-2 isoform X1

[tnl:113499397](https://www.genome.jp/entry/tnl:113499397) [K02150](https://www.genome.jp/entry/K02150) V-type proton ATPase subunit E

[tnl:113500508](https://www.genome.jp/entry/tnl:113500508) [K02151](https://www.genome.jp/entry/K02151) V-type proton ATPase subunit F

[tnl:113500972](https://www.genome.jp/entry/tnl:113500972) [K02432](https://www.genome.jp/entry/K02432) frizzled-7-B-like

[tnl:113502271](https://www.genome.jp/entry/tnl:113502271) [K03259](https://www.genome.jp/entry/K03259) eukaryotic translation initiation factor 4E-like

[tnl:113502394](https://www.genome.jp/entry/tnl:113502394) [K07298](https://www.genome.jp/entry/K07298) serine/threonine-protein kinase STK11 isoform X1

[tnl:113503039](https://www.genome.jp/entry/tnl:113503039) [K07198](https://www.genome.jp/entry/K07198) 5'-AMP-activated protein kinase catalytic subunit alpha-2 isoform X1

[tnl:113503154](https://www.genome.jp/entry/tnl:113503154) [K02148](https://www.genome.jp/entry/K02148) V-type proton ATPase subunit C

[tnl:113503306](https://www.genome.jp/entry/tnl:113503306) [K02991](https://www.genome.jp/entry/K02991) 40S ribosomal protein S6

[tnl:113503427](https://www.genome.jp/entry/tnl:113503427) [K04513](https://www.genome.jp/entry/K04513) ras-like GTP-binding protein Rho1 isoform X1

[tnl:113503523](https://www.genome.jp/entry/tnl:113503523) [K04368](https://www.genome.jp/entry/K04368) dual specificity mitogen-activated protein kinase kinase dSOR1

[tnl:113503740](https://www.genome.jp/entry/tnl:113503740) [K03258](https://www.genome.jp/entry/K03258) eukaryotic translation initiation factor 4B

[tnl:113503892](https://www.genome.jp/entry/tnl:113503892) [K02842](https://www.genome.jp/entry/K02842) frizzled-10-like

[tnl:113504422](https://www.genome.jp/entry/tnl:113504422) [K04688](https://www.genome.jp/entry/K04688) ribosomal protein S6 kinase beta-2

[tnl:113505023](https://www.genome.jp/entry/tnl:113505023) [K14004](https://www.genome.jp/entry/K14004) protein SEC13 homolog

[tnl:113506207](https://www.genome.jp/entry/tnl:113506207) [K02152](https://www.genome.jp/entry/K02152) V-type proton ATPase subunit G

[tnl:113507623](https://www.genome.jp/entry/tnl:113507623) [K07203](https://www.genome.jp/entry/K07203) serine/threonine-protein kinase Tor-like isoform X1

[tnl:113507914](https://www.genome.jp/entry/tnl:113507914) [K02353](https://www.genome.jp/entry/K02353) LOW QUALITY PROTEIN: segment polarity protein dishevelled homolog DVL-3-like

[tnl:113507920](https://www.genome.jp/entry/tnl:113507920) [K02353](https://www.genome.jp/entry/K02353) LOW QUALITY PROTEIN: segment polarity protein dishevelled homolog DVL-3-like

[tnl:113508080](https://www.genome.jp/entry/tnl:113508080) [K07207](https://www.genome.jp/entry/K07207) tuberin

**** [**tnl00230**](https://www.genome.jp/kegg-bin/show_pathway?166859291342340/tnl00230.args) **Purine metabolism - Trichoplusia ni (cabbage looper) (**[**37**](javascript:display('tnl00230'))**)**

[tnl:113491632](https://www.genome.jp/entry/tnl:113491632) [K01514](https://www.genome.jp/entry/K01514) exopolyphosphatase PRUNE1

[tnl:113492544](https://www.genome.jp/entry/tnl:113492544) [K00940](https://www.genome.jp/entry/K00940) nucleoside diphosphate kinase

[tnl:113493155](https://www.genome.jp/entry/tnl:113493155) [K13755](https://www.genome.jp/entry/K13755) calcium/calmodulin-dependent 3',5'-cyclic nucleotide phosphodiesterase 1 isoform X1

[tnl:113493828](https://www.genome.jp/entry/tnl:113493828) [K00948](https://www.genome.jp/entry/K00948) ribose-phosphate pyrophosphokinase 2

[tnl:113494314](https://www.genome.jp/entry/tnl:113494314) [K00364](https://www.genome.jp/entry/K00364) GMP reductase 1-like

[tnl:113494579](https://www.genome.jp/entry/tnl:113494579) [K00764](https://www.genome.jp/entry/K00764) amidophosphoribosyltransferase-like isoform X1

[tnl:113494584](https://www.genome.jp/entry/tnl:113494584) [K01587](https://www.genome.jp/entry/K01587) multifunctional protein ADE2

[tnl:113494755](https://www.genome.jp/entry/tnl:113494755) [K12304](https://www.genome.jp/entry/K12304) soluble calcium-activated nucleotidase 1

[tnl:113494797](https://www.genome.jp/entry/tnl:113494797) [K01756](https://www.genome.jp/entry/K01756) adenylosuccinate lyase-like

[tnl:113496055](https://www.genome.jp/entry/tnl:113496055) [K03783](https://www.genome.jp/entry/K03783) purine nucleoside phosphorylase-like isoform X1

[tnl:113496347](https://www.genome.jp/entry/tnl:113496347) [K10808](https://www.genome.jp/entry/K10808) ribonucleoside-diphosphate reductase subunit M2

[tnl:113496570](https://www.genome.jp/entry/tnl:113496570) [K00942](https://www.genome.jp/entry/K00942) guanylate kinase isoform X1

[tnl:113496813](https://www.genome.jp/entry/tnl:113496813) [K00939](https://www.genome.jp/entry/K00939) adenylate kinase

[tnl:113497121](https://www.genome.jp/entry/tnl:113497121) [K10807](https://www.genome.jp/entry/K10807) ribonucleoside-diphosphate reductase large subunit

[tnl:113497732](https://www.genome.jp/entry/tnl:113497732) [K00106](https://www.genome.jp/entry/K00106) xanthine dehydrogenase

[tnl:113497953](https://www.genome.jp/entry/tnl:113497953) [K01081](https://www.genome.jp/entry/K01081) cytosolic purine 5'-nucleotidase isoform X1

[tnl:113498801](https://www.genome.jp/entry/tnl:113498801) [K13988](https://www.genome.jp/entry/K13988) ADP-ribose pyrophosphatase, mitochondrial

[tnl:113499175](https://www.genome.jp/entry/tnl:113499175) [K01487](https://www.genome.jp/entry/K01487) guanine deaminase-like

[tnl:113499305](https://www.genome.jp/entry/tnl:113499305) [K01487](https://www.genome.jp/entry/K01487) LOW QUALITY PROTEIN: guanine deaminase-like

[tnl:113499351](https://www.genome.jp/entry/tnl:113499351) [K01951](https://www.genome.jp/entry/K01951) GMP synthase [glutamine-hydrolyzing] isoform X1

[tnl:113500912](https://www.genome.jp/entry/tnl:113500912) [K00602](https://www.genome.jp/entry/K00602) bifunctional purine biosynthesis protein PURH-like

[tnl:113501491](https://www.genome.jp/entry/tnl:113501491) [K01519](https://www.genome.jp/entry/K01519) inosine triphosphate pyrophosphatase

[tnl:113501500](https://www.genome.jp/entry/tnl:113501500) [K08041](https://www.genome.jp/entry/K08041) Ca(2+)/calmodulin-responsive adenylate cyclase-like

[tnl:113501642](https://www.genome.jp/entry/tnl:113501642) [K07023](https://www.genome.jp/entry/K07023) HD domain-containing protein 2

[tnl:113501798](https://www.genome.jp/entry/tnl:113501798) [K18532](https://www.genome.jp/entry/K18532) adenylate kinase isoenzyme 6 homolog

[tnl:113502400](https://www.genome.jp/entry/tnl:113502400) [K13811](https://www.genome.jp/entry/K13811) bifunctional 3'-phosphoadenosine 5'-phosphosulfate synthase isoform X1

[tnl:113502690](https://www.genome.jp/entry/tnl:113502690) [K01952](https://www.genome.jp/entry/K01952) phosphoribosylformylglycinamidine synthase-like

[tnl:113503659](https://www.genome.jp/entry/tnl:113503659) [K01835](https://www.genome.jp/entry/K01835) phosphoglucomutase

[tnl:113503681](https://www.genome.jp/entry/tnl:113503681) [K00856](https://www.genome.jp/entry/K00856) adenosine kinase isoform X1

[tnl:113504622](https://www.genome.jp/entry/tnl:113504622) [K01769](https://www.genome.jp/entry/K01769) receptor-type guanylate cyclase Gyc76C-like isoform X1

[tnl:113504794](https://www.genome.jp/entry/tnl:113504794) [K01490](https://www.genome.jp/entry/K01490) AMP deaminase 2-like isoform X1

[tnl:113504856](https://www.genome.jp/entry/tnl:113504856) [K01939](https://www.genome.jp/entry/K01939) adenylosuccinate synthetase-like

[tnl:113505248](https://www.genome.jp/entry/tnl:113505248) [K01518](https://www.genome.jp/entry/K01518) bis(5'-nucleosyl)-tetraphosphatase [asymmetrical]

[tnl:113506886](https://www.genome.jp/entry/tnl:113506886) [K01952](https://www.genome.jp/entry/K01952) LOW QUALITY PROTEIN: phosphoribosylformylglycinamidine synthase-like

[tnl:113507969](https://www.genome.jp/entry/tnl:113507969) [K01939](https://www.genome.jp/entry/K01939) LOW QUALITY PROTEIN: adenylosuccinate synthetase-like

[tnl:113507970](https://www.genome.jp/entry/tnl:113507970) [K08049](https://www.genome.jp/entry/K08049) adenylate cyclase type 9

[tnl:113508532](https://www.genome.jp/entry/tnl:113508532) [K00088](https://www.genome.jp/entry/K00088) inosine-5'-monophosphate dehydrogenase

 [**tnl04013**](https://www.genome.jp/kegg-bin/show_pathway?166859291342340/tnl04013.args) **MAPK signaling pathway - fly - Trichoplusia ni (cabbage looper) (**[**35**](javascript:display('tnl04013'))**)**

[tnl:113491757](https://www.genome.jp/entry/tnl:113491757) [K04371](https://www.genome.jp/entry/K04371) mitogen-activated protein kinase ERK-A

[tnl:113492015](https://www.genome.jp/entry/tnl:113492015) [K04364](https://www.genome.jp/entry/K04364) protein enhancer of sevenless 2B

[tnl:113492044](https://www.genome.jp/entry/tnl:113492044) [K04506](https://www.genome.jp/entry/K04506) LOW QUALITY PROTEIN: E3 ubiquitin-protein ligase siah-1-like

[tnl:113492467](https://www.genome.jp/entry/tnl:113492467) [K04461](https://www.genome.jp/entry/K04461) protein phosphatase 1A

[tnl:113492667](https://www.genome.jp/entry/tnl:113492667) [K08840](https://www.genome.jp/entry/K08840) serine/threonine-protein kinase mig-15 isoform X1

[tnl:113492716](https://www.genome.jp/entry/tnl:113492716) [K04392](https://www.genome.jp/entry/K04392) ras-like GTP-binding protein RhoL

[tnl:113492784](https://www.genome.jp/entry/tnl:113492784) [K12380](https://www.genome.jp/entry/K12380) GTPase-activating protein

[tnl:113493455](https://www.genome.jp/entry/tnl:113493455) [K05759](https://www.genome.jp/entry/K05759) profilin

[tnl:113493668](https://www.genome.jp/entry/tnl:113493668) [K04365](https://www.genome.jp/entry/K04365) raf homolog serine/threonine-protein kinase Raf isoform X1

[tnl:113493805](https://www.genome.jp/entry/tnl:113493805) [K07827](https://www.genome.jp/entry/K07827) GTPase HRas

[tnl:113494031](https://www.genome.jp/entry/tnl:113494031) [K20231](https://www.genome.jp/entry/K20231) uncharacterized protein LOC113494031 isoform X1

[tnl:113494342](https://www.genome.jp/entry/tnl:113494342) [K04506](https://www.genome.jp/entry/K04506) E3 ubiquitin-protein ligase sina-like isoform X1

[tnl:113494585](https://www.genome.jp/entry/tnl:113494585) [K04431](https://www.genome.jp/entry/K04431) dual specificity mitogen-activated protein kinase kinase 7-like isoform X1

[tnl:113494689](https://www.genome.jp/entry/tnl:113494689) [K04392](https://www.genome.jp/entry/K04392) ras-related protein Rac1

[tnl:113496323](https://www.genome.jp/entry/tnl:113496323) [K16197](https://www.genome.jp/entry/K16197) 14-3-3 protein zeta isoform X1

[tnl:113496933](https://www.genome.jp/entry/tnl:113496933) [K06279](https://www.genome.jp/entry/K06279) SHC-transforming protein 1

[tnl:113497250](https://www.genome.jp/entry/tnl:113497250) [K17608](https://www.genome.jp/entry/K17608) striatin-3

[tnl:113497265](https://www.genome.jp/entry/tnl:113497265) [K05704](https://www.genome.jp/entry/K05704) tyrosine-protein kinase Src64B isoform X1

[tnl:113497314](https://www.genome.jp/entry/tnl:113497314) [K04404](https://www.genome.jp/entry/K04404) uncharacterized protein LOC113497314 isoform X1

[tnl:113497556](https://www.genome.jp/entry/tnl:113497556) [K20230](https://www.genome.jp/entry/K20230) immunoglobulin superfamily containing leucine-rich repeat protein

[tnl:113497745](https://www.genome.jp/entry/tnl:113497745) [K06689](https://www.genome.jp/entry/K06689) ubiquitin-conjugating enzyme E2-17 kDa

[tnl:113497767](https://www.genome.jp/entry/tnl:113497767) [K14290](https://www.genome.jp/entry/K14290) exportin-1

[tnl:113499516](https://www.genome.jp/entry/tnl:113499516) [K04382](https://www.genome.jp/entry/K04382) serine/threonine-protein phosphatase 2A catalytic subunit beta isoform

[tnl:113501693](https://www.genome.jp/entry/tnl:113501693) [K17512](https://www.genome.jp/entry/K17512) tyrosine-protein kinase Shark isoform X1

[tnl:113501750](https://www.genome.jp/entry/tnl:113501750) [K18529](https://www.genome.jp/entry/K18529) kinase suppressor of Ras 2 isoform X1

[tnl:113501942](https://www.genome.jp/entry/tnl:113501942) [K04441](https://www.genome.jp/entry/K04441) mitogen-activated protein kinase p38b-like isoform X1

[tnl:113503316](https://www.genome.jp/entry/tnl:113503316) [K04352](https://www.genome.jp/entry/K04352) ras GTPase-activating protein 1

[tnl:113503523](https://www.genome.jp/entry/tnl:113503523) [K04368](https://www.genome.jp/entry/K04368) dual specificity mitogen-activated protein kinase kinase dSOR1

[tnl:113503612](https://www.genome.jp/entry/tnl:113503612) [K02104](https://www.genome.jp/entry/K02104) protein giant-lens

[tnl:113503669](https://www.genome.jp/entry/tnl:113503669) [K13578](https://www.genome.jp/entry/K13578) bone morphogenetic protein receptor type-1B isoform X1

[tnl:113503670](https://www.genome.jp/entry/tnl:113503670) [K04440](https://www.genome.jp/entry/K04440) stress-activated protein kinase JNK isoform X1

[tnl:113505770](https://www.genome.jp/entry/tnl:113505770) [K20234](https://www.genome.jp/entry/K20234) fibroblast growth factor receptor substrate 2

[tnl:113505956](https://www.genome.jp/entry/tnl:113505956) [K04392](https://www.genome.jp/entry/K04392) ras-related C3 botulinum toxin substrate 1

[tnl:113507706](https://www.genome.jp/entry/tnl:113507706) [K17383](https://www.genome.jp/entry/K17383) protein sprouty

[tnl:113508374](https://www.genome.jp/entry/tnl:113508374) [K20223](https://www.genome.jp/entry/K20223) importin-7 isoform X1

 [**tnl01200**](https://www.genome.jp/kegg-bin/show_pathway?166859291342340/tnl01200.args) **Carbon metabolism - Trichoplusia ni (cabbage looper) (**[**33**](javascript:display('tnl01200'))**)**

[tnl:113491873](https://www.genome.jp/entry/tnl:113491873) [K00600](https://www.genome.jp/entry/K00600) serine hydroxymethyltransferase, cytosolic isoform X1

[tnl:113492358](https://www.genome.jp/entry/tnl:113492358) [K00658](https://www.genome.jp/entry/K00658) dihydrolipoyllysine-residue succinyltransferase component of 2-oxoglutarate dehydrogenase complex, mitochondrial-like

[tnl:113492630](https://www.genome.jp/entry/tnl:113492630) [K00029](https://www.genome.jp/entry/K00029) LOW QUALITY PROTEIN: NADP-dependent malic enzyme-like

[tnl:113492641](https://www.genome.jp/entry/tnl:113492641) [K01070](https://www.genome.jp/entry/K01070) S-formylglutathione hydrolase isoform X1

[tnl:113492710](https://www.genome.jp/entry/tnl:113492710) [K00031](https://www.genome.jp/entry/K00031) isocitrate dehydrogenase [NADP] cytoplasmic-like

[tnl:113492937](https://www.genome.jp/entry/tnl:113492937) [K14454](https://www.genome.jp/entry/K14454) aspartate aminotransferase, cytoplasmic

[tnl:113493828](https://www.genome.jp/entry/tnl:113493828) [K00948](https://www.genome.jp/entry/K00948) ribose-phosphate pyrophosphokinase 2

[tnl:113493924](https://www.genome.jp/entry/tnl:113493924) [K01803](https://www.genome.jp/entry/K01803) triosephosphate isomerase

[tnl:113494316](https://www.genome.jp/entry/tnl:113494316) [K01810](https://www.genome.jp/entry/K01810) glucose-6-phosphate isomerase

[tnl:113494495](https://www.genome.jp/entry/tnl:113494495) [K00927](https://www.genome.jp/entry/K00927) phosphoglycerate kinase

[tnl:113495603](https://www.genome.jp/entry/tnl:113495603) [K01679](https://www.genome.jp/entry/K01679) fumarate hydratase, mitochondrial-like isoform X1

[tnl:113495760](https://www.genome.jp/entry/tnl:113495760) [K03781](https://www.genome.jp/entry/K03781) catalase

[tnl:113496177](https://www.genome.jp/entry/tnl:113496177) [K00058](https://www.genome.jp/entry/K00058) D-3-phosphoglycerate dehydrogenase

[tnl:113496310](https://www.genome.jp/entry/tnl:113496310) [K00850](https://www.genome.jp/entry/K00850) ATP-dependent 6-phosphofructokinase isoform X1

[tnl:113496832](https://www.genome.jp/entry/tnl:113496832) [K00261](https://www.genome.jp/entry/K00261) glutamate dehydrogenase, mitochondrial

[tnl:113497832](https://www.genome.jp/entry/tnl:113497832) [K00025](https://www.genome.jp/entry/K00025) malate dehydrogenase, cytoplasmic

[tnl:113497863](https://www.genome.jp/entry/tnl:113497863) [K00121](https://www.genome.jp/entry/K00121) alcohol dehydrogenase class-3

[tnl:113497947](https://www.genome.jp/entry/tnl:113497947) [K00844](https://www.genome.jp/entry/K00844) hexokinase type 2 isoform X1

[tnl:113498020](https://www.genome.jp/entry/tnl:113498020) [K00626](https://www.genome.jp/entry/K00626) acetyl-CoA acetyltransferase, mitochondrial

[tnl:113498369](https://www.genome.jp/entry/tnl:113498369) [K00814](https://www.genome.jp/entry/K00814) alanine aminotransferase 1-like isoform X1

[tnl:113499004](https://www.genome.jp/entry/tnl:113499004) [K01689](https://www.genome.jp/entry/K01689) enolase

[tnl:113499721](https://www.genome.jp/entry/tnl:113499721) [K00615](https://www.genome.jp/entry/K00615) transketolase-like protein 2

[tnl:113500261](https://www.genome.jp/entry/tnl:113500261) [K01053](https://www.genome.jp/entry/K01053) regucalcin-like isoform X1

[tnl:113500423](https://www.genome.jp/entry/tnl:113500423) [K00873](https://www.genome.jp/entry/K00873) pyruvate kinase-like isoform X1

[tnl:113500574](https://www.genome.jp/entry/tnl:113500574) [K00831](https://www.genome.jp/entry/K00831) probable phosphoserine aminotransferase

[tnl:113501060](https://www.genome.jp/entry/tnl:113501060) [K00036](https://www.genome.jp/entry/K00036) glucose-6-phosphate 1-dehydrogenase

[tnl:113501275](https://www.genome.jp/entry/tnl:113501275) [K19269](https://www.genome.jp/entry/K19269) glycerol-3-phosphate phosphatase-like

[tnl:113503107](https://www.genome.jp/entry/tnl:113503107) [K01057](https://www.genome.jp/entry/K01057) 6-phosphogluconolactonase

[tnl:113504438](https://www.genome.jp/entry/tnl:113504438) [K00031](https://www.genome.jp/entry/K00031) isocitrate dehydrogenase [NADP] cytoplasmic isoform X1

[tnl:113506529](https://www.genome.jp/entry/tnl:113506529) [K01834](https://www.genome.jp/entry/K01834) phosphoglycerate mutase 2-like

[tnl:113508324](https://www.genome.jp/entry/tnl:113508324) [K00616](https://www.genome.jp/entry/K00616) probable transaldolase

[tnl:113508342](https://www.genome.jp/entry/tnl:113508342) [K01807](https://www.genome.jp/entry/K01807) ribose-5-phosphate isomerase

[tnl:113509001](https://www.genome.jp/entry/tnl:113509001) [K00134](https://www.genome.jp/entry/K00134) glyceraldehyde-3-phosphate dehydrogenase

 [**tnl03050**](https://www.genome.jp/kegg-bin/show_pathway?166859291342340/tnl03050.args) **Proteasome - Trichoplusia ni (cabbage looper) (**[**33**](javascript:display('tnl03050'))**)**

[tnl:113491986](https://www.genome.jp/entry/tnl:113491986) [K03035](https://www.genome.jp/entry/K03035) 26S proteasome non-ATPase regulatory subunit 12

[tnl:113492132](https://www.genome.jp/entry/tnl:113492132) [K03029](https://www.genome.jp/entry/K03029) 26S proteasome non-ATPase regulatory subunit 4

[tnl:113492787](https://www.genome.jp/entry/tnl:113492787) [K03030](https://www.genome.jp/entry/K03030) 26S proteasome non-ATPase regulatory subunit 14

[tnl:113492907](https://www.genome.jp/entry/tnl:113492907) [K02738](https://www.genome.jp/entry/K02738) proteasome subunit beta type-6

[tnl:113493235](https://www.genome.jp/entry/tnl:113493235) [K02731](https://www.genome.jp/entry/K02731) proteasome subunit alpha type-7-1

[tnl:113493286](https://www.genome.jp/entry/tnl:113493286) [K02739](https://www.genome.jp/entry/K02739) proteasome subunit beta type-7-like

[tnl:113493512](https://www.genome.jp/entry/tnl:113493512) [K03033](https://www.genome.jp/entry/K03033) probable 26S proteasome non-ATPase regulatory subunit 3

[tnl:113493521](https://www.genome.jp/entry/tnl:113493521) [K03037](https://www.genome.jp/entry/K03037) 26S proteasome non-ATPase regulatory subunit 6

[tnl:113493840](https://www.genome.jp/entry/tnl:113493840) [K02735](https://www.genome.jp/entry/K02735) proteasome subunit beta type-3

[tnl:113494135](https://www.genome.jp/entry/tnl:113494135) [K06693](https://www.genome.jp/entry/K06693) 26S proteasome non-ATPase regulatory subunit 9

[tnl:113494173](https://www.genome.jp/entry/tnl:113494173) [K02737](https://www.genome.jp/entry/K02737) proteasome subunit beta type-5

[tnl:113494351](https://www.genome.jp/entry/tnl:113494351) [K03066](https://www.genome.jp/entry/K03066) 26S proteasome regulatory subunit 8

[tnl:113494826](https://www.genome.jp/entry/tnl:113494826) [K03064](https://www.genome.jp/entry/K03064) 26S proteasome regulatory subunit 10B

[tnl:113495567](https://www.genome.jp/entry/tnl:113495567) [K03031](https://www.genome.jp/entry/K03031) 26S proteasome non-ATPase regulatory subunit 8

[tnl:113495599](https://www.genome.jp/entry/tnl:113495599) [K02727](https://www.genome.jp/entry/K02727) proteasome subunit alpha type-3

[tnl:113495660](https://www.genome.jp/entry/tnl:113495660) [K02734](https://www.genome.jp/entry/K02734) proteasome subunit beta type-2

[tnl:113496212](https://www.genome.jp/entry/tnl:113496212) [K03063](https://www.genome.jp/entry/K03063) 26S proteasome regulatory subunit 6B

[tnl:113498313](https://www.genome.jp/entry/tnl:113498313) [K02730](https://www.genome.jp/entry/K02730) proteasome subunit alpha type-6-like

[tnl:113498463](https://www.genome.jp/entry/tnl:113498463) [K06698](https://www.genome.jp/entry/K06698) proteasome activator complex subunit 3 isoform X1

[tnl:113499000](https://www.genome.jp/entry/tnl:113499000) [K03028](https://www.genome.jp/entry/K03028) 26S proteasome non-ATPase regulatory subunit 2

[tnl:113499054](https://www.genome.jp/entry/tnl:113499054) [K03036](https://www.genome.jp/entry/K03036) 26S proteasome non-ATPase regulatory subunit 11

[tnl:113499378](https://www.genome.jp/entry/tnl:113499378) [K03061](https://www.genome.jp/entry/K03061) 26S proteasome regulatory subunit 7

[tnl:113499985](https://www.genome.jp/entry/tnl:113499985) [K02725](https://www.genome.jp/entry/K02725) proteasome subunit alpha type-1

[tnl:113501489](https://www.genome.jp/entry/tnl:113501489) [K03038](https://www.genome.jp/entry/K03038) 26S proteasome non-ATPase regulatory subunit 7

[tnl:113501753](https://www.genome.jp/entry/tnl:113501753) [K06691](https://www.genome.jp/entry/K06691) proteasomal ubiquitin receptor ADRM1 isoform X1

[tnl:113501817](https://www.genome.jp/entry/tnl:113501817) [K06700](https://www.genome.jp/entry/K06700) proteasome inhibitor PI31 subunit

[tnl:113504182](https://www.genome.jp/entry/tnl:113504182) [K02736](https://www.genome.jp/entry/K02736) proteasome subunit beta type-4

[tnl:113504569](https://www.genome.jp/entry/tnl:113504569) [K02728](https://www.genome.jp/entry/K02728) proteasome subunit alpha type-4

[tnl:113504711](https://www.genome.jp/entry/tnl:113504711) [K03065](https://www.genome.jp/entry/K03065) 26S proteasome regulatory subunit 6A-B

[tnl:113505281](https://www.genome.jp/entry/tnl:113505281) [K02732](https://www.genome.jp/entry/K02732) proteasome subunit beta type-1

[tnl:113505590](https://www.genome.jp/entry/tnl:113505590) [K02726](https://www.genome.jp/entry/K02726) proteasome subunit alpha type-2

[tnl:113506702](https://www.genome.jp/entry/tnl:113506702) [K03062](https://www.genome.jp/entry/K03062) LOW QUALITY PROTEIN: 26S proteasome regulatory subunit 4-like

[tnl:113506737](https://www.genome.jp/entry/tnl:113506737) [K03062](https://www.genome.jp/entry/K03062) 26S proteasome regulatory subunit 4

 [**tnl04142**](https://www.genome.jp/kegg-bin/show_pathway?166859291342340/tnl04142.args) **Lysosome - Trichoplusia ni (cabbage looper) (**[**33**](javascript:display('tnl04142'))**)**

[tnl:113491799](https://www.genome.jp/entry/tnl:113491799) [K12393](https://www.genome.jp/entry/K12393) AP-1 complex subunit mu-1

[tnl:113492649](https://www.genome.jp/entry/tnl:113492649) [K19363](https://www.genome.jp/entry/K19363) lipopolysaccharide-induced tumor necrosis factor-alpha factor homolog

[tnl:113492751](https://www.genome.jp/entry/tnl:113492751) [K02144](https://www.genome.jp/entry/K02144) V-type proton ATPase subunit H isoform X1

[tnl:113492846](https://www.genome.jp/entry/tnl:113492846) [K14410](https://www.genome.jp/entry/K14410) prostatic acid phosphatase-like isoform X1

[tnl:113493473](https://www.genome.jp/entry/tnl:113493473) [K02146](https://www.genome.jp/entry/K02146) V-type proton ATPase subunit d

[tnl:113493523](https://www.genome.jp/entry/tnl:113493523) [K12396](https://www.genome.jp/entry/K12396) AP-3 complex subunit delta

[tnl:113493578](https://www.genome.jp/entry/tnl:113493578) [K12392](https://www.genome.jp/entry/K12392) AP-1 complex subunit beta-1

[tnl:113493842](https://www.genome.jp/entry/tnl:113493842) [K04646](https://www.genome.jp/entry/K04646) clathrin heavy chain isoform X1

[tnl:113494320](https://www.genome.jp/entry/tnl:113494320) [K10532](https://www.genome.jp/entry/K10532) heparan-alpha-glucosaminide N-acetyltransferase-like

[tnl:113495786](https://www.genome.jp/entry/tnl:113495786) [K06497](https://www.genome.jp/entry/K06497) 23 kDa integral membrane protein-like

[tnl:113496175](https://www.genome.jp/entry/tnl:113496175) [K14410](https://www.genome.jp/entry/K14410) prostatic acid phosphatase isoform X1

[tnl:113497190](https://www.genome.jp/entry/tnl:113497190) [K12385](https://www.genome.jp/entry/K12385) NPC intracellular cholesterol transporter 1 isoform X1

[tnl:113497289](https://www.genome.jp/entry/tnl:113497289) [K12373](https://www.genome.jp/entry/K12373) chitooligosaccharidolytic beta-N-acetylglucosaminidase

[tnl:113497839](https://www.genome.jp/entry/tnl:113497839) [K12316](https://www.genome.jp/entry/K12316) lysosomal alpha-glucosidase-like

[tnl:113498588](https://www.genome.jp/entry/tnl:113498588) [K12387](https://www.genome.jp/entry/K12387) lysosomal-associated transmembrane protein 4A isoform X1

[tnl:113498601](https://www.genome.jp/entry/tnl:113498601) [K12373](https://www.genome.jp/entry/K12373) beta-hexosaminidase subunit beta-like isoform X1

[tnl:113498816](https://www.genome.jp/entry/tnl:113498816) [K12301](https://www.genome.jp/entry/K12301) putative inorganic phosphate cotransporter isoform X1

[tnl:113500022](https://www.genome.jp/entry/tnl:113500022) [K01374](https://www.genome.jp/entry/K01374) cathepsin O-like

[tnl:113500148](https://www.genome.jp/entry/tnl:113500148) [K01374](https://www.genome.jp/entry/K01374) cathepsin O-like

[tnl:113500605](https://www.genome.jp/entry/tnl:113500605) [K02154](https://www.genome.jp/entry/K02154) V-type proton ATPase 116 kDa subunit a

[tnl:113500873](https://www.genome.jp/entry/tnl:113500873) [K12385](https://www.genome.jp/entry/K12385) protein patched homolog 1-like

[tnl:113501292](https://www.genome.jp/entry/tnl:113501292) [K06528](https://www.genome.jp/entry/K06528) lysosome-associated membrane glycoprotein 1-like isoform X1

[tnl:113501700](https://www.genome.jp/entry/tnl:113501700) [K12404](https://www.genome.jp/entry/K12404) ADP-ribosylation factor-binding protein GGA2 isoform X1

[tnl:113502256](https://www.genome.jp/entry/tnl:113502256) [K12386](https://www.genome.jp/entry/K12386) cystinosin homolog isoform X1

[tnl:113502333](https://www.genome.jp/entry/tnl:113502333) [K03661](https://www.genome.jp/entry/K03661) V-type proton ATPase 21 kDa proteolipid subunit

[tnl:113503468](https://www.genome.jp/entry/tnl:113503468) [K24155](https://www.genome.jp/entry/K24155) dmX-like protein 2 isoform X1

[tnl:113504478](https://www.genome.jp/entry/tnl:113504478) [K06497](https://www.genome.jp/entry/K06497) CD63 antigen-like

[tnl:113505484](https://www.genome.jp/entry/tnl:113505484) [K03662](https://www.genome.jp/entry/K03662) V-type proton ATPase subunit S1

[tnl:113505850](https://www.genome.jp/entry/tnl:113505850) [K06546](https://www.genome.jp/entry/K06546) sialomucin core protein 24-like

[tnl:113506377](https://www.genome.jp/entry/tnl:113506377) [K01365](https://www.genome.jp/entry/K01365) cathepsin L-like

[tnl:113506669](https://www.genome.jp/entry/tnl:113506669) [K06497](https://www.genome.jp/entry/K06497) CD63 antigen-like

[tnl:113506671](https://www.genome.jp/entry/tnl:113506671) [K06497](https://www.genome.jp/entry/K06497) CD63 antigen-like isoform X1

[tnl:113506679](https://www.genome.jp/entry/tnl:113506679) [K12391](https://www.genome.jp/entry/K12391) AP-1 complex subunit gamma-1 isoform X1

 [**tnl04140**](https://www.genome.jp/kegg-bin/show_pathway?166859291342340/tnl04140.args) **Autophagy - animal - Trichoplusia ni (cabbage looper) (**[**31**](javascript:display('tnl04140'))**)**

[tnl:113491757](https://www.genome.jp/entry/tnl:113491757) [K04371](https://www.genome.jp/entry/K04371) mitogen-activated protein kinase ERK-A

[tnl:113492520](https://www.genome.jp/entry/tnl:113492520) [K08339](https://www.genome.jp/entry/K08339) autophagy protein 5

[tnl:113492607](https://www.genome.jp/entry/tnl:113492607) [K07897](https://www.genome.jp/entry/K07897) ras-related protein Rab-7a

[tnl:113493077](https://www.genome.jp/entry/tnl:113493077) [K08343](https://www.genome.jp/entry/K08343) ubiquitin-like-conjugating enzyme ATG3

[tnl:113493805](https://www.genome.jp/entry/tnl:113493805) [K07827](https://www.genome.jp/entry/K07827) GTPase HRas

[tnl:113494236](https://www.genome.jp/entry/tnl:113494236) [K03237](https://www.genome.jp/entry/K03237) eukaryotic translation initiation factor 2 subunit 1

[tnl:113495052](https://www.genome.jp/entry/tnl:113495052) [K07208](https://www.genome.jp/entry/K07208) GTP-binding protein Rheb homolog

[tnl:113495656](https://www.genome.jp/entry/tnl:113495656) [K04456](https://www.genome.jp/entry/K04456) RAC serine/threonine-protein kinase

[tnl:113495885](https://www.genome.jp/entry/tnl:113495885) [K08341](https://www.genome.jp/entry/K08341) gamma-aminobutyric acid receptor-associated protein

[tnl:113495905](https://www.genome.jp/entry/tnl:113495905) [K17907](https://www.genome.jp/entry/K17907) autophagy-related protein 9A

[tnl:113496361](https://www.genome.jp/entry/tnl:113496361) [K14381](https://www.genome.jp/entry/K14381) sequestosome-1-like isoform X1

[tnl:113496598](https://www.genome.jp/entry/tnl:113496598) [K17606](https://www.genome.jp/entry/K17606) immunoglobulin-binding protein 1

[tnl:113497664](https://www.genome.jp/entry/tnl:113497664) [K01110](https://www.genome.jp/entry/K01110) phosphatidylinositol 3,4,5-trisphosphate 3-phosphatase and dual-specificity protein phosphatase PTEN

[tnl:113498457](https://www.genome.jp/entry/tnl:113498457) [K07874](https://www.genome.jp/entry/K07874) ras-related protein ORAB-1

[tnl:113499516](https://www.genome.jp/entry/tnl:113499516) [K04382](https://www.genome.jp/entry/K04382) serine/threonine-protein phosphatase 2A catalytic subunit beta isoform

[tnl:113500058](https://www.genome.jp/entry/tnl:113500058) [K07925](https://www.genome.jp/entry/K07925) ras-related protein Rab-39B

[tnl:113500068](https://www.genome.jp/entry/tnl:113500068) [K07920](https://www.genome.jp/entry/K07920) putative Ras-related protein Rab-33

[tnl:113501292](https://www.genome.jp/entry/tnl:113501292) [K06528](https://www.genome.jp/entry/K06528) lysosome-associated membrane glycoprotein 1-like isoform X1

[tnl:113502222](https://www.genome.jp/entry/tnl:113502222) [K07830](https://www.genome.jp/entry/K07830) ras-like protein 2

[tnl:113502394](https://www.genome.jp/entry/tnl:113502394) [K07298](https://www.genome.jp/entry/K07298) serine/threonine-protein kinase STK11 isoform X1

[tnl:113503039](https://www.genome.jp/entry/tnl:113503039) [K07198](https://www.genome.jp/entry/K07198) 5'-AMP-activated protein kinase catalytic subunit alpha-2 isoform X1

[tnl:113503064](https://www.genome.jp/entry/tnl:113503064) [K08509](https://www.genome.jp/entry/K08509) synaptosomal-associated protein 29

[tnl:113503523](https://www.genome.jp/entry/tnl:113503523) [K04368](https://www.genome.jp/entry/K04368) dual specificity mitogen-activated protein kinase kinase dSOR1

[tnl:113503670](https://www.genome.jp/entry/tnl:113503670) [K04440](https://www.genome.jp/entry/K04440) stress-activated protein kinase JNK isoform X1

[tnl:113504422](https://www.genome.jp/entry/tnl:113504422) [K04688](https://www.genome.jp/entry/K04688) ribosomal protein S6 kinase beta-2

[tnl:113505027](https://www.genome.jp/entry/tnl:113505027) [K07901](https://www.genome.jp/entry/K07901) ras-related protein Rab-8A isoform X1

[tnl:113506377](https://www.genome.jp/entry/tnl:113506377) [K01365](https://www.genome.jp/entry/K01365) cathepsin L-like

[tnl:113506778](https://www.genome.jp/entry/tnl:113506778) [K08491](https://www.genome.jp/entry/K08491) uncharacterized protein LOC113506778 isoform X1

[tnl:113506804](https://www.genome.jp/entry/tnl:113506804) [K04345](https://www.genome.jp/entry/K04345) cAMP-dependent protein kinase catalytic subunit

[tnl:113507623](https://www.genome.jp/entry/tnl:113507623) [K07203](https://www.genome.jp/entry/K07203) serine/threonine-protein kinase Tor-like isoform X1

[tnl:113508080](https://www.genome.jp/entry/tnl:113508080) [K07207](https://www.genome.jp/entry/K07207) tuberin

 [**tnl04145**](https://www.genome.jp/kegg-bin/show_pathway?166859291342340/tnl04145.args) **Phagosome - Trichoplusia ni (cabbage looper) (**[**31**](javascript:display('tnl04145'))**)**

[tnl:113491902](https://www.genome.jp/entry/tnl:113491902) [K12182](https://www.genome.jp/entry/K12182) hepatocyte growth factor-regulated tyrosine kinase substrate

[tnl:113492607](https://www.genome.jp/entry/tnl:113492607) [K07897](https://www.genome.jp/entry/K07897) ras-related protein Rab-7a

[tnl:113492716](https://www.genome.jp/entry/tnl:113492716) [K04392](https://www.genome.jp/entry/K04392) ras-like GTP-binding protein RhoL

[tnl:113492751](https://www.genome.jp/entry/tnl:113492751) [K02144](https://www.genome.jp/entry/K02144) V-type proton ATPase subunit H isoform X1

[tnl:113493473](https://www.genome.jp/entry/tnl:113493473) [K02146](https://www.genome.jp/entry/K02146) V-type proton ATPase subunit d

[tnl:113494447](https://www.genome.jp/entry/tnl:113494447) [K07375](https://www.genome.jp/entry/K07375) tubulin beta chain-like

[tnl:113494681](https://www.genome.jp/entry/tnl:113494681) [K02147](https://www.genome.jp/entry/K02147) V-type proton ATPase subunit B

[tnl:113494689](https://www.genome.jp/entry/tnl:113494689) [K04392](https://www.genome.jp/entry/K04392) ras-related protein Rac1

[tnl:113496116](https://www.genome.jp/entry/tnl:113496116) [K02145](https://www.genome.jp/entry/K02145) V-type proton ATPase catalytic subunit A

[tnl:113496232](https://www.genome.jp/entry/tnl:113496232) [K02149](https://www.genome.jp/entry/K02149) V-type proton ATPase subunit D

[tnl:113497454](https://www.genome.jp/entry/tnl:113497454) [K13885](https://www.genome.jp/entry/K13885) protein peste-like isoform X1

[tnl:113497455](https://www.genome.jp/entry/tnl:113497455) [K13885](https://www.genome.jp/entry/K13885) protein croquemort-like

[tnl:113498276](https://www.genome.jp/entry/tnl:113498276) [K07374](https://www.genome.jp/entry/K07374) tubulin alpha chain

[tnl:113499397](https://www.genome.jp/entry/tnl:113499397) [K02150](https://www.genome.jp/entry/K02150) V-type proton ATPase subunit E

[tnl:113500508](https://www.genome.jp/entry/tnl:113500508) [K02151](https://www.genome.jp/entry/K02151) V-type proton ATPase subunit F

[tnl:113500605](https://www.genome.jp/entry/tnl:113500605) [K02154](https://www.genome.jp/entry/K02154) V-type proton ATPase 116 kDa subunit a

[tnl:113501292](https://www.genome.jp/entry/tnl:113501292) [K06528](https://www.genome.jp/entry/K06528) lysosome-associated membrane glycoprotein 1-like isoform X1

[tnl:113501446](https://www.genome.jp/entry/tnl:113501446) [K10415](https://www.genome.jp/entry/K10415) cytoplasmic dynein 1 intermediate chain isoform X1

[tnl:113502294](https://www.genome.jp/entry/tnl:113502294) [K08057](https://www.genome.jp/entry/K08057) calreticulin

[tnl:113502333](https://www.genome.jp/entry/tnl:113502333) [K03661](https://www.genome.jp/entry/K03661) V-type proton ATPase 21 kDa proteolipid subunit

[tnl:113503154](https://www.genome.jp/entry/tnl:113503154) [K02148](https://www.genome.jp/entry/K02148) V-type proton ATPase subunit C

[tnl:113503403](https://www.genome.jp/entry/tnl:113503403) [K10413](https://www.genome.jp/entry/K10413) dynein heavy chain, cytoplasmic isoform X1

[tnl:113504609](https://www.genome.jp/entry/tnl:113504609) [K05719](https://www.genome.jp/entry/K05719) integrin beta-PS

[tnl:113505484](https://www.genome.jp/entry/tnl:113505484) [K03662](https://www.genome.jp/entry/K03662) V-type proton ATPase subunit S1

[tnl:113505867](https://www.genome.jp/entry/tnl:113505867) [K08488](https://www.genome.jp/entry/K08488) syntaxin-7

[tnl:113505956](https://www.genome.jp/entry/tnl:113505956) [K04392](https://www.genome.jp/entry/K04392) ras-related C3 botulinum toxin substrate 1

[tnl:113506207](https://www.genome.jp/entry/tnl:113506207) [K02152](https://www.genome.jp/entry/K02152) V-type proton ATPase subunit G

[tnl:113506377](https://www.genome.jp/entry/tnl:113506377) [K01365](https://www.genome.jp/entry/K01365) cathepsin L-like

[tnl:113507896](https://www.genome.jp/entry/tnl:113507896) [K10416](https://www.genome.jp/entry/K10416) cytoplasmic dynein 1 light intermediate chain 2

[tnl:113508548](https://www.genome.jp/entry/tnl:113508548) [K08054](https://www.genome.jp/entry/K08054) calnexin

[tnl:113508948](https://www.genome.jp/entry/tnl:113508948) [K07375](https://www.genome.jp/entry/K07375) tubulin beta chain-like

 [**tnl04310**](https://www.genome.jp/kegg-bin/show_pathway?166859291342340/tnl04310.args) **Wnt signaling pathway - Trichoplusia ni (cabbage looper) (**[**28**](javascript:display('tnl04310'))**)**

[tnl:113492044](https://www.genome.jp/entry/tnl:113492044) [K04506](https://www.genome.jp/entry/K04506) LOW QUALITY PROTEIN: E3 ubiquitin-protein ligase siah-1-like

[tnl:113492716](https://www.genome.jp/entry/tnl:113492716) [K04392](https://www.genome.jp/entry/K04392) ras-like GTP-binding protein RhoL

[tnl:113493053](https://www.genome.jp/entry/tnl:113493053) [K08110](https://www.genome.jp/entry/K08110) glypican-6

[tnl:113494342](https://www.genome.jp/entry/tnl:113494342) [K04506](https://www.genome.jp/entry/K04506) E3 ubiquitin-protein ligase sina-like isoform X1

[tnl:113494689](https://www.genome.jp/entry/tnl:113494689) [K04392](https://www.genome.jp/entry/K04392) ras-related protein Rac1

[tnl:113495045](https://www.genome.jp/entry/tnl:113495045) [K04499](https://www.genome.jp/entry/K04499) ruvB-like helicase 1

[tnl:113496312](https://www.genome.jp/entry/tnl:113496312) [K03097](https://www.genome.jp/entry/K03097) casein kinase II subunit alpha isoform X1

[tnl:113496977](https://www.genome.jp/entry/tnl:113496977) [K05128](https://www.genome.jp/entry/K05128) tyrosine-protein kinase Drl

[tnl:113497125](https://www.genome.jp/entry/tnl:113497125) [K05858](https://www.genome.jp/entry/K05858) 1-phosphatidylinositol 4,5-bisphosphate phosphodiesterase classes I and II

[tnl:113497147](https://www.genome.jp/entry/tnl:113497147) [K05122](https://www.genome.jp/entry/K05122) tyrosine-protein kinase transmembrane receptor Ror

[tnl:113498219](https://www.genome.jp/entry/tnl:113498219) [K02375](https://www.genome.jp/entry/K02375) frizzled-2 isoform X1

[tnl:113499255](https://www.genome.jp/entry/tnl:113499255) [K04501](https://www.genome.jp/entry/K04501) mothers against decapentaplegic homolog 4 isoform X1

[tnl:113500056](https://www.genome.jp/entry/tnl:113500056) [K04510](https://www.genome.jp/entry/K04510) vang-like protein 1

[tnl:113500972](https://www.genome.jp/entry/tnl:113500972) [K02432](https://www.genome.jp/entry/K02432) frizzled-7-B-like

[tnl:113502491](https://www.genome.jp/entry/tnl:113502491) [K04515](https://www.genome.jp/entry/K04515) calcium/calmodulin-dependent protein kinase type II alpha chain

[tnl:113503427](https://www.genome.jp/entry/tnl:113503427) [K04513](https://www.genome.jp/entry/K04513) ras-like GTP-binding protein Rho1 isoform X1

[tnl:113503670](https://www.genome.jp/entry/tnl:113503670) [K04440](https://www.genome.jp/entry/K04440) stress-activated protein kinase JNK isoform X1

[tnl:113503848](https://www.genome.jp/entry/tnl:113503848) [K04512](https://www.genome.jp/entry/K04512) disheveled-associated activator of morphogenesis 1 isoform X1

[tnl:113503892](https://www.genome.jp/entry/tnl:113503892) [K02842](https://www.genome.jp/entry/K02842) frizzled-10-like

[tnl:113503987](https://www.genome.jp/entry/tnl:113503987) [K05858](https://www.genome.jp/entry/K05858) 1-phosphatidylinositol 4,5-bisphosphate phosphodiesterase isoform X1

[tnl:113505956](https://www.genome.jp/entry/tnl:113505956) [K04392](https://www.genome.jp/entry/K04392) ras-related C3 botulinum toxin substrate 1

[tnl:113506733](https://www.genome.jp/entry/tnl:113506733) [K04505](https://www.genome.jp/entry/K04505) presenilin homolog isoform X1

[tnl:113506804](https://www.genome.jp/entry/tnl:113506804) [K04345](https://www.genome.jp/entry/K04345) cAMP-dependent protein kinase catalytic subunit

[tnl:113506972](https://www.genome.jp/entry/tnl:113506972) [K04348](https://www.genome.jp/entry/K04348) serine/threonine-protein phosphatase 2B catalytic subunit 3-like

[tnl:113507780](https://www.genome.jp/entry/tnl:113507780) [K04507](https://www.genome.jp/entry/K04507) calcyclin-binding protein-like

[tnl:113507914](https://www.genome.jp/entry/tnl:113507914) [K02353](https://www.genome.jp/entry/K02353) LOW QUALITY PROTEIN: segment polarity protein dishevelled homolog DVL-3-like

[tnl:113507920](https://www.genome.jp/entry/tnl:113507920) [K02353](https://www.genome.jp/entry/K02353) LOW QUALITY PROTEIN: segment polarity protein dishevelled homolog DVL-3-like

[tnl:113508299](https://www.genome.jp/entry/tnl:113508299) [K03094](https://www.genome.jp/entry/K03094) S-phase kinase-associated protein 1

 [**tnl01232**](https://www.genome.jp/kegg-bin/show_pathway?166859291342340/tnl01232.args) **Nucleotide metabolism - Trichoplusia ni (cabbage looper) (**[**28**](javascript:display('tnl01232'))**)**

[tnl:113492544](https://www.genome.jp/entry/tnl:113492544) [K00940](https://www.genome.jp/entry/K00940) nucleoside diphosphate kinase

[tnl:113494314](https://www.genome.jp/entry/tnl:113494314) [K00364](https://www.genome.jp/entry/K00364) GMP reductase 1-like

[tnl:113494590](https://www.genome.jp/entry/tnl:113494590) [K00876](https://www.genome.jp/entry/K00876) probable uridine-cytidine kinase isoform X1

[tnl:113494755](https://www.genome.jp/entry/tnl:113494755) [K12304](https://www.genome.jp/entry/K12304) soluble calcium-activated nucleotidase 1

[tnl:113494797](https://www.genome.jp/entry/tnl:113494797) [K01756](https://www.genome.jp/entry/K01756) adenylosuccinate lyase-like

[tnl:113496055](https://www.genome.jp/entry/tnl:113496055) [K03783](https://www.genome.jp/entry/K03783) purine nucleoside phosphorylase-like isoform X1

[tnl:113496347](https://www.genome.jp/entry/tnl:113496347) [K10808](https://www.genome.jp/entry/K10808) ribonucleoside-diphosphate reductase subunit M2

[tnl:113496570](https://www.genome.jp/entry/tnl:113496570) [K00942](https://www.genome.jp/entry/K00942) guanylate kinase isoform X1

[tnl:113496813](https://www.genome.jp/entry/tnl:113496813) [K00939](https://www.genome.jp/entry/K00939) adenylate kinase

[tnl:113497121](https://www.genome.jp/entry/tnl:113497121) [K10807](https://www.genome.jp/entry/K10807) ribonucleoside-diphosphate reductase large subunit

[tnl:113497732](https://www.genome.jp/entry/tnl:113497732) [K00106](https://www.genome.jp/entry/K00106) xanthine dehydrogenase

[tnl:113497953](https://www.genome.jp/entry/tnl:113497953) [K01081](https://www.genome.jp/entry/K01081) cytosolic purine 5'-nucleotidase isoform X1

[tnl:113499175](https://www.genome.jp/entry/tnl:113499175) [K01487](https://www.genome.jp/entry/K01487) guanine deaminase-like

[tnl:113499305](https://www.genome.jp/entry/tnl:113499305) [K01487](https://www.genome.jp/entry/K01487) LOW QUALITY PROTEIN: guanine deaminase-like

[tnl:113499351](https://www.genome.jp/entry/tnl:113499351) [K01951](https://www.genome.jp/entry/K01951) GMP synthase [glutamine-hydrolyzing] isoform X1

[tnl:113499695](https://www.genome.jp/entry/tnl:113499695) [K01520](https://www.genome.jp/entry/K01520) deoxyuridine 5'-triphosphate nucleotidohydrolase

[tnl:113501491](https://www.genome.jp/entry/tnl:113501491) [K01519](https://www.genome.jp/entry/K01519) inosine triphosphate pyrophosphatase

[tnl:113501642](https://www.genome.jp/entry/tnl:113501642) [K07023](https://www.genome.jp/entry/K07023) HD domain-containing protein 2

[tnl:113501798](https://www.genome.jp/entry/tnl:113501798) [K18532](https://www.genome.jp/entry/K18532) adenylate kinase isoenzyme 6 homolog

[tnl:113503681](https://www.genome.jp/entry/tnl:113503681) [K00856](https://www.genome.jp/entry/K00856) adenosine kinase isoform X1

[tnl:113504794](https://www.genome.jp/entry/tnl:113504794) [K01490](https://www.genome.jp/entry/K01490) AMP deaminase 2-like isoform X1

[tnl:113504856](https://www.genome.jp/entry/tnl:113504856) [K01939](https://www.genome.jp/entry/K01939) adenylosuccinate synthetase-like

[tnl:113505327](https://www.genome.jp/entry/tnl:113505327) [K13800](https://www.genome.jp/entry/K13800) UMP-CMP kinase

[tnl:113507725](https://www.genome.jp/entry/tnl:113507725) [K01937](https://www.genome.jp/entry/K01937) CTP synthase

[tnl:113507969](https://www.genome.jp/entry/tnl:113507969) [K01939](https://www.genome.jp/entry/K01939) LOW QUALITY PROTEIN: adenylosuccinate synthetase-like

[tnl:113508501](https://www.genome.jp/entry/tnl:113508501) [K00560](https://www.genome.jp/entry/K00560) thymidylate synthase

[tnl:113508532](https://www.genome.jp/entry/tnl:113508532) [K00088](https://www.genome.jp/entry/K00088) inosine-5'-monophosphate dehydrogenase

[tnl:113508579](https://www.genome.jp/entry/tnl:113508579) [K24242](https://www.genome.jp/entry/K24242) 7-methylguanosine phosphate-specific 5'-nucleotidase

 [**tnl03013**](https://www.genome.jp/kegg-bin/show_pathway?166859291342340/tnl03013.args) **Nucleocytoplasmic transport - Trichoplusia ni (cabbage looper) (**[**27**](javascript:display('tnl03013'))**)**

[tnl:113491648](https://www.genome.jp/entry/tnl:113491648) [K14319](https://www.genome.jp/entry/K14319) ran GTPase-activating protein 1 isoform X1

[tnl:113491707](https://www.genome.jp/entry/tnl:113491707) [K07936](https://www.genome.jp/entry/K07936) GTP-binding nuclear protein Ran

[tnl:113491935](https://www.genome.jp/entry/tnl:113491935) [K14317](https://www.genome.jp/entry/K14317) nuclear pore complex protein Nup214

[tnl:113492859](https://www.genome.jp/entry/tnl:113492859) [K20222](https://www.genome.jp/entry/K20222) LOW QUALITY PROTEIN: importin-5-like

[tnl:113493063](https://www.genome.jp/entry/tnl:113493063) [K23583](https://www.genome.jp/entry/K23583) importin subunit alpha-3-like

[tnl:113493212](https://www.genome.jp/entry/tnl:113493212) [K14309](https://www.genome.jp/entry/K14309) nuclear pore complex protein Nup93-like

[tnl:113493477](https://www.genome.jp/entry/tnl:113493477) [K14293](https://www.genome.jp/entry/K14293) importin subunit beta-1

[tnl:113493667](https://www.genome.jp/entry/tnl:113493667) [K14310](https://www.genome.jp/entry/K14310) LOW QUALITY PROTEIN: nuclear pore complex protein Nup205

[tnl:113494111](https://www.genome.jp/entry/tnl:113494111) [K14323](https://www.genome.jp/entry/K14323) protein CASC3-like isoform X1

[tnl:113494292](https://www.genome.jp/entry/tnl:113494292) [K18423](https://www.genome.jp/entry/K18423) exportin-2

[tnl:113494401](https://www.genome.jp/entry/tnl:113494401) [K14298](https://www.genome.jp/entry/K14298) mRNA export factor

[tnl:113494550](https://www.genome.jp/entry/tnl:113494550) [K12812](https://www.genome.jp/entry/K12812) ATP-dependent RNA helicase WM6

[tnl:113497709](https://www.genome.jp/entry/tnl:113497709) [K15436](https://www.genome.jp/entry/K15436) transportin-3

[tnl:113497767](https://www.genome.jp/entry/tnl:113497767) [K14290](https://www.genome.jp/entry/K14290) exportin-1

[tnl:113498599](https://www.genome.jp/entry/tnl:113498599) [K15043](https://www.genome.jp/entry/K15043) importin subunit alpha-1-like isoform X1

[tnl:113499040](https://www.genome.jp/entry/tnl:113499040) [K12881](https://www.genome.jp/entry/K12881) THO complex subunit 4 isoform X1

[tnl:113499421](https://www.genome.jp/entry/tnl:113499421) [K14320](https://www.genome.jp/entry/K14320) aladin-like

[tnl:113500906](https://www.genome.jp/entry/tnl:113500906) [K15042](https://www.genome.jp/entry/K15042) importin subunit alpha-7

[tnl:113501787](https://www.genome.jp/entry/tnl:113501787) [K14294](https://www.genome.jp/entry/K14294) partner of Y14 and mago

[tnl:113501794](https://www.genome.jp/entry/tnl:113501794) [K03231](https://www.genome.jp/entry/K03231) elongation factor 1-alpha

[tnl:113501866](https://www.genome.jp/entry/tnl:113501866) [K14289](https://www.genome.jp/entry/K14289) exportin-5

[tnl:113502270](https://www.genome.jp/entry/tnl:113502270) [K14313](https://www.genome.jp/entry/K14313) nucleoporin Nup35

[tnl:113504067](https://www.genome.jp/entry/tnl:113504067) [K12876](https://www.genome.jp/entry/K12876) RNA-binding protein 8A

[tnl:113505023](https://www.genome.jp/entry/tnl:113505023) [K14004](https://www.genome.jp/entry/K14004) protein SEC13 homolog

[tnl:113505369](https://www.genome.jp/entry/tnl:113505369) [K12160](https://www.genome.jp/entry/K12160) small ubiquitin-related modifier 3

[tnl:113508311](https://www.genome.jp/entry/tnl:113508311) [K18655](https://www.genome.jp/entry/K18655) DEAD-box helicase Dbp80

[tnl:113508374](https://www.genome.jp/entry/tnl:113508374) [K20223](https://www.genome.jp/entry/K20223) importin-7 isoform X1

 [**tnl04120**](https://www.genome.jp/kegg-bin/show_pathway?166859291342340/tnl04120.args) **Ubiquitin mediated proteolysis - Trichoplusia ni (cabbage looper) (**[**26**](javascript:display('tnl04120'))**)**

[tnl:113491770](https://www.genome.jp/entry/tnl:113491770) [K10588](https://www.genome.jp/entry/K10588) ubiquitin-protein ligase E3B

[tnl:113491887](https://www.genome.jp/entry/tnl:113491887) [K04678](https://www.genome.jp/entry/K04678) E3 ubiquitin-protein ligase SMURF2

[tnl:113492044](https://www.genome.jp/entry/tnl:113492044) [K04506](https://www.genome.jp/entry/K04506) LOW QUALITY PROTEIN: E3 ubiquitin-protein ligase siah-1-like

[tnl:113492542](https://www.genome.jp/entry/tnl:113492542) [K10579](https://www.genome.jp/entry/K10579) NEDD8-conjugating enzyme Ubc12

[tnl:113493415](https://www.genome.jp/entry/tnl:113493415) [K03873](https://www.genome.jp/entry/K03873) elongin-B

[tnl:113494342](https://www.genome.jp/entry/tnl:113494342) [K04506](https://www.genome.jp/entry/K04506) E3 ubiquitin-protein ligase sina-like isoform X1

[tnl:113494695](https://www.genome.jp/entry/tnl:113494695) [K09561](https://www.genome.jp/entry/K09561) E3 ubiquitin-protein ligase CHIP

[tnl:113497745](https://www.genome.jp/entry/tnl:113497745) [K06689](https://www.genome.jp/entry/K06689) ubiquitin-conjugating enzyme E2-17 kDa

[tnl:113497933](https://www.genome.jp/entry/tnl:113497933) [K10576](https://www.genome.jp/entry/K10576) ubiquitin-conjugating enzyme E2 H

[tnl:113498056](https://www.genome.jp/entry/tnl:113498056) [K10688](https://www.genome.jp/entry/K10688) ubiquitin-conjugating enzyme E2 W

[tnl:113498293](https://www.genome.jp/entry/tnl:113498293) [K10612](https://www.genome.jp/entry/K10612) cullin-5-like

[tnl:113501076](https://www.genome.jp/entry/tnl:113501076) [K03869](https://www.genome.jp/entry/K03869) cullin-3 isoform X1

[tnl:113501517](https://www.genome.jp/entry/tnl:113501517) [K10687](https://www.genome.jp/entry/K10687) NEDD8-conjugating enzyme UBE2F-like

[tnl:113501562](https://www.genome.jp/entry/tnl:113501562) [K05633](https://www.genome.jp/entry/K05633) E3 ubiquitin-protein ligase Su(dx)

[tnl:113501820](https://www.genome.jp/entry/tnl:113501820) [K03178](https://www.genome.jp/entry/K03178) ubiquitin-like modifier-activating enzyme 1

[tnl:113502276](https://www.genome.jp/entry/tnl:113502276) [K03870](https://www.genome.jp/entry/K03870) cullin-2

[tnl:113502801](https://www.genome.jp/entry/tnl:113502801) [K10575](https://www.genome.jp/entry/K10575) ubiquitin-conjugating enzyme E2 G1-like isoform X1

[tnl:113503065](https://www.genome.jp/entry/tnl:113503065) [K04552](https://www.genome.jp/entry/K04552) ubiquitin-conjugating enzyme E2 L3

[tnl:113503581](https://www.genome.jp/entry/tnl:113503581) [K08770](https://www.genome.jp/entry/K08770) polyubiquitin-A

[tnl:113505171](https://www.genome.jp/entry/tnl:113505171) [K04706](https://www.genome.jp/entry/K04706) E3 SUMO-protein ligase PIAS2 isoform X1

[tnl:113505180](https://www.genome.jp/entry/tnl:113505180) [K04707](https://www.genome.jp/entry/K04707) E3 ubiquitin-protein ligase CBL-B-B isoform X1

[tnl:113506374](https://www.genome.jp/entry/tnl:113506374) [K02977](https://www.genome.jp/entry/K02977) ubiquitin-40S ribosomal protein S27a

[tnl:113506541](https://www.genome.jp/entry/tnl:113506541) [K10575](https://www.genome.jp/entry/K10575) ubiquitin-conjugating enzyme E2 G1-like

[tnl:113507644](https://www.genome.jp/entry/tnl:113507644) [K10597](https://www.genome.jp/entry/K10597) ubiquitin conjugation factor E4 B

[tnl:113508299](https://www.genome.jp/entry/tnl:113508299) [K03094](https://www.genome.jp/entry/K03094) S-phase kinase-associated protein 1

[tnl:113508721](https://www.genome.jp/entry/tnl:113508721) [K10581](https://www.genome.jp/entry/K10581) (E3-independent) E2 ubiquitin-conjugating enzyme

 [**tnl01230**](https://www.genome.jp/kegg-bin/show_pathway?166859291342340/tnl01230.args) **Biosynthesis of amino acids - Trichoplusia ni (cabbage looper) (**[**24**](javascript:display('tnl01230'))**)**

[tnl:113491873](https://www.genome.jp/entry/tnl:113491873) [K00600](https://www.genome.jp/entry/K00600) serine hydroxymethyltransferase, cytosolic isoform X1

[tnl:113492710](https://www.genome.jp/entry/tnl:113492710) [K00031](https://www.genome.jp/entry/K00031) isocitrate dehydrogenase [NADP] cytoplasmic-like

[tnl:113492937](https://www.genome.jp/entry/tnl:113492937) [K14454](https://www.genome.jp/entry/K14454) aspartate aminotransferase, cytoplasmic

[tnl:113493828](https://www.genome.jp/entry/tnl:113493828) [K00948](https://www.genome.jp/entry/K00948) ribose-phosphate pyrophosphokinase 2

[tnl:113493924](https://www.genome.jp/entry/tnl:113493924) [K01803](https://www.genome.jp/entry/K01803) triosephosphate isomerase

[tnl:113494495](https://www.genome.jp/entry/tnl:113494495) [K00927](https://www.genome.jp/entry/K00927) phosphoglycerate kinase

[tnl:113495260](https://www.genome.jp/entry/tnl:113495260) [K01953](https://www.genome.jp/entry/K01953) asparagine synthetase [glutamine-hydrolyzing]

[tnl:113496121](https://www.genome.jp/entry/tnl:113496121) [K01915](https://www.genome.jp/entry/K01915) glutamine synthetase 2 cytoplasmic-like isoform X1

[tnl:113496177](https://www.genome.jp/entry/tnl:113496177) [K00058](https://www.genome.jp/entry/K00058) D-3-phosphoglycerate dehydrogenase

[tnl:113496310](https://www.genome.jp/entry/tnl:113496310) [K00850](https://www.genome.jp/entry/K00850) ATP-dependent 6-phosphofructokinase isoform X1

[tnl:113496805](https://www.genome.jp/entry/tnl:113496805) [K00286](https://www.genome.jp/entry/K00286) pyrroline-5-carboxylate reductase-like

[tnl:113498369](https://www.genome.jp/entry/tnl:113498369) [K00814](https://www.genome.jp/entry/K00814) alanine aminotransferase 1-like isoform X1

[tnl:113499004](https://www.genome.jp/entry/tnl:113499004) [K01689](https://www.genome.jp/entry/K01689) enolase

[tnl:113499721](https://www.genome.jp/entry/tnl:113499721) [K00615](https://www.genome.jp/entry/K00615) transketolase-like protein 2

[tnl:113500423](https://www.genome.jp/entry/tnl:113500423) [K00873](https://www.genome.jp/entry/K00873) pyruvate kinase-like isoform X1

[tnl:113500574](https://www.genome.jp/entry/tnl:113500574) [K00831](https://www.genome.jp/entry/K00831) probable phosphoserine aminotransferase

[tnl:113500928](https://www.genome.jp/entry/tnl:113500928) [K00789](https://www.genome.jp/entry/K00789) S-adenosylmethionine synthase isoform X1

[tnl:113502164](https://www.genome.jp/entry/tnl:113502164) [K01758](https://www.genome.jp/entry/K01758) cystathionine gamma-lyase-like

[tnl:113504438](https://www.genome.jp/entry/tnl:113504438) [K00031](https://www.genome.jp/entry/K00031) isocitrate dehydrogenase [NADP] cytoplasmic isoform X1

[tnl:113504890](https://www.genome.jp/entry/tnl:113504890) [K01697](https://www.genome.jp/entry/K01697) cystathionine beta-synthase-like

[tnl:113506529](https://www.genome.jp/entry/tnl:113506529) [K01834](https://www.genome.jp/entry/K01834) phosphoglycerate mutase 2-like

[tnl:113508324](https://www.genome.jp/entry/tnl:113508324) [K00616](https://www.genome.jp/entry/K00616) probable transaldolase

[tnl:113508342](https://www.genome.jp/entry/tnl:113508342) [K01807](https://www.genome.jp/entry/K01807) ribose-5-phosphate isomerase

[tnl:113509001](https://www.genome.jp/entry/tnl:113509001) [K00134](https://www.genome.jp/entry/K00134) glyceraldehyde-3-phosphate dehydrogenase

 [**tnl04391**](https://www.genome.jp/kegg-bin/show_pathway?166859291342340/tnl04391.args) **Hippo signaling pathway - fly - Trichoplusia ni (cabbage looper) (**[**23**](javascript:display('tnl04391'))**)**

[tnl:113492109](https://www.genome.jp/entry/tnl:113492109) [K06094](https://www.genome.jp/entry/K06094) lethal(2) giant larvae protein isoform X1

[tnl:113493261](https://www.genome.jp/entry/tnl:113493261) [K16682](https://www.genome.jp/entry/K16682) LIM domain-containing protein jub

[tnl:113493343](https://www.genome.jp/entry/tnl:113493343) [K06685](https://www.genome.jp/entry/K06685) MOB kinase activator-like 1

[tnl:113494124](https://www.genome.jp/entry/tnl:113494124) [K06093](https://www.genome.jp/entry/K06093) partitioning defective protein 6-like

[tnl:113494680](https://www.genome.jp/entry/tnl:113494680) [K03456](https://www.genome.jp/entry/K03456) serine/threonine-protein phosphatase PP2A 65 kDa regulatory subunit

[tnl:113495361](https://www.genome.jp/entry/tnl:113495361) [K04354](https://www.genome.jp/entry/K04354) serine/threonine-protein phosphatase 2A 55 kDa regulatory subunit B alpha isoform isoform X1

[tnl:113496323](https://www.genome.jp/entry/tnl:113496323) [K16197](https://www.genome.jp/entry/K16197) 14-3-3 protein zeta isoform X1

[tnl:113496563](https://www.genome.jp/entry/tnl:113496563) [K06095](https://www.genome.jp/entry/K06095) patj homolog

[tnl:113496801](https://www.genome.jp/entry/tnl:113496801) [K16175](https://www.genome.jp/entry/K16175) protein lap4 isoform X1

[tnl:113497312](https://www.genome.jp/entry/tnl:113497312) [K04676](https://www.genome.jp/entry/K04676) protein mothers against dpp

[tnl:113497791](https://www.genome.jp/entry/tnl:113497791) [K12076](https://www.genome.jp/entry/K12076) disks large 1 tumor suppressor protein isoform X1

[tnl:113497795](https://www.genome.jp/entry/tnl:113497795) [K16680](https://www.genome.jp/entry/K16680) hemicentin-2

[tnl:113497825](https://www.genome.jp/entry/tnl:113497825) [K24050](https://www.genome.jp/entry/K24050) disks large homolog 5

[tnl:113499516](https://www.genome.jp/entry/tnl:113499516) [K04382](https://www.genome.jp/entry/K04382) serine/threonine-protein phosphatase 2A catalytic subunit beta isoform

[tnl:113500953](https://www.genome.jp/entry/tnl:113500953) [K04412](https://www.genome.jp/entry/K04412) serine/threonine-protein kinase 4 isoform X1

[tnl:113502200](https://www.genome.jp/entry/tnl:113502200) [K16687](https://www.genome.jp/entry/K16687) transcriptional coactivator YAP1-like isoform X1

[tnl:113502799](https://www.genome.jp/entry/tnl:113502799) [K16684](https://www.genome.jp/entry/K16684) moesin/ezrin/radixin homolog 2 isoform X1

[tnl:113503197](https://www.genome.jp/entry/tnl:113503197) [K06630](https://www.genome.jp/entry/K06630) 14-3-3 protein epsilon

[tnl:113503670](https://www.genome.jp/entry/tnl:113503670) [K04440](https://www.genome.jp/entry/K04440) stress-activated protein kinase JNK isoform X1

[tnl:113504720](https://www.genome.jp/entry/tnl:113504720) [K16672](https://www.genome.jp/entry/K16672) homeobox protein homothorax isoform X1

[tnl:113505824](https://www.genome.jp/entry/tnl:113505824) [K16676](https://www.genome.jp/entry/K16676) lipoma-preferred partner homolog

[tnl:113506351](https://www.genome.jp/entry/tnl:113506351) [K04237](https://www.genome.jp/entry/K04237) partitioning defective 3 homolog isoform X1

[tnl:113506488](https://www.genome.jp/entry/tnl:113506488) [K16675](https://www.genome.jp/entry/K16675) palmitoyltransferase ZDHHC18-like

 [**tnl00480**](https://www.genome.jp/kegg-bin/show_pathway?166859291342340/tnl00480.args) **Glutathione metabolism - Trichoplusia ni (cabbage looper) (**[**23**](javascript:display('tnl00480'))**)**

[tnl:113492710](https://www.genome.jp/entry/tnl:113492710) [K00031](https://www.genome.jp/entry/K00031) isocitrate dehydrogenase [NADP] cytoplasmic-like

[tnl:113492726](https://www.genome.jp/entry/tnl:113492726) [K11204](https://www.genome.jp/entry/K11204) glutamate--cysteine ligase catalytic subunit

[tnl:113494140](https://www.genome.jp/entry/tnl:113494140) [K01469](https://www.genome.jp/entry/K01469) 5-oxoprolinase

[tnl:113494198](https://www.genome.jp/entry/tnl:113494198) [K11205](https://www.genome.jp/entry/K11205) glutamate--cysteine ligase regulatory subunit isoform X1

[tnl:113494305](https://www.genome.jp/entry/tnl:113494305) [K21456](https://www.genome.jp/entry/K21456) glutathione synthetase-like isoform X1

[tnl:113494907](https://www.genome.jp/entry/tnl:113494907) [K00799](https://www.genome.jp/entry/K00799) glutathione S-transferase 1-like

[tnl:113495619](https://www.genome.jp/entry/tnl:113495619) [K11188](https://www.genome.jp/entry/K11188) peroxiredoxin-6

[tnl:113496347](https://www.genome.jp/entry/tnl:113496347) [K10808](https://www.genome.jp/entry/K10808) ribonucleoside-diphosphate reductase subunit M2

[tnl:113496731](https://www.genome.jp/entry/tnl:113496731) [K00799](https://www.genome.jp/entry/K00799) uncharacterized protein LOC113496731

[tnl:113497008](https://www.genome.jp/entry/tnl:113497008) [K00799](https://www.genome.jp/entry/K00799) glutathione S-transferase 1-1-like

[tnl:113497009](https://www.genome.jp/entry/tnl:113497009) [K00799](https://www.genome.jp/entry/K00799) glutathione S-transferase 1-1-like isoform X1

[tnl:113497121](https://www.genome.jp/entry/tnl:113497121) [K10807](https://www.genome.jp/entry/K10807) ribonucleoside-diphosphate reductase large subunit

[tnl:113497263](https://www.genome.jp/entry/tnl:113497263) [K04097](https://www.genome.jp/entry/K04097) glutathione S-transferase 2-like

[tnl:113497424](https://www.genome.jp/entry/tnl:113497424) [K00797](https://www.genome.jp/entry/K00797) spermidine synthase

[tnl:113499552](https://www.genome.jp/entry/tnl:113499552) [K00799](https://www.genome.jp/entry/K00799) uncharacterized protein LOC113499552

[tnl:113501060](https://www.genome.jp/entry/tnl:113501060) [K00036](https://www.genome.jp/entry/K00036) glucose-6-phosphate 1-dehydrogenase

[tnl:113501099](https://www.genome.jp/entry/tnl:113501099) [K00310](https://www.genome.jp/entry/K00310) pyrimidodiazepine synthase-like

[tnl:113501843](https://www.genome.jp/entry/tnl:113501843) [K00682](https://www.genome.jp/entry/K00682) gamma-glutamylcyclotransferase-like isoform X1

[tnl:113502268](https://www.genome.jp/entry/tnl:113502268) [K00802](https://www.genome.jp/entry/K00802) spermine synthase

[tnl:113504438](https://www.genome.jp/entry/tnl:113504438) [K00031](https://www.genome.jp/entry/K00031) isocitrate dehydrogenase [NADP] cytoplasmic isoform X1

[tnl:113506144](https://www.genome.jp/entry/tnl:113506144) [K01255](https://www.genome.jp/entry/K01255) putative aminopeptidase W07G4.4

[tnl:113507103](https://www.genome.jp/entry/tnl:113507103) [K00799](https://www.genome.jp/entry/K00799) glutathione S-transferase 1-1-like

[tnl:113508050](https://www.genome.jp/entry/tnl:113508050) [K00799](https://www.genome.jp/entry/K00799) glutathione S-transferase 1-1-like

 [**tnl04213**](https://www.genome.jp/kegg-bin/show_pathway?166859291342340/tnl04213.args) **Longevity regulating pathway - multiple species - Trichoplusia ni (cabbage looper) (**[**22**](javascript:display('tnl04213'))**)**

[tnl:113492520](https://www.genome.jp/entry/tnl:113492520) [K08339](https://www.genome.jp/entry/K08339) autophagy protein 5

[tnl:113493497](https://www.genome.jp/entry/tnl:113493497) [K07200](https://www.genome.jp/entry/K07200) 5'-AMP-activated protein kinase subunit gamma-2-like

[tnl:113493805](https://www.genome.jp/entry/tnl:113493805) [K07827](https://www.genome.jp/entry/K07827) GTPase HRas

[tnl:113495560](https://www.genome.jp/entry/tnl:113495560) [K03283](https://www.genome.jp/entry/K03283) heat shock protein 68-like

[tnl:113495656](https://www.genome.jp/entry/tnl:113495656) [K04456](https://www.genome.jp/entry/K04456) RAC serine/threonine-protein kinase

[tnl:113495760](https://www.genome.jp/entry/tnl:113495760) [K03781](https://www.genome.jp/entry/K03781) catalase

[tnl:113496173](https://www.genome.jp/entry/tnl:113496173) [K07200](https://www.genome.jp/entry/K07200) uncharacterized protein LOC113496173 isoform X1

[tnl:113496267](https://www.genome.jp/entry/tnl:113496267) [K03283](https://www.genome.jp/entry/K03283) heat shock protein 68-like

[tnl:113496483](https://www.genome.jp/entry/tnl:113496483) [K06067](https://www.genome.jp/entry/K06067) LOW QUALITY PROTEIN: histone deacetylase Rpd3-like

[tnl:113496752](https://www.genome.jp/entry/tnl:113496752) [K06067](https://www.genome.jp/entry/K06067) histone deacetylase Rpd3-like

[tnl:113497553](https://www.genome.jp/entry/tnl:113497553) [K07199](https://www.genome.jp/entry/K07199) 5'-AMP-activated protein kinase subunit beta-1 isoform X1

[tnl:113498138](https://www.genome.jp/entry/tnl:113498138) [K09542](https://www.genome.jp/entry/K09542) protein lethal(2)essential for life-like

[tnl:113499468](https://www.genome.jp/entry/tnl:113499468) [K18644](https://www.genome.jp/entry/K18644) eukaryotic translation initiation factor 4E-binding protein 3

[tnl:113501500](https://www.genome.jp/entry/tnl:113501500) [K08041](https://www.genome.jp/entry/K08041) Ca(2+)/calmodulin-responsive adenylate cyclase-like

[tnl:113503039](https://www.genome.jp/entry/tnl:113503039) [K07198](https://www.genome.jp/entry/K07198) 5'-AMP-activated protein kinase catalytic subunit alpha-2 isoform X1

[tnl:113504422](https://www.genome.jp/entry/tnl:113504422) [K04688](https://www.genome.jp/entry/K04688) ribosomal protein S6 kinase beta-2

[tnl:113504501](https://www.genome.jp/entry/tnl:113504501) [K04565](https://www.genome.jp/entry/K04565) superoxide dismutase [Cu-Zn] 2-like

[tnl:113505811](https://www.genome.jp/entry/tnl:113505811) [K04565](https://www.genome.jp/entry/K04565) superoxide dismutase [Cu-Zn]

[tnl:113506423](https://www.genome.jp/entry/tnl:113506423) [K03283](https://www.genome.jp/entry/K03283) heat shock 70 kDa protein cognate 4

[tnl:113506804](https://www.genome.jp/entry/tnl:113506804) [K04345](https://www.genome.jp/entry/K04345) cAMP-dependent protein kinase catalytic subunit

[tnl:113507623](https://www.genome.jp/entry/tnl:113507623) [K07203](https://www.genome.jp/entry/K07203) serine/threonine-protein kinase Tor-like isoform X1

[tnl:113507970](https://www.genome.jp/entry/tnl:113507970) [K08049](https://www.genome.jp/entry/K08049) adenylate cyclase type 9

 [**tnl04068**](https://www.genome.jp/kegg-bin/show_pathway?166859291342340/tnl04068.args) **FoxO signaling pathway - Trichoplusia ni (cabbage looper) (**[**22**](javascript:display('tnl04068'))**)**

[tnl:113491757](https://www.genome.jp/entry/tnl:113491757) [K04371](https://www.genome.jp/entry/K04371) mitogen-activated protein kinase ERK-A

[tnl:113492015](https://www.genome.jp/entry/tnl:113492015) [K04364](https://www.genome.jp/entry/K04364) protein enhancer of sevenless 2B

[tnl:113492018](https://www.genome.jp/entry/tnl:113492018) [K11838](https://www.genome.jp/entry/K11838) ubiquitin carboxyl-terminal hydrolase 7 isoform X1

[tnl:113492473](https://www.genome.jp/entry/tnl:113492473) [K11434](https://www.genome.jp/entry/K11434) protein arginine N-methyltransferase 1

[tnl:113492842](https://www.genome.jp/entry/tnl:113492842) [K06631](https://www.genome.jp/entry/K06631) serine/threonine-protein kinase polo isoform X1

[tnl:113493497](https://www.genome.jp/entry/tnl:113493497) [K07200](https://www.genome.jp/entry/K07200) 5'-AMP-activated protein kinase subunit gamma-2-like

[tnl:113493668](https://www.genome.jp/entry/tnl:113493668) [K04365](https://www.genome.jp/entry/K04365) raf homolog serine/threonine-protein kinase Raf isoform X1

[tnl:113493805](https://www.genome.jp/entry/tnl:113493805) [K07827](https://www.genome.jp/entry/K07827) GTPase HRas

[tnl:113495656](https://www.genome.jp/entry/tnl:113495656) [K04456](https://www.genome.jp/entry/K04456) RAC serine/threonine-protein kinase

[tnl:113495760](https://www.genome.jp/entry/tnl:113495760) [K03781](https://www.genome.jp/entry/K03781) catalase

[tnl:113495885](https://www.genome.jp/entry/tnl:113495885) [K08341](https://www.genome.jp/entry/K08341) gamma-aminobutyric acid receptor-associated protein

[tnl:113496173](https://www.genome.jp/entry/tnl:113496173) [K07200](https://www.genome.jp/entry/K07200) uncharacterized protein LOC113496173 isoform X1

[tnl:113497553](https://www.genome.jp/entry/tnl:113497553) [K07199](https://www.genome.jp/entry/K07199) 5'-AMP-activated protein kinase subunit beta-1 isoform X1

[tnl:113497664](https://www.genome.jp/entry/tnl:113497664) [K01110](https://www.genome.jp/entry/K01110) phosphatidylinositol 3,4,5-trisphosphate 3-phosphatase and dual-specificity protein phosphatase PTEN

[tnl:113499255](https://www.genome.jp/entry/tnl:113499255) [K04501](https://www.genome.jp/entry/K04501) mothers against decapentaplegic homolog 4 isoform X1

[tnl:113501942](https://www.genome.jp/entry/tnl:113501942) [K04441](https://www.genome.jp/entry/K04441) mitogen-activated protein kinase p38b-like isoform X1

[tnl:113502394](https://www.genome.jp/entry/tnl:113502394) [K07298](https://www.genome.jp/entry/K07298) serine/threonine-protein kinase STK11 isoform X1

[tnl:113502760](https://www.genome.jp/entry/tnl:113502760) [K15010](https://www.genome.jp/entry/K15010) homer protein homolog 2 isoform X1

[tnl:113503039](https://www.genome.jp/entry/tnl:113503039) [K07198](https://www.genome.jp/entry/K07198) 5'-AMP-activated protein kinase catalytic subunit alpha-2 isoform X1

[tnl:113503523](https://www.genome.jp/entry/tnl:113503523) [K04368](https://www.genome.jp/entry/K04368) dual specificity mitogen-activated protein kinase kinase dSOR1

[tnl:113503670](https://www.genome.jp/entry/tnl:113503670) [K04440](https://www.genome.jp/entry/K04440) stress-activated protein kinase JNK isoform X1

[tnl:113505189](https://www.genome.jp/entry/tnl:113505189) [K04674](https://www.genome.jp/entry/K04674) TGF-beta receptor type-1-like isoform X1

 [**tnl00970**](https://www.genome.jp/kegg-bin/show_pathway?166859291342340/tnl00970.args) **Aminoacyl-tRNA biosynthesis - Trichoplusia ni (cabbage looper) (**[**21**](javascript:display('tnl00970'))**)**

[tnl:113491923](https://www.genome.jp/entry/tnl:113491923) [K01873](https://www.genome.jp/entry/K01873) valine--tRNA ligase

[tnl:113493447](https://www.genome.jp/entry/tnl:113493447) [K04567](https://www.genome.jp/entry/K04567) lysine--tRNA ligase isoform X1

[tnl:113494610](https://www.genome.jp/entry/tnl:113494610) [K01880](https://www.genome.jp/entry/K01880) glycine--tRNA ligase

[tnl:113496381](https://www.genome.jp/entry/tnl:113496381) [K01889](https://www.genome.jp/entry/K01889) phenylalanine--tRNA ligase alpha subunit

[tnl:113497820](https://www.genome.jp/entry/tnl:113497820) [K01887](https://www.genome.jp/entry/K01887) arginine--tRNA ligase, cytoplasmic

[tnl:113498448](https://www.genome.jp/entry/tnl:113498448) [K14163](https://www.genome.jp/entry/K14163) bifunctional glutamate/proline--tRNA ligase isoform X1

[tnl:113498613](https://www.genome.jp/entry/tnl:113498613) [K01867](https://www.genome.jp/entry/K01867) tryptophan--tRNA ligase, cytoplasmic

[tnl:113499807](https://www.genome.jp/entry/tnl:113499807) [K22503](https://www.genome.jp/entry/K22503) LOW QUALITY PROTEIN: aspartate--tRNA ligase, cytoplasmic-like

[tnl:113501557](https://www.genome.jp/entry/tnl:113501557) [K01872](https://www.genome.jp/entry/K01872) alanine--tRNA ligase, cytoplasmic

[tnl:113502506](https://www.genome.jp/entry/tnl:113502506) [K01868](https://www.genome.jp/entry/K01868) threonine--tRNA ligase, cytoplasmic isoform X1

[tnl:113503013](https://www.genome.jp/entry/tnl:113503013) [K01893](https://www.genome.jp/entry/K01893) asparagine--tRNA ligase, cytoplasmic

[tnl:113503429](https://www.genome.jp/entry/tnl:113503429) [K01866](https://www.genome.jp/entry/K01866) tyrosine--tRNA ligase, cytoplasmic

[tnl:113503469](https://www.genome.jp/entry/tnl:113503469) [K01870](https://www.genome.jp/entry/K01870) isoleucine--tRNA ligase, cytoplasmic

[tnl:113505465](https://www.genome.jp/entry/tnl:113505465) [K01875](https://www.genome.jp/entry/K01875) serine--tRNA ligase, cytoplasmic

[tnl:113507036](https://www.genome.jp/entry/tnl:113507036) [K22503](https://www.genome.jp/entry/K22503) aspartate--tRNA ligase, cytoplasmic-like

[tnl:113507104](https://www.genome.jp/entry/tnl:113507104) [K01890](https://www.genome.jp/entry/K01890) phenylalanine--tRNA ligase beta subunit isoform X1

[tnl:113507470](https://www.genome.jp/entry/tnl:113507470) [K01874](https://www.genome.jp/entry/K01874) methionine--tRNA ligase, cytoplasmic

[tnl:113508287](https://www.genome.jp/entry/tnl:113508287) [K01883](https://www.genome.jp/entry/K01883) cysteine--tRNA ligase, cytoplasmic

[tnl:113508384](https://www.genome.jp/entry/tnl:113508384) [K01886](https://www.genome.jp/entry/K01886) probable glutamine--tRNA ligase

[tnl:113508450](https://www.genome.jp/entry/tnl:113508450) [K01892](https://www.genome.jp/entry/K01892) histidine--tRNA ligase, cytoplasmic isoform X1

[tnl:113508534](https://www.genome.jp/entry/tnl:113508534) [K01869](https://www.genome.jp/entry/K01869) leucine--tRNA ligase, cytoplasmic

 [**tnl03015**](https://www.genome.jp/kegg-bin/show_pathway?166859291342340/tnl03015.args) **mRNA surveillance pathway - Trichoplusia ni (cabbage looper) (**[**19**](javascript:display('tnl03015'))**)**

[tnl:113491975](https://www.genome.jp/entry/tnl:113491975) [K03267](https://www.genome.jp/entry/K03267) eukaryotic peptide chain release factor GTP-binding subunit ERF3A isoform X1

[tnl:113494111](https://www.genome.jp/entry/tnl:113494111) [K14323](https://www.genome.jp/entry/K14323) protein CASC3-like isoform X1

[tnl:113494550](https://www.genome.jp/entry/tnl:113494550) [K12812](https://www.genome.jp/entry/K12812) ATP-dependent RNA helicase WM6

[tnl:113494680](https://www.genome.jp/entry/tnl:113494680) [K03456](https://www.genome.jp/entry/K03456) serine/threonine-protein phosphatase PP2A 65 kDa regulatory subunit

[tnl:113495361](https://www.genome.jp/entry/tnl:113495361) [K04354](https://www.genome.jp/entry/K04354) serine/threonine-protein phosphatase 2A 55 kDa regulatory subunit B alpha isoform isoform X1

[tnl:113495718](https://www.genome.jp/entry/tnl:113495718) [K06269](https://www.genome.jp/entry/K06269) serine/threonine-protein phosphatase PP1-beta catalytic subunit

[tnl:113496349](https://www.genome.jp/entry/tnl:113496349) [K13126](https://www.genome.jp/entry/K13126) polyadenylate-binding protein 1-A

[tnl:113498663](https://www.genome.jp/entry/tnl:113498663) [K11584](https://www.genome.jp/entry/K11584) serine/threonine-protein phosphatase 2A 56 kDa regulatory subunit gamma isoform-like isoform X1

[tnl:113499040](https://www.genome.jp/entry/tnl:113499040) [K12881](https://www.genome.jp/entry/K12881) THO complex subunit 4 isoform X1

[tnl:113499516](https://www.genome.jp/entry/tnl:113499516) [K04382](https://www.genome.jp/entry/K04382) serine/threonine-protein phosphatase 2A catalytic subunit beta isoform

[tnl:113500207](https://www.genome.jp/entry/tnl:113500207) [K14398](https://www.genome.jp/entry/K14398) cleavage and polyadenylation specificity factor subunit CG7185

[tnl:113501787](https://www.genome.jp/entry/tnl:113501787) [K14294](https://www.genome.jp/entry/K14294) partner of Y14 and mago

[tnl:113502995](https://www.genome.jp/entry/tnl:113502995) [K06269](https://www.genome.jp/entry/K06269) serine/threonine-protein phosphatase PP1-gamma catalytic subunit B-like

[tnl:113503328](https://www.genome.jp/entry/tnl:113503328) [K14411](https://www.genome.jp/entry/K14411) heterogeneous nuclear ribonucleoprotein 27C isoform X1

[tnl:113504067](https://www.genome.jp/entry/tnl:113504067) [K12876](https://www.genome.jp/entry/K12876) RNA-binding protein 8A

[tnl:113504892](https://www.genome.jp/entry/tnl:113504892) [K11584](https://www.genome.jp/entry/K11584) serine/threonine-protein phosphatase 2A 56 kDa regulatory subunit epsilon isoform

[tnl:113505522](https://www.genome.jp/entry/tnl:113505522) [K03265](https://www.genome.jp/entry/K03265) eukaryotic peptide chain release factor subunit 1 isoform X1

[tnl:113506083](https://www.genome.jp/entry/tnl:113506083) [K13098](https://www.genome.jp/entry/K13098) RNA-binding protein cabeza isoform X1

[tnl:113508311](https://www.genome.jp/entry/tnl:113508311) [K18655](https://www.genome.jp/entry/K18655) DEAD-box helicase Dbp80

**** [**tnl03008**](https://www.genome.jp/kegg-bin/show_pathway?166859291342340/tnl03008.args) **Ribosome biogenesis in eukaryotes - Trichoplusia ni (cabbage looper) (**[**19**](javascript:display('tnl03008'))**)**

[tnl:113491707](https://www.genome.jp/entry/tnl:113491707) [K07936](https://www.genome.jp/entry/K07936) GTP-binding nuclear protein Ran

[tnl:113491728](https://www.genome.jp/entry/tnl:113491728) [K14574](https://www.genome.jp/entry/K14574) ribosome maturation protein SBDS

[tnl:113493801](https://www.genome.jp/entry/tnl:113493801) [K11883](https://www.genome.jp/entry/K11883) RNA-binding protein NOB1

[tnl:113496312](https://www.genome.jp/entry/tnl:113496312) [K03097](https://www.genome.jp/entry/K03097) casein kinase II subunit alpha isoform X1

[tnl:113497609](https://www.genome.jp/entry/tnl:113497609) [K14566](https://www.genome.jp/entry/K14566) rRNA-processing protein FCF1 homolog

[tnl:113497767](https://www.genome.jp/entry/tnl:113497767) [K14290](https://www.genome.jp/entry/K14290) exportin-1

[tnl:113497867](https://www.genome.jp/entry/tnl:113497867) [K12845](https://www.genome.jp/entry/K12845) NHP2-like protein 1

[tnl:113498901](https://www.genome.jp/entry/tnl:113498901) [K11130](https://www.genome.jp/entry/K11130) H/ACA ribonucleoprotein complex subunit 3

[tnl:113500134](https://www.genome.jp/entry/tnl:113500134) [K14573](https://www.genome.jp/entry/K14573) RNA-binding protein 28

[tnl:113500257](https://www.genome.jp/entry/tnl:113500257) [K14569](https://www.genome.jp/entry/K14569) ribosome biogenesis protein BMS1 homolog

[tnl:113500981](https://www.genome.jp/entry/tnl:113500981) [K11128](https://www.genome.jp/entry/K11128) probable H/ACA ribonucleoprotein complex subunit 1

[tnl:113501719](https://www.genome.jp/entry/tnl:113501719) [K11108](https://www.genome.jp/entry/K11108) RNA 3'-terminal phosphate cyclase-like protein

[tnl:113501798](https://www.genome.jp/entry/tnl:113501798) [K18532](https://www.genome.jp/entry/K18532) adenylate kinase isoenzyme 6 homolog

[tnl:113501864](https://www.genome.jp/entry/tnl:113501864) [K03264](https://www.genome.jp/entry/K03264) eukaryotic translation initiation factor 6

[tnl:113502427](https://www.genome.jp/entry/tnl:113502427) [K14538](https://www.genome.jp/entry/K14538) guanine nucleotide-binding protein-like 3 homolog

[tnl:113504134](https://www.genome.jp/entry/tnl:113504134) [K06943](https://www.genome.jp/entry/K06943) nucleolar GTP-binding protein 1-like

[tnl:113504280](https://www.genome.jp/entry/tnl:113504280) [K06943](https://www.genome.jp/entry/K06943) nucleolar GTP-binding protein 1

[tnl:113505363](https://www.genome.jp/entry/tnl:113505363) [K13288](https://www.genome.jp/entry/K13288) probable oligoribonuclease

[tnl:113508231](https://www.genome.jp/entry/tnl:113508231) [K11131](https://www.genome.jp/entry/K11131) H/ACA ribonucleoprotein complex subunit 4-like

 [**tnl03040**](https://www.genome.jp/kegg-bin/show_pathway?166859291342340/tnl03040.args) **Spliceosome - Trichoplusia ni (cabbage looper) (**[**19**](javascript:display('tnl03040'))**)**

[tnl:113492055](https://www.genome.jp/entry/tnl:113492055) [K12834](https://www.genome.jp/entry/K12834) PHD finger-like domain-containing protein 5A

[tnl:113492916](https://www.genome.jp/entry/tnl:113492916) [K09564](https://www.genome.jp/entry/K09564) peptidyl-prolyl cis-trans isomerase E isoform X1

[tnl:113494550](https://www.genome.jp/entry/tnl:113494550) [K12812](https://www.genome.jp/entry/K12812) ATP-dependent RNA helicase WM6

[tnl:113494572](https://www.genome.jp/entry/tnl:113494572) [K12896](https://www.genome.jp/entry/K12896) RNA-binding protein 1-like isoform X1

[tnl:113495317](https://www.genome.jp/entry/tnl:113495317) [K12873](https://www.genome.jp/entry/K12873) protein BUD31 homolog

[tnl:113495560](https://www.genome.jp/entry/tnl:113495560) [K03283](https://www.genome.jp/entry/K03283) heat shock protein 68-like

[tnl:113496267](https://www.genome.jp/entry/tnl:113496267) [K03283](https://www.genome.jp/entry/K03283) heat shock protein 68-like

[tnl:113496527](https://www.genome.jp/entry/tnl:113496527) [K12823](https://www.genome.jp/entry/K12823) ATP-dependent RNA helicase dbp2-like isoform X1

[tnl:113497576](https://www.genome.jp/entry/tnl:113497576) [K12896](https://www.genome.jp/entry/K12896) serine/arginine-rich splicing factor 7-like

[tnl:113497748](https://www.genome.jp/entry/tnl:113497748) [K11094](https://www.genome.jp/entry/K11094) U1 small nuclear ribonucleoprotein A

[tnl:113497867](https://www.genome.jp/entry/tnl:113497867) [K12845](https://www.genome.jp/entry/K12845) NHP2-like protein 1

[tnl:113498637](https://www.genome.jp/entry/tnl:113498637) [K12874](https://www.genome.jp/entry/K12874) RNA helicase aquarius-like

[tnl:113499040](https://www.genome.jp/entry/tnl:113499040) [K12881](https://www.genome.jp/entry/K12881) THO complex subunit 4 isoform X1

[tnl:113504067](https://www.genome.jp/entry/tnl:113504067) [K12876](https://www.genome.jp/entry/K12876) RNA-binding protein 8A

[tnl:113505020](https://www.genome.jp/entry/tnl:113505020) [K12829](https://www.genome.jp/entry/K12829) splicing factor 3B subunit 2

[tnl:113506083](https://www.genome.jp/entry/tnl:113506083) [K13098](https://www.genome.jp/entry/K13098) RNA-binding protein cabeza isoform X1

[tnl:113506423](https://www.genome.jp/entry/tnl:113506423) [K03283](https://www.genome.jp/entry/K03283) heat shock 70 kDa protein cognate 4

[tnl:113506519](https://www.genome.jp/entry/tnl:113506519) [K12625](https://www.genome.jp/entry/K12625) U6 snRNA-associated Sm-like protein LSm6

[tnl:113507925](https://www.genome.jp/entry/tnl:113507925) [K12824](https://www.genome.jp/entry/K12824) transcription elongation regulator 1 isoform X1

 [**tnl00270**](https://www.genome.jp/kegg-bin/show_pathway?166859291342340/tnl00270.args) **Cysteine and methionine metabolism - Trichoplusia ni (cabbage looper) (**[**18**](javascript:display('tnl00270'))**)**

[tnl:113492726](https://www.genome.jp/entry/tnl:113492726) [K11204](https://www.genome.jp/entry/K11204) glutamate--cysteine ligase catalytic subunit

[tnl:113492937](https://www.genome.jp/entry/tnl:113492937) [K14454](https://www.genome.jp/entry/K14454) aspartate aminotransferase, cytoplasmic

[tnl:113493478](https://www.genome.jp/entry/tnl:113493478) [K01251](https://www.genome.jp/entry/K01251) adenosylhomocysteinase

[tnl:113494198](https://www.genome.jp/entry/tnl:113494198) [K11205](https://www.genome.jp/entry/K11205) glutamate--cysteine ligase regulatory subunit isoform X1

[tnl:113494305](https://www.genome.jp/entry/tnl:113494305) [K21456](https://www.genome.jp/entry/K21456) glutathione synthetase-like isoform X1

[tnl:113494548](https://www.genome.jp/entry/tnl:113494548) [K00816](https://www.genome.jp/entry/K00816) kynurenine--oxoglutarate transaminase 3 isoform X1

[tnl:113496177](https://www.genome.jp/entry/tnl:113496177) [K00058](https://www.genome.jp/entry/K00058) D-3-phosphoglycerate dehydrogenase

[tnl:113497424](https://www.genome.jp/entry/tnl:113497424) [K00797](https://www.genome.jp/entry/K00797) spermidine synthase

[tnl:113497832](https://www.genome.jp/entry/tnl:113497832) [K00025](https://www.genome.jp/entry/K00025) malate dehydrogenase, cytoplasmic

[tnl:113499273](https://www.genome.jp/entry/tnl:113499273) [K00772](https://www.genome.jp/entry/K00772) S-methyl-5'-thioadenosine phosphorylase-like

[tnl:113500574](https://www.genome.jp/entry/tnl:113500574) [K00831](https://www.genome.jp/entry/K00831) probable phosphoserine aminotransferase

[tnl:113500626](https://www.genome.jp/entry/tnl:113500626) [K08963](https://www.genome.jp/entry/K08963) methylthioribose-1-phosphate isomerase

[tnl:113500928](https://www.genome.jp/entry/tnl:113500928) [K00789](https://www.genome.jp/entry/K00789) S-adenosylmethionine synthase isoform X1

[tnl:113501183](https://www.genome.jp/entry/tnl:113501183) [K00547](https://www.genome.jp/entry/K00547) uncharacterized protein LOC113501183

[tnl:113502164](https://www.genome.jp/entry/tnl:113502164) [K01758](https://www.genome.jp/entry/K01758) cystathionine gamma-lyase-like

[tnl:113502268](https://www.genome.jp/entry/tnl:113502268) [K00802](https://www.genome.jp/entry/K00802) spermine synthase

[tnl:113504890](https://www.genome.jp/entry/tnl:113504890) [K01697](https://www.genome.jp/entry/K01697) cystathionine beta-synthase-like

[tnl:113506948](https://www.genome.jp/entry/tnl:113506948) [K00016](https://www.genome.jp/entry/K00016) L-lactate dehydrogenase-like isoform X1

 [**tnl00520**](https://www.genome.jp/kegg-bin/show_pathway?166859291342340/tnl00520.args) **Amino sugar and nucleotide sugar metabolism - Trichoplusia ni (cabbage looper) (**[**17**](javascript:display('tnl00520'))**)**

[tnl:113493566](https://www.genome.jp/entry/tnl:113493566) [K08678](https://www.genome.jp/entry/K08678) UDP-glucuronic acid decarboxylase 1

[tnl:113494165](https://www.genome.jp/entry/tnl:113494165) [K00966](https://www.genome.jp/entry/K00966) mannose-1-phosphate guanyltransferase alpha-A

[tnl:113494316](https://www.genome.jp/entry/tnl:113494316) [K01810](https://www.genome.jp/entry/K01810) glucose-6-phosphate isomerase

[tnl:113495499](https://www.genome.jp/entry/tnl:113495499) [K01836](https://www.genome.jp/entry/K01836) phosphoacetylglucosamine mutase

[tnl:113496554](https://www.genome.jp/entry/tnl:113496554) [K02377](https://www.genome.jp/entry/K02377) GDP-L-fucose synthase isoform X1

[tnl:113497289](https://www.genome.jp/entry/tnl:113497289) [K12373](https://www.genome.jp/entry/K12373) chitooligosaccharidolytic beta-N-acetylglucosaminidase

[tnl:113497947](https://www.genome.jp/entry/tnl:113497947) [K00844](https://www.genome.jp/entry/K00844) hexokinase type 2 isoform X1

[tnl:113498601](https://www.genome.jp/entry/tnl:113498601) [K12373](https://www.genome.jp/entry/K12373) beta-hexosaminidase subunit beta-like isoform X1

[tnl:113500327](https://www.genome.jp/entry/tnl:113500327) [K02564](https://www.genome.jp/entry/K02564) glucosamine-6-phosphate isomerase isoform X1

[tnl:113501660](https://www.genome.jp/entry/tnl:113501660) [K00963](https://www.genome.jp/entry/K00963) UTP--glucose-1-phosphate uridylyltransferase isoform X1

[tnl:113503659](https://www.genome.jp/entry/tnl:113503659) [K01835](https://www.genome.jp/entry/K01835) phosphoglucomutase

[tnl:113503711](https://www.genome.jp/entry/tnl:113503711) [K01784](https://www.genome.jp/entry/K01784) UDP-glucose 4-epimerase-like

[tnl:113503730](https://www.genome.jp/entry/tnl:113503730) [K01711](https://www.genome.jp/entry/K01711) GDP-mannose 4,6 dehydratase

[tnl:113505855](https://www.genome.jp/entry/tnl:113505855) [K01809](https://www.genome.jp/entry/K01809) mannose-6-phosphate isomerase

[tnl:113507917](https://www.genome.jp/entry/tnl:113507917) [K00012](https://www.genome.jp/entry/K00012) UDP-glucose 6-dehydrogenase

[tnl:113508098](https://www.genome.jp/entry/tnl:113508098) [K17497](https://www.genome.jp/entry/K17497) phosphomannomutase 2

[tnl:113508463](https://www.genome.jp/entry/tnl:113508463) [K00326](https://www.genome.jp/entry/K00326) NADH-cytochrome b5 reductase 3 isoform X1

 [**tnl00983**](https://www.genome.jp/kegg-bin/show_pathway?166859291342340/tnl00983.args) **Drug metabolism - other enzymes - Trichoplusia ni (cabbage looper) (**[**17**](javascript:display('tnl00983'))**)**

[tnl:113492544](https://www.genome.jp/entry/tnl:113492544) [K00940](https://www.genome.jp/entry/K00940) nucleoside diphosphate kinase

[tnl:113494590](https://www.genome.jp/entry/tnl:113494590) [K00876](https://www.genome.jp/entry/K00876) probable uridine-cytidine kinase isoform X1

[tnl:113494907](https://www.genome.jp/entry/tnl:113494907) [K00799](https://www.genome.jp/entry/K00799) glutathione S-transferase 1-like

[tnl:113496347](https://www.genome.jp/entry/tnl:113496347) [K10808](https://www.genome.jp/entry/K10808) ribonucleoside-diphosphate reductase subunit M2

[tnl:113496731](https://www.genome.jp/entry/tnl:113496731) [K00799](https://www.genome.jp/entry/K00799) uncharacterized protein LOC113496731

[tnl:113497008](https://www.genome.jp/entry/tnl:113497008) [K00799](https://www.genome.jp/entry/K00799) glutathione S-transferase 1-1-like

[tnl:113497009](https://www.genome.jp/entry/tnl:113497009) [K00799](https://www.genome.jp/entry/K00799) glutathione S-transferase 1-1-like isoform X1

[tnl:113497121](https://www.genome.jp/entry/tnl:113497121) [K10807](https://www.genome.jp/entry/K10807) ribonucleoside-diphosphate reductase large subunit

[tnl:113497732](https://www.genome.jp/entry/tnl:113497732) [K00106](https://www.genome.jp/entry/K00106) xanthine dehydrogenase

[tnl:113499351](https://www.genome.jp/entry/tnl:113499351) [K01951](https://www.genome.jp/entry/K01951) GMP synthase [glutamine-hydrolyzing] isoform X1

[tnl:113499552](https://www.genome.jp/entry/tnl:113499552) [K00799](https://www.genome.jp/entry/K00799) uncharacterized protein LOC113499552

[tnl:113499695](https://www.genome.jp/entry/tnl:113499695) [K01520](https://www.genome.jp/entry/K01520) deoxyuridine 5'-triphosphate nucleotidohydrolase

[tnl:113501491](https://www.genome.jp/entry/tnl:113501491) [K01519](https://www.genome.jp/entry/K01519) inosine triphosphate pyrophosphatase

[tnl:113505327](https://www.genome.jp/entry/tnl:113505327) [K13800](https://www.genome.jp/entry/K13800) UMP-CMP kinase

[tnl:113507103](https://www.genome.jp/entry/tnl:113507103) [K00799](https://www.genome.jp/entry/K00799) glutathione S-transferase 1-1-like

[tnl:113508050](https://www.genome.jp/entry/tnl:113508050) [K00799](https://www.genome.jp/entry/K00799) glutathione S-transferase 1-1-like

[tnl:113508532](https://www.genome.jp/entry/tnl:113508532) [K00088](https://www.genome.jp/entry/K00088) inosine-5'-monophosphate dehydrogenase

**** [**tnl04214**](https://www.genome.jp/kegg-bin/show_pathway?166859291342340/tnl04214.args) **Apoptosis - fly - Trichoplusia ni (cabbage looper) (**[**16**](javascript:display('tnl04214'))**)**

[tnl:113491757](https://www.genome.jp/entry/tnl:113491757) [K04371](https://www.genome.jp/entry/K04371) mitogen-activated protein kinase ERK-A

[tnl:113492628](https://www.genome.jp/entry/tnl:113492628) [K08738](https://www.genome.jp/entry/K08738) cytochrome c

[tnl:113493668](https://www.genome.jp/entry/tnl:113493668) [K04365](https://www.genome.jp/entry/K04365) raf homolog serine/threonine-protein kinase Raf isoform X1

[tnl:113493805](https://www.genome.jp/entry/tnl:113493805) [K07827](https://www.genome.jp/entry/K07827) GTPase HRas

[tnl:113494585](https://www.genome.jp/entry/tnl:113494585) [K04431](https://www.genome.jp/entry/K04431) dual specificity mitogen-activated protein kinase kinase 7-like isoform X1

[tnl:113494876](https://www.genome.jp/entry/tnl:113494876) [K01527](https://www.genome.jp/entry/K01527) transcription factor BTF3 homolog 4-like

[tnl:113495776](https://www.genome.jp/entry/tnl:113495776) [K03262](https://www.genome.jp/entry/K03262) eukaryotic translation initiation factor 5 isoform X1

[tnl:113497314](https://www.genome.jp/entry/tnl:113497314) [K04404](https://www.genome.jp/entry/K04404) uncharacterized protein LOC113497314 isoform X1

[tnl:113500953](https://www.genome.jp/entry/tnl:113500953) [K04412](https://www.genome.jp/entry/K04412) serine/threonine-protein kinase 4 isoform X1

[tnl:113501742](https://www.genome.jp/entry/tnl:113501742) [K07611](https://www.genome.jp/entry/K07611) lamin-C-like

[tnl:113503670](https://www.genome.jp/entry/tnl:113503670) [K04440](https://www.genome.jp/entry/K04440) stress-activated protein kinase JNK isoform X1

[tnl:113504482](https://www.genome.jp/entry/tnl:113504482) [K20009](https://www.genome.jp/entry/K20009) caspase Dronc

[tnl:113504517](https://www.genome.jp/entry/tnl:113504517) [K04397](https://www.genome.jp/entry/K04397) caspase-1-like

[tnl:113504518](https://www.genome.jp/entry/tnl:113504518) [K04397](https://www.genome.jp/entry/K04397) caspase-1

[tnl:113508433](https://www.genome.jp/entry/tnl:113508433) [K17065](https://www.genome.jp/entry/K17065) dynamin-1-like protein isoform X1

[tnl:113508819](https://www.genome.jp/entry/tnl:113508819) [K09848](https://www.genome.jp/entry/K09848) TNF receptor-associated factor 4 isoform X1

 [**tnl04070**](https://www.genome.jp/kegg-bin/show_pathway?166859291342340/tnl04070.args) **Phosphatidylinositol signaling system - Trichoplusia ni (cabbage looper) (**[**16**](javascript:display('tnl04070'))**)**

[tnl:113491732](https://www.genome.jp/entry/tnl:113491732) [K13024](https://www.genome.jp/entry/K13024) inositol hexakisphosphate and diphosphoinositol-pentakisphosphate kinase isoform X1

[tnl:113492561](https://www.genome.jp/entry/tnl:113492561) [K18081](https://www.genome.jp/entry/K18081) myotubularin-related protein 2

[tnl:113493839](https://www.genome.jp/entry/tnl:113493839) [K00920](https://www.genome.jp/entry/K00920) phosphatidylinositol 5-phosphate 4-kinase type-2 alpha isoform X1

[tnl:113494878](https://www.genome.jp/entry/tnl:113494878) [K21798](https://www.genome.jp/entry/K21798) phosphatidylinositide phosphatase SAC2-like isoform X1

[tnl:113497125](https://www.genome.jp/entry/tnl:113497125) [K05858](https://www.genome.jp/entry/K05858) 1-phosphatidylinositol 4,5-bisphosphate phosphodiesterase classes I and II

[tnl:113497272](https://www.genome.jp/entry/tnl:113497272) [K20279](https://www.genome.jp/entry/K20279) synaptojanin-1

[tnl:113497664](https://www.genome.jp/entry/tnl:113497664) [K01110](https://www.genome.jp/entry/K01110) phosphatidylinositol 3,4,5-trisphosphate 3-phosphatase and dual-specificity protein phosphatase PTEN

[tnl:113498047](https://www.genome.jp/entry/tnl:113498047) [K15759](https://www.genome.jp/entry/K15759) putative inositol monophosphatase 3

[tnl:113498973](https://www.genome.jp/entry/tnl:113498973) [K01106](https://www.genome.jp/entry/K01106) uncharacterized protein LOC113498973 isoform X1

[tnl:113499295](https://www.genome.jp/entry/tnl:113499295) [K13711](https://www.genome.jp/entry/K13711) phosphatidylinositol 4-kinase type 2-alpha-like isoform X1

[tnl:113501180](https://www.genome.jp/entry/tnl:113501180) [K01099](https://www.genome.jp/entry/K01099) type II inositol 1,4,5-trisphosphate 5-phosphatase

[tnl:113503987](https://www.genome.jp/entry/tnl:113503987) [K05858](https://www.genome.jp/entry/K05858) 1-phosphatidylinositol 4,5-bisphosphate phosphodiesterase isoform X1

[tnl:113504269](https://www.genome.jp/entry/tnl:113504269) [K00889](https://www.genome.jp/entry/K00889) phosphatidylinositol 4-phosphate 5-kinase type-1 alpha-like isoform X1

[tnl:113505041](https://www.genome.jp/entry/tnl:113505041) [K01116](https://www.genome.jp/entry/K01116) 1-phosphatidylinositol 4,5-bisphosphate phosphodiesterase gamma-1

[tnl:113505800](https://www.genome.jp/entry/tnl:113505800) [K02183](https://www.genome.jp/entry/K02183) calmodulin isoform X1

[tnl:113507614](https://www.genome.jp/entry/tnl:113507614) [K00923](https://www.genome.jp/entry/K00923) phosphatidylinositol 4-phosphate 3-kinase C2 domain-containing subunit alpha isoform X1

**** [**tnl04350**](https://www.genome.jp/kegg-bin/show_pathway?166859291342340/tnl04350.args) **TGF-beta signaling pathway - Trichoplusia ni (cabbage looper) (**[**15**](javascript:display('tnl04350'))**)**

[tnl:113491757](https://www.genome.jp/entry/tnl:113491757) [K04371](https://www.genome.jp/entry/K04371) mitogen-activated protein kinase ERK-A

[tnl:113491887](https://www.genome.jp/entry/tnl:113491887) [K04678](https://www.genome.jp/entry/K04678) E3 ubiquitin-protein ligase SMURF2

[tnl:113493196](https://www.genome.jp/entry/tnl:113493196) [K04514](https://www.genome.jp/entry/K04514) rho-associated protein kinase 2

[tnl:113493430](https://www.genome.jp/entry/tnl:113493430) [K13596](https://www.genome.jp/entry/K13596) activin receptor type-2A-like

[tnl:113494449](https://www.genome.jp/entry/tnl:113494449) [K04675](https://www.genome.jp/entry/K04675) activin receptor type-1 isoform X1

[tnl:113494680](https://www.genome.jp/entry/tnl:113494680) [K03456](https://www.genome.jp/entry/K03456) serine/threonine-protein phosphatase PP2A 65 kDa regulatory subunit

[tnl:113497312](https://www.genome.jp/entry/tnl:113497312) [K04676](https://www.genome.jp/entry/K04676) protein mothers against dpp

[tnl:113499255](https://www.genome.jp/entry/tnl:113499255) [K04501](https://www.genome.jp/entry/K04501) mothers against decapentaplegic homolog 4 isoform X1

[tnl:113499516](https://www.genome.jp/entry/tnl:113499516) [K04382](https://www.genome.jp/entry/K04382) serine/threonine-protein phosphatase 2A catalytic subunit beta isoform

[tnl:113503427](https://www.genome.jp/entry/tnl:113503427) [K04513](https://www.genome.jp/entry/K04513) ras-like GTP-binding protein Rho1 isoform X1

[tnl:113503669](https://www.genome.jp/entry/tnl:113503669) [K13578](https://www.genome.jp/entry/K13578) bone morphogenetic protein receptor type-1B isoform X1

[tnl:113504177](https://www.genome.jp/entry/tnl:113504177) [K06766](https://www.genome.jp/entry/K06766) neogenin-like

[tnl:113504422](https://www.genome.jp/entry/tnl:113504422) [K04688](https://www.genome.jp/entry/K04688) ribosomal protein S6 kinase beta-2

[tnl:113505189](https://www.genome.jp/entry/tnl:113505189) [K04674](https://www.genome.jp/entry/K04674) TGF-beta receptor type-1-like isoform X1

[tnl:113508299](https://www.genome.jp/entry/tnl:113508299) [K03094](https://www.genome.jp/entry/K03094) S-phase kinase-associated protein 1

 [**tnl00010**](https://www.genome.jp/kegg-bin/show_pathway?166859291342340/tnl00010.args) **Glycolysis / Gluconeogenesis - Trichoplusia ni (cabbage looper) (**[**14**](javascript:display('tnl00010'))**)**

[tnl:113493924](https://www.genome.jp/entry/tnl:113493924) [K01803](https://www.genome.jp/entry/K01803) triosephosphate isomerase

[tnl:113494316](https://www.genome.jp/entry/tnl:113494316) [K01810](https://www.genome.jp/entry/K01810) glucose-6-phosphate isomerase

[tnl:113494495](https://www.genome.jp/entry/tnl:113494495) [K00927](https://www.genome.jp/entry/K00927) phosphoglycerate kinase

[tnl:113496310](https://www.genome.jp/entry/tnl:113496310) [K00850](https://www.genome.jp/entry/K00850) ATP-dependent 6-phosphofructokinase isoform X1

[tnl:113497863](https://www.genome.jp/entry/tnl:113497863) [K00121](https://www.genome.jp/entry/K00121) alcohol dehydrogenase class-3

[tnl:113497947](https://www.genome.jp/entry/tnl:113497947) [K00844](https://www.genome.jp/entry/K00844) hexokinase type 2 isoform X1

[tnl:113499004](https://www.genome.jp/entry/tnl:113499004) [K01689](https://www.genome.jp/entry/K01689) enolase

[tnl:113500423](https://www.genome.jp/entry/tnl:113500423) [K00873](https://www.genome.jp/entry/K00873) pyruvate kinase-like isoform X1

[tnl:113500921](https://www.genome.jp/entry/tnl:113500921) [K00128](https://www.genome.jp/entry/K00128) retinal dehydrogenase 1-like

[tnl:113503573](https://www.genome.jp/entry/tnl:113503573) [K00128](https://www.genome.jp/entry/K00128) aldehyde dehydrogenase, mitochondrial

[tnl:113503659](https://www.genome.jp/entry/tnl:113503659) [K01835](https://www.genome.jp/entry/K01835) phosphoglucomutase

[tnl:113506529](https://www.genome.jp/entry/tnl:113506529) [K01834](https://www.genome.jp/entry/K01834) phosphoglycerate mutase 2-like

[tnl:113506948](https://www.genome.jp/entry/tnl:113506948) [K00016](https://www.genome.jp/entry/K00016) L-lactate dehydrogenase-like isoform X1

[tnl:113509001](https://www.genome.jp/entry/tnl:113509001) [K00134](https://www.genome.jp/entry/K00134) glyceraldehyde-3-phosphate dehydrogenase

**** [**tnl04624**](https://www.genome.jp/kegg-bin/show_pathway?166859291342340/tnl04624.args) **Toll and Imd signaling pathway - Trichoplusia ni (cabbage looper) (**[**14**](javascript:display('tnl04624'))**)**

[tnl:113491662](https://www.genome.jp/entry/tnl:113491662) [K18809](https://www.genome.jp/entry/K18809) protein toll-like

[tnl:113494585](https://www.genome.jp/entry/tnl:113494585) [K04431](https://www.genome.jp/entry/K04431) dual specificity mitogen-activated protein kinase kinase 7-like isoform X1

[tnl:113497314](https://www.genome.jp/entry/tnl:113497314) [K04404](https://www.genome.jp/entry/K04404) uncharacterized protein LOC113497314 isoform X1

[tnl:113497745](https://www.genome.jp/entry/tnl:113497745) [K06689](https://www.genome.jp/entry/K06689) ubiquitin-conjugating enzyme E2-17 kDa

[tnl:113497879](https://www.genome.jp/entry/tnl:113497879) [K10380](https://www.genome.jp/entry/K10380) ankyrin-1-like isoform X1

[tnl:113498326](https://www.genome.jp/entry/tnl:113498326) [K20692](https://www.genome.jp/entry/K20692) beta-1,3-glucan-binding protein-like

[tnl:113500756](https://www.genome.jp/entry/tnl:113500756) [K04733](https://www.genome.jp/entry/K04733) interleukin-1 receptor-associated kinase 4-like

[tnl:113501535](https://www.genome.jp/entry/tnl:113501535) [K10704](https://www.genome.jp/entry/K10704) ubiquitin-conjugating enzyme E2 variant 2

[tnl:113501882](https://www.genome.jp/entry/tnl:113501882) [K20699](https://www.genome.jp/entry/K20699) protein immune deficiency

[tnl:113501942](https://www.genome.jp/entry/tnl:113501942) [K04441](https://www.genome.jp/entry/K04441) mitogen-activated protein kinase p38b-like isoform X1

[tnl:113502933](https://www.genome.jp/entry/tnl:113502933) [K01446](https://www.genome.jp/entry/K01446) peptidoglycan-recognition protein LB-like

[tnl:113502954](https://www.genome.jp/entry/tnl:113502954) [K20703](https://www.genome.jp/entry/K20703) FAS-associated factor 1 isoform X1

[tnl:113503670](https://www.genome.jp/entry/tnl:113503670) [K04440](https://www.genome.jp/entry/K04440) stress-activated protein kinase JNK isoform X1

[tnl:113504445](https://www.genome.jp/entry/tnl:113504445) [K04729](https://www.genome.jp/entry/K04729) myeloid differentiation primary response protein MyD88-like

 [**tnl00190**](https://www.genome.jp/kegg-bin/show_pathway?166859291342340/tnl00190.args) **Oxidative phosphorylation - Trichoplusia ni (cabbage looper) (**[**14**](javascript:display('tnl00190'))**)**

[tnl:113492628](https://www.genome.jp/entry/tnl:113492628) [K08738](https://www.genome.jp/entry/K08738) cytochrome c

[tnl:113492751](https://www.genome.jp/entry/tnl:113492751) [K02144](https://www.genome.jp/entry/K02144) V-type proton ATPase subunit H isoform X1

[tnl:113493473](https://www.genome.jp/entry/tnl:113493473) [K02146](https://www.genome.jp/entry/K02146) V-type proton ATPase subunit d

[tnl:113494681](https://www.genome.jp/entry/tnl:113494681) [K02147](https://www.genome.jp/entry/K02147) V-type proton ATPase subunit B

[tnl:113496116](https://www.genome.jp/entry/tnl:113496116) [K02145](https://www.genome.jp/entry/K02145) V-type proton ATPase catalytic subunit A

[tnl:113496232](https://www.genome.jp/entry/tnl:113496232) [K02149](https://www.genome.jp/entry/K02149) V-type proton ATPase subunit D

[tnl:113499397](https://www.genome.jp/entry/tnl:113499397) [K02150](https://www.genome.jp/entry/K02150) V-type proton ATPase subunit E

[tnl:113500459](https://www.genome.jp/entry/tnl:113500459) [K11726](https://www.genome.jp/entry/K11726) inorganic pyrophosphatase

[tnl:113500508](https://www.genome.jp/entry/tnl:113500508) [K02151](https://www.genome.jp/entry/K02151) V-type proton ATPase subunit F

[tnl:113500605](https://www.genome.jp/entry/tnl:113500605) [K02154](https://www.genome.jp/entry/K02154) V-type proton ATPase 116 kDa subunit a

[tnl:113502333](https://www.genome.jp/entry/tnl:113502333) [K03661](https://www.genome.jp/entry/K03661) V-type proton ATPase 21 kDa proteolipid subunit

[tnl:113503154](https://www.genome.jp/entry/tnl:113503154) [K02148](https://www.genome.jp/entry/K02148) V-type proton ATPase subunit C

[tnl:113505484](https://www.genome.jp/entry/tnl:113505484) [K03662](https://www.genome.jp/entry/K03662) V-type proton ATPase subunit S1

[tnl:113506207](https://www.genome.jp/entry/tnl:113506207) [K02152](https://www.genome.jp/entry/K02152) V-type proton ATPase subunit G

 [**tnl00620**](https://www.genome.jp/kegg-bin/show_pathway?166859291342340/tnl00620.args) **Pyruvate metabolism - Trichoplusia ni (cabbage looper) (**[**14**](javascript:display('tnl00620'))**)**

[tnl:113492630](https://www.genome.jp/entry/tnl:113492630) [K00029](https://www.genome.jp/entry/K00029) LOW QUALITY PROTEIN: NADP-dependent malic enzyme-like

[tnl:113494704](https://www.genome.jp/entry/tnl:113494704) [K01069](https://www.genome.jp/entry/K01069) hydroxyacylglutathione hydrolase, mitochondrial isoform X1

[tnl:113495603](https://www.genome.jp/entry/tnl:113495603) [K01679](https://www.genome.jp/entry/K01679) fumarate hydratase, mitochondrial-like isoform X1

[tnl:113497192](https://www.genome.jp/entry/tnl:113497192) [K00049](https://www.genome.jp/entry/K00049) glyoxylate reductase/hydroxypyruvate reductase-like

[tnl:113497832](https://www.genome.jp/entry/tnl:113497832) [K00025](https://www.genome.jp/entry/K00025) malate dehydrogenase, cytoplasmic

[tnl:113497863](https://www.genome.jp/entry/tnl:113497863) [K00121](https://www.genome.jp/entry/K00121) alcohol dehydrogenase class-3

[tnl:113498020](https://www.genome.jp/entry/tnl:113498020) [K00626](https://www.genome.jp/entry/K00626) acetyl-CoA acetyltransferase, mitochondrial

[tnl:113498563](https://www.genome.jp/entry/tnl:113498563) [K01512](https://www.genome.jp/entry/K01512) acylphosphatase-1-like

[tnl:113498903](https://www.genome.jp/entry/tnl:113498903) [K11262](https://www.genome.jp/entry/K11262) acetyl-CoA carboxylase isoform X1

[tnl:113500423](https://www.genome.jp/entry/tnl:113500423) [K00873](https://www.genome.jp/entry/K00873) pyruvate kinase-like isoform X1

[tnl:113500921](https://www.genome.jp/entry/tnl:113500921) [K00128](https://www.genome.jp/entry/K00128) retinal dehydrogenase 1-like

[tnl:113503573](https://www.genome.jp/entry/tnl:113503573) [K00128](https://www.genome.jp/entry/K00128) aldehyde dehydrogenase, mitochondrial

[tnl:113506948](https://www.genome.jp/entry/tnl:113506948) [K00016](https://www.genome.jp/entry/K00016) L-lactate dehydrogenase-like isoform X1

[tnl:113507140](https://www.genome.jp/entry/tnl:113507140) [K00049](https://www.genome.jp/entry/K00049) LOW QUALITY PROTEIN: glyoxylate reductase/hydroxypyruvate reductase-like

 [**tnl00240**](https://www.genome.jp/kegg-bin/show_pathway?166859291342340/tnl00240.args) **Pyrimidine metabolism - Trichoplusia ni (cabbage looper) (**[**14**](javascript:display('tnl00240'))**)**

[tnl:113492544](https://www.genome.jp/entry/tnl:113492544) [K00940](https://www.genome.jp/entry/K00940) nucleoside diphosphate kinase

[tnl:113494590](https://www.genome.jp/entry/tnl:113494590) [K00876](https://www.genome.jp/entry/K00876) probable uridine-cytidine kinase isoform X1

[tnl:113494755](https://www.genome.jp/entry/tnl:113494755) [K12304](https://www.genome.jp/entry/K12304) soluble calcium-activated nucleotidase 1

[tnl:113496347](https://www.genome.jp/entry/tnl:113496347) [K10808](https://www.genome.jp/entry/K10808) ribonucleoside-diphosphate reductase subunit M2

[tnl:113497121](https://www.genome.jp/entry/tnl:113497121) [K10807](https://www.genome.jp/entry/K10807) ribonucleoside-diphosphate reductase large subunit

[tnl:113497953](https://www.genome.jp/entry/tnl:113497953) [K01081](https://www.genome.jp/entry/K01081) cytosolic purine 5'-nucleotidase isoform X1

[tnl:113499695](https://www.genome.jp/entry/tnl:113499695) [K01520](https://www.genome.jp/entry/K01520) deoxyuridine 5'-triphosphate nucleotidohydrolase

[tnl:113501642](https://www.genome.jp/entry/tnl:113501642) [K07023](https://www.genome.jp/entry/K07023) HD domain-containing protein 2

[tnl:113504558](https://www.genome.jp/entry/tnl:113504558) [K11540](https://www.genome.jp/entry/K11540) CAD protein isoform X1

[tnl:113505248](https://www.genome.jp/entry/tnl:113505248) [K01518](https://www.genome.jp/entry/K01518) bis(5'-nucleosyl)-tetraphosphatase [asymmetrical]

[tnl:113505327](https://www.genome.jp/entry/tnl:113505327) [K13800](https://www.genome.jp/entry/K13800) UMP-CMP kinase

[tnl:113507725](https://www.genome.jp/entry/tnl:113507725) [K01937](https://www.genome.jp/entry/K01937) CTP synthase

[tnl:113508501](https://www.genome.jp/entry/tnl:113508501) [K00560](https://www.genome.jp/entry/K00560) thymidylate synthase

[tnl:113508579](https://www.genome.jp/entry/tnl:113508579) [K24242](https://www.genome.jp/entry/K24242) 7-methylguanosine phosphate-specific 5'-nucleotidase

 [**tnl02010**](https://www.genome.jp/kegg-bin/show_pathway?166859291342340/tnl02010.args) **ABC transporters - Trichoplusia ni (cabbage looper) (**[**14**](javascript:display('tnl02010'))**)**

[tnl:113492148](https://www.genome.jp/entry/tnl:113492148) [K05643](https://www.genome.jp/entry/K05643) ATP-binding cassette sub-family A member 1

[tnl:113493522](https://www.genome.jp/entry/tnl:113493522) [K05673](https://www.genome.jp/entry/K05673) probable multidrug resistance-associated protein lethal(2)03659

[tnl:113493562](https://www.genome.jp/entry/tnl:113493562) [K05673](https://www.genome.jp/entry/K05673) multidrug resistance-associated protein 4-like

[tnl:113494000](https://www.genome.jp/entry/tnl:113494000) [K05658](https://www.genome.jp/entry/K05658) multidrug resistance protein homolog 49-like isoform X1

[tnl:113497134](https://www.genome.jp/entry/tnl:113497134) [K05680](https://www.genome.jp/entry/K05680) ATP-binding cassette sub-family G member 1

[tnl:113497233](https://www.genome.jp/entry/tnl:113497233) [K05679](https://www.genome.jp/entry/K05679) ATP-binding cassette sub-family G member 4 isoform X1

[tnl:113497341](https://www.genome.jp/entry/tnl:113497341) [K05665](https://www.genome.jp/entry/K05665) multidrug resistance-associated protein 1

[tnl:113497342](https://www.genome.jp/entry/tnl:113497342) [K05665](https://www.genome.jp/entry/K05665) multidrug resistance-associated protein 1-like isoform X1

[tnl:113498587](https://www.genome.jp/entry/tnl:113498587) [K05673](https://www.genome.jp/entry/K05673) probable multidrug resistance-associated protein lethal(2)03659 isoform X1

[tnl:113499081](https://www.genome.jp/entry/tnl:113499081) [K05661](https://www.genome.jp/entry/K05661) ATP-binding cassette sub-family B member 6, mitochondrial

[tnl:113501075](https://www.genome.jp/entry/tnl:113501075) [K05673](https://www.genome.jp/entry/K05673) multidrug resistance-associated protein 4-like

[tnl:113504461](https://www.genome.jp/entry/tnl:113504461) [K05033](https://www.genome.jp/entry/K05033) ATP-binding cassette sub-family C member Sur

[tnl:113505221](https://www.genome.jp/entry/tnl:113505221) [K05643](https://www.genome.jp/entry/K05643) retinal-specific ATP-binding cassette transporter-like isoform X1

[tnl:113507463](https://www.genome.jp/entry/tnl:113507463) [K05658](https://www.genome.jp/entry/K05658) multidrug resistance protein homolog 49-like

**** [**tnl00562**](https://www.genome.jp/kegg-bin/show_pathway?166859291342340/tnl00562.args) **Inositol phosphate metabolism - Trichoplusia ni (cabbage looper) (**[**14**](javascript:display('tnl00562'))**)**

[tnl:113492561](https://www.genome.jp/entry/tnl:113492561) [K18081](https://www.genome.jp/entry/K18081) myotubularin-related protein 2

[tnl:113493839](https://www.genome.jp/entry/tnl:113493839) [K00920](https://www.genome.jp/entry/K00920) phosphatidylinositol 5-phosphate 4-kinase type-2 alpha isoform X1

[tnl:113493924](https://www.genome.jp/entry/tnl:113493924) [K01803](https://www.genome.jp/entry/K01803) triosephosphate isomerase

[tnl:113494878](https://www.genome.jp/entry/tnl:113494878) [K21798](https://www.genome.jp/entry/K21798) phosphatidylinositide phosphatase SAC2-like isoform X1

[tnl:113497125](https://www.genome.jp/entry/tnl:113497125) [K05858](https://www.genome.jp/entry/K05858) 1-phosphatidylinositol 4,5-bisphosphate phosphodiesterase classes I and II

[tnl:113497272](https://www.genome.jp/entry/tnl:113497272) [K20279](https://www.genome.jp/entry/K20279) synaptojanin-1

[tnl:113497664](https://www.genome.jp/entry/tnl:113497664) [K01110](https://www.genome.jp/entry/K01110) phosphatidylinositol 3,4,5-trisphosphate 3-phosphatase and dual-specificity protein phosphatase PTEN

[tnl:113498973](https://www.genome.jp/entry/tnl:113498973) [K01106](https://www.genome.jp/entry/K01106) uncharacterized protein LOC113498973 isoform X1

[tnl:113499295](https://www.genome.jp/entry/tnl:113499295) [K13711](https://www.genome.jp/entry/K13711) phosphatidylinositol 4-kinase type 2-alpha-like isoform X1

[tnl:113501180](https://www.genome.jp/entry/tnl:113501180) [K01099](https://www.genome.jp/entry/K01099) type II inositol 1,4,5-trisphosphate 5-phosphatase

[tnl:113503987](https://www.genome.jp/entry/tnl:113503987) [K05858](https://www.genome.jp/entry/K05858) 1-phosphatidylinositol 4,5-bisphosphate phosphodiesterase isoform X1

[tnl:113504269](https://www.genome.jp/entry/tnl:113504269) [K00889](https://www.genome.jp/entry/K00889) phosphatidylinositol 4-phosphate 5-kinase type-1 alpha-like isoform X1

[tnl:113505041](https://www.genome.jp/entry/tnl:113505041) [K01116](https://www.genome.jp/entry/K01116) 1-phosphatidylinositol 4,5-bisphosphate phosphodiesterase gamma-1

[tnl:113507614](https://www.genome.jp/entry/tnl:113507614) [K00923](https://www.genome.jp/entry/K00923) phosphatidylinositol 4-phosphate 3-kinase C2 domain-containing subunit alpha isoform X1

**** [**tnl01250**](https://www.genome.jp/kegg-bin/show_pathway?166859291342340/tnl01250.args) **Biosynthesis of nucleotide sugars - Trichoplusia ni (cabbage looper) (**[**13**](javascript:display('tnl01250'))**)**

[tnl:113493566](https://www.genome.jp/entry/tnl:113493566) [K08678](https://www.genome.jp/entry/K08678) UDP-glucuronic acid decarboxylase 1

[tnl:113494165](https://www.genome.jp/entry/tnl:113494165) [K00966](https://www.genome.jp/entry/K00966) mannose-1-phosphate guanyltransferase alpha-A

[tnl:113494316](https://www.genome.jp/entry/tnl:113494316) [K01810](https://www.genome.jp/entry/K01810) glucose-6-phosphate isomerase

[tnl:113495499](https://www.genome.jp/entry/tnl:113495499) [K01836](https://www.genome.jp/entry/K01836) phosphoacetylglucosamine mutase

[tnl:113496554](https://www.genome.jp/entry/tnl:113496554) [K02377](https://www.genome.jp/entry/K02377) GDP-L-fucose synthase isoform X1

[tnl:113497947](https://www.genome.jp/entry/tnl:113497947) [K00844](https://www.genome.jp/entry/K00844) hexokinase type 2 isoform X1

[tnl:113501660](https://www.genome.jp/entry/tnl:113501660) [K00963](https://www.genome.jp/entry/K00963) UTP--glucose-1-phosphate uridylyltransferase isoform X1

[tnl:113503659](https://www.genome.jp/entry/tnl:113503659) [K01835](https://www.genome.jp/entry/K01835) phosphoglucomutase

[tnl:113503711](https://www.genome.jp/entry/tnl:113503711) [K01784](https://www.genome.jp/entry/K01784) UDP-glucose 4-epimerase-like

[tnl:113503730](https://www.genome.jp/entry/tnl:113503730) [K01711](https://www.genome.jp/entry/K01711) GDP-mannose 4,6 dehydratase

[tnl:113505855](https://www.genome.jp/entry/tnl:113505855) [K01809](https://www.genome.jp/entry/K01809) mannose-6-phosphate isomerase

[tnl:113507917](https://www.genome.jp/entry/tnl:113507917) [K00012](https://www.genome.jp/entry/K00012) UDP-glucose 6-dehydrogenase

[tnl:113508098](https://www.genome.jp/entry/tnl:113508098) [K17497](https://www.genome.jp/entry/K17497) phosphomannomutase 2

 [**tnl04137**](https://www.genome.jp/kegg-bin/show_pathway?166859291342340/tnl04137.args) **Mitophagy - animal - Trichoplusia ni (cabbage looper) (**[**12**](javascript:display('tnl04137'))**)**

[tnl:113492520](https://www.genome.jp/entry/tnl:113492520) [K08339](https://www.genome.jp/entry/K08339) autophagy protein 5

[tnl:113492607](https://www.genome.jp/entry/tnl:113492607) [K07897](https://www.genome.jp/entry/K07897) ras-related protein Rab-7a

[tnl:113493805](https://www.genome.jp/entry/tnl:113493805) [K07827](https://www.genome.jp/entry/K07827) GTPase HRas

[tnl:113495885](https://www.genome.jp/entry/tnl:113495885) [K08341](https://www.genome.jp/entry/K08341) gamma-aminobutyric acid receptor-associated protein

[tnl:113495905](https://www.genome.jp/entry/tnl:113495905) [K17907](https://www.genome.jp/entry/K17907) autophagy-related protein 9A

[tnl:113496312](https://www.genome.jp/entry/tnl:113496312) [K03097](https://www.genome.jp/entry/K03097) casein kinase II subunit alpha isoform X1

[tnl:113496361](https://www.genome.jp/entry/tnl:113496361) [K14381](https://www.genome.jp/entry/K14381) sequestosome-1-like isoform X1

[tnl:113497265](https://www.genome.jp/entry/tnl:113497265) [K05704](https://www.genome.jp/entry/K05704) tyrosine-protein kinase Src64B isoform X1

[tnl:113502222](https://www.genome.jp/entry/tnl:113502222) [K07830](https://www.genome.jp/entry/K07830) ras-like protein 2

[tnl:113503581](https://www.genome.jp/entry/tnl:113503581) [K08770](https://www.genome.jp/entry/K08770) polyubiquitin-A

[tnl:113503670](https://www.genome.jp/entry/tnl:113503670) [K04440](https://www.genome.jp/entry/K04440) stress-activated protein kinase JNK isoform X1

[tnl:113506374](https://www.genome.jp/entry/tnl:113506374) [K02977](https://www.genome.jp/entry/K02977) ubiquitin-40S ribosomal protein S27a

**** [**tnl04512**](https://www.genome.jp/kegg-bin/show_pathway?166859291342340/tnl04512.args) **ECM-receptor interaction - Trichoplusia ni (cabbage looper) (**[**12**](javascript:display('tnl04512'))**)**

[tnl:113497504](https://www.genome.jp/entry/tnl:113497504) [K06483](https://www.genome.jp/entry/K06483) integrin alpha-PS4-like isoform X1

[tnl:113499965](https://www.genome.jp/entry/tnl:113499965) [K05635](https://www.genome.jp/entry/K05635) laminin subunit gamma-1

[tnl:113499980](https://www.genome.jp/entry/tnl:113499980) [K06240](https://www.genome.jp/entry/K06240) LOW QUALITY PROTEIN: laminin subunit alpha

[tnl:113501210](https://www.genome.jp/entry/tnl:113501210) [K06237](https://www.genome.jp/entry/K06237) collagen alpha-1(IV) chain

[tnl:113501211](https://www.genome.jp/entry/tnl:113501211) [K06237](https://www.genome.jp/entry/K06237) collagen alpha-1(IV) chain

[tnl:113501373](https://www.genome.jp/entry/tnl:113501373) [K05637](https://www.genome.jp/entry/K05637) laminin subunit alpha-1-like

[tnl:113501391](https://www.genome.jp/entry/tnl:113501391) [K06265](https://www.genome.jp/entry/K06265) LOW QUALITY PROTEIN: dystroglycan-like

[tnl:113504222](https://www.genome.jp/entry/tnl:113504222) [K06265](https://www.genome.jp/entry/K06265) LOW QUALITY PROTEIN: dystroglycan-like

[tnl:113504314](https://www.genome.jp/entry/tnl:113504314) [K06258](https://www.genome.jp/entry/K06258) uncharacterized protein LOC113504314

[tnl:113504609](https://www.genome.jp/entry/tnl:113504609) [K05719](https://www.genome.jp/entry/K05719) integrin beta-PS

[tnl:113508286](https://www.genome.jp/entry/tnl:113508286) [K05636](https://www.genome.jp/entry/K05636) laminin subunit beta-1

[tnl:113508828](https://www.genome.jp/entry/tnl:113508828) [K06584](https://www.genome.jp/entry/K06584) integrin alpha-PS2 isoform X1

 [**tnl03018**](https://www.genome.jp/kegg-bin/show_pathway?166859291342340/tnl03018.args) **RNA degradation - Trichoplusia ni (cabbage looper) (**[**12**](javascript:display('tnl03018'))**)**

[tnl:113496310](https://www.genome.jp/entry/tnl:113496310) [K00850](https://www.genome.jp/entry/K00850) ATP-dependent 6-phosphofructokinase isoform X1

[tnl:113496349](https://www.genome.jp/entry/tnl:113496349) [K13126](https://www.genome.jp/entry/K13126) polyadenylate-binding protein 1-A

[tnl:113496362](https://www.genome.jp/entry/tnl:113496362) [K12589](https://www.genome.jp/entry/K12589) exosome complex component RRP42

[tnl:113497171](https://www.genome.jp/entry/tnl:113497171) [K12614](https://www.genome.jp/entry/K12614) putative ATP-dependent RNA helicase me31b

[tnl:113499004](https://www.genome.jp/entry/tnl:113499004) [K01689](https://www.genome.jp/entry/K01689) enolase

[tnl:113499084](https://www.genome.jp/entry/tnl:113499084) [K12611](https://www.genome.jp/entry/K12611) mRNA-decapping enzyme 1A

[tnl:113499093](https://www.genome.jp/entry/tnl:113499093) [K12599](https://www.genome.jp/entry/K12599) helicase SKI2W

[tnl:113501751](https://www.genome.jp/entry/tnl:113501751) [K03681](https://www.genome.jp/entry/K03681) exosome complex component RRP40

[tnl:113503680](https://www.genome.jp/entry/tnl:113503680) [K04077](https://www.genome.jp/entry/K04077) heat shock protein 60A-like

[tnl:113505692](https://www.genome.jp/entry/tnl:113505692) [K04043](https://www.genome.jp/entry/K04043) heat shock 70 kDa protein cognate 5

[tnl:113506519](https://www.genome.jp/entry/tnl:113506519) [K12625](https://www.genome.jp/entry/K12625) U6 snRNA-associated Sm-like protein LSm6

[tnl:113507019](https://www.genome.jp/entry/tnl:113507019) [K12600](https://www.genome.jp/entry/K12600) tetratricopeptide repeat protein 37

**** [**tnl00980**](https://www.genome.jp/kegg-bin/show_pathway?166859291342340/tnl00980.args) **Metabolism of xenobiotics by cytochrome P450 - Trichoplusia ni (cabbage looper) (**[**12**](javascript:display('tnl00980'))**)**

[tnl:113493465](https://www.genome.jp/entry/tnl:113493465) [K00079](https://www.genome.jp/entry/K00079) carbonyl reductase [NADPH] 1-like

[tnl:113494468](https://www.genome.jp/entry/tnl:113494468) [K00079](https://www.genome.jp/entry/K00079) carbonyl reductase [NADPH] 3-like

[tnl:113494907](https://www.genome.jp/entry/tnl:113494907) [K00799](https://www.genome.jp/entry/K00799) glutathione S-transferase 1-like

[tnl:113496731](https://www.genome.jp/entry/tnl:113496731) [K00799](https://www.genome.jp/entry/K00799) uncharacterized protein LOC113496731

[tnl:113497008](https://www.genome.jp/entry/tnl:113497008) [K00799](https://www.genome.jp/entry/K00799) glutathione S-transferase 1-1-like

[tnl:113497009](https://www.genome.jp/entry/tnl:113497009) [K00799](https://www.genome.jp/entry/K00799) glutathione S-transferase 1-1-like isoform X1

[tnl:113497263](https://www.genome.jp/entry/tnl:113497263) [K04097](https://www.genome.jp/entry/K04097) glutathione S-transferase 2-like

[tnl:113497863](https://www.genome.jp/entry/tnl:113497863) [K00121](https://www.genome.jp/entry/K00121) alcohol dehydrogenase class-3

[tnl:113497936](https://www.genome.jp/entry/tnl:113497936) [K00079](https://www.genome.jp/entry/K00079) carbonyl reductase [NADPH] 1-like

[tnl:113499552](https://www.genome.jp/entry/tnl:113499552) [K00799](https://www.genome.jp/entry/K00799) uncharacterized protein LOC113499552

[tnl:113507103](https://www.genome.jp/entry/tnl:113507103) [K00799](https://www.genome.jp/entry/K00799) glutathione S-transferase 1-1-like

[tnl:113508050](https://www.genome.jp/entry/tnl:113508050) [K00799](https://www.genome.jp/entry/K00799) glutathione S-transferase 1-1-like

 [**tnl00250**](https://www.genome.jp/kegg-bin/show_pathway?166859291342340/tnl00250.args) **Alanine, aspartate and glutamate metabolism - Trichoplusia ni (cabbage looper) (**[**11**](javascript:display('tnl00250'))**)**

[tnl:113492937](https://www.genome.jp/entry/tnl:113492937) [K14454](https://www.genome.jp/entry/K14454) aspartate aminotransferase, cytoplasmic

[tnl:113493809](https://www.genome.jp/entry/tnl:113493809) [K13566](https://www.genome.jp/entry/K13566) omega-amidase NIT2-like isoform X1

[tnl:113494579](https://www.genome.jp/entry/tnl:113494579) [K00764](https://www.genome.jp/entry/K00764) amidophosphoribosyltransferase-like isoform X1

[tnl:113494797](https://www.genome.jp/entry/tnl:113494797) [K01756](https://www.genome.jp/entry/K01756) adenylosuccinate lyase-like

[tnl:113495260](https://www.genome.jp/entry/tnl:113495260) [K01953](https://www.genome.jp/entry/K01953) asparagine synthetase [glutamine-hydrolyzing]

[tnl:113496121](https://www.genome.jp/entry/tnl:113496121) [K01915](https://www.genome.jp/entry/K01915) glutamine synthetase 2 cytoplasmic-like isoform X1

[tnl:113496832](https://www.genome.jp/entry/tnl:113496832) [K00261](https://www.genome.jp/entry/K00261) glutamate dehydrogenase, mitochondrial

[tnl:113498369](https://www.genome.jp/entry/tnl:113498369) [K00814](https://www.genome.jp/entry/K00814) alanine aminotransferase 1-like isoform X1

[tnl:113504558](https://www.genome.jp/entry/tnl:113504558) [K11540](https://www.genome.jp/entry/K11540) CAD protein isoform X1

[tnl:113504856](https://www.genome.jp/entry/tnl:113504856) [K01939](https://www.genome.jp/entry/K01939) adenylosuccinate synthetase-like

[tnl:113507969](https://www.genome.jp/entry/tnl:113507969) [K01939](https://www.genome.jp/entry/K01939) LOW QUALITY PROTEIN: adenylosuccinate synthetase-like

**** [**tnl00500**](https://www.genome.jp/kegg-bin/show_pathway?166859291342340/tnl00500.args) **Starch and sucrose metabolism - Trichoplusia ni (cabbage looper) (**[**11**](javascript:display('tnl00500'))**)**

[tnl:113494316](https://www.genome.jp/entry/tnl:113494316) [K01810](https://www.genome.jp/entry/K01810) glucose-6-phosphate isomerase

[tnl:113495376](https://www.genome.jp/entry/tnl:113495376) [K01196](https://www.genome.jp/entry/K01196) glycogen debranching enzyme

[tnl:113496976](https://www.genome.jp/entry/tnl:113496976) [K00750](https://www.genome.jp/entry/K00750) uncharacterized protein LOC113496976 isoform X1

[tnl:113497652](https://www.genome.jp/entry/tnl:113497652) [K00688](https://www.genome.jp/entry/K00688) glycogen phosphorylase

[tnl:113497839](https://www.genome.jp/entry/tnl:113497839) [K12316](https://www.genome.jp/entry/K12316) lysosomal alpha-glucosidase-like

[tnl:113497947](https://www.genome.jp/entry/tnl:113497947) [K00844](https://www.genome.jp/entry/K00844) hexokinase type 2 isoform X1

[tnl:113498597](https://www.genome.jp/entry/tnl:113498597) [K00693](https://www.genome.jp/entry/K00693) glycogen [starch] synthase

[tnl:113501660](https://www.genome.jp/entry/tnl:113501660) [K00963](https://www.genome.jp/entry/K00963) UTP--glucose-1-phosphate uridylyltransferase isoform X1

[tnl:113503659](https://www.genome.jp/entry/tnl:113503659) [K01835](https://www.genome.jp/entry/K01835) phosphoglucomutase

[tnl:113508734](https://www.genome.jp/entry/tnl:113508734) [K01194](https://www.genome.jp/entry/K01194) uncharacterized protein LOC113508734

[tnl:113508887](https://www.genome.jp/entry/tnl:113508887) [K00700](https://www.genome.jp/entry/K00700) 1,4-alpha-glucan-branching enzyme

 [**tnl00630**](https://www.genome.jp/kegg-bin/show_pathway?166859291342340/tnl00630.args) **Glyoxylate and dicarboxylate metabolism - Trichoplusia ni (cabbage looper) (**[**10**](javascript:display('tnl00630'))**)**

[tnl:113491873](https://www.genome.jp/entry/tnl:113491873) [K00600](https://www.genome.jp/entry/K00600) serine hydroxymethyltransferase, cytosolic isoform X1

[tnl:113492551](https://www.genome.jp/entry/tnl:113492551) [K01432](https://www.genome.jp/entry/K01432) kynurenine formamidase isoform X1

[tnl:113495760](https://www.genome.jp/entry/tnl:113495760) [K03781](https://www.genome.jp/entry/K03781) catalase

[tnl:113496121](https://www.genome.jp/entry/tnl:113496121) [K01915](https://www.genome.jp/entry/K01915) glutamine synthetase 2 cytoplasmic-like isoform X1

[tnl:113497192](https://www.genome.jp/entry/tnl:113497192) [K00049](https://www.genome.jp/entry/K00049) glyoxylate reductase/hydroxypyruvate reductase-like

[tnl:113497832](https://www.genome.jp/entry/tnl:113497832) [K00025](https://www.genome.jp/entry/K00025) malate dehydrogenase, cytoplasmic

[tnl:113498020](https://www.genome.jp/entry/tnl:113498020) [K00626](https://www.genome.jp/entry/K00626) acetyl-CoA acetyltransferase, mitochondrial

[tnl:113499205](https://www.genome.jp/entry/tnl:113499205) [K01816](https://www.genome.jp/entry/K01816) putative hydroxypyruvate isomerase

[tnl:113501275](https://www.genome.jp/entry/tnl:113501275) [K19269](https://www.genome.jp/entry/K19269) glycerol-3-phosphate phosphatase-like

[tnl:113507140](https://www.genome.jp/entry/tnl:113507140) [K00049](https://www.genome.jp/entry/K00049) LOW QUALITY PROTEIN: glyoxylate reductase/hydroxypyruvate reductase-like

 [**tnl00330**](https://www.genome.jp/kegg-bin/show_pathway?166859291342340/tnl00330.args) **Arginine and proline metabolism - Trichoplusia ni (cabbage looper) (**[**10**](javascript:display('tnl00330'))**)**

[tnl:113492664](https://www.genome.jp/entry/tnl:113492664) [K08660](https://www.genome.jp/entry/K08660) cytosolic non-specific dipeptidase

[tnl:113492937](https://www.genome.jp/entry/tnl:113492937) [K14454](https://www.genome.jp/entry/K14454) aspartate aminotransferase, cytoplasmic

[tnl:113493022](https://www.genome.jp/entry/tnl:113493022) [K00657](https://www.genome.jp/entry/K00657) diamine acetyltransferase 2-like

[tnl:113496805](https://www.genome.jp/entry/tnl:113496805) [K00286](https://www.genome.jp/entry/K00286) pyrroline-5-carboxylate reductase-like

[tnl:113497383](https://www.genome.jp/entry/tnl:113497383) [K00472](https://www.genome.jp/entry/K00472) prolyl 4-hydroxylase subunit alpha-1-like

[tnl:113497424](https://www.genome.jp/entry/tnl:113497424) [K00797](https://www.genome.jp/entry/K00797) spermidine synthase

[tnl:113500921](https://www.genome.jp/entry/tnl:113500921) [K00128](https://www.genome.jp/entry/K00128) retinal dehydrogenase 1-like

[tnl:113501645](https://www.genome.jp/entry/tnl:113501645) [K00934](https://www.genome.jp/entry/K00934) arginine kinase isoform X1

[tnl:113502268](https://www.genome.jp/entry/tnl:113502268) [K00802](https://www.genome.jp/entry/K00802) spermine synthase

[tnl:113503573](https://www.genome.jp/entry/tnl:113503573) [K00128](https://www.genome.jp/entry/K00128) aldehyde dehydrogenase, mitochondrial

 [**tnl00030**](https://www.genome.jp/kegg-bin/show_pathway?166859291342340/tnl00030.args) **Pentose phosphate pathway - Trichoplusia ni (cabbage looper) (**[**10**](javascript:display('tnl00030'))**)**

[tnl:113493828](https://www.genome.jp/entry/tnl:113493828) [K00948](https://www.genome.jp/entry/K00948) ribose-phosphate pyrophosphokinase 2

[tnl:113494316](https://www.genome.jp/entry/tnl:113494316) [K01810](https://www.genome.jp/entry/K01810) glucose-6-phosphate isomerase

[tnl:113496310](https://www.genome.jp/entry/tnl:113496310) [K00850](https://www.genome.jp/entry/K00850) ATP-dependent 6-phosphofructokinase isoform X1

[tnl:113499721](https://www.genome.jp/entry/tnl:113499721) [K00615](https://www.genome.jp/entry/K00615) transketolase-like protein 2

[tnl:113500261](https://www.genome.jp/entry/tnl:113500261) [K01053](https://www.genome.jp/entry/K01053) regucalcin-like isoform X1

[tnl:113501060](https://www.genome.jp/entry/tnl:113501060) [K00036](https://www.genome.jp/entry/K00036) glucose-6-phosphate 1-dehydrogenase

[tnl:113503107](https://www.genome.jp/entry/tnl:113503107) [K01057](https://www.genome.jp/entry/K01057) 6-phosphogluconolactonase

[tnl:113503659](https://www.genome.jp/entry/tnl:113503659) [K01835](https://www.genome.jp/entry/K01835) phosphoglucomutase

[tnl:113508324](https://www.genome.jp/entry/tnl:113508324) [K00616](https://www.genome.jp/entry/K00616) probable transaldolase

[tnl:113508342](https://www.genome.jp/entry/tnl:113508342) [K01807](https://www.genome.jp/entry/K01807) ribose-5-phosphate isomerase

 [**tnl01212**](https://www.genome.jp/kegg-bin/show_pathway?166859291342340/tnl01212.args) **Fatty acid metabolism - Trichoplusia ni (cabbage looper) (**[**10**](javascript:display('tnl01212'))**)**

[tnl:113493061](https://www.genome.jp/entry/tnl:113493061) [K01897](https://www.genome.jp/entry/K01897) long-chain-fatty-acid--CoA ligase 4 isoform X1

[tnl:113493488](https://www.genome.jp/entry/tnl:113493488) [K07515](https://www.genome.jp/entry/K07515) trifunctional enzyme subunit alpha, mitochondrial

[tnl:113494349](https://www.genome.jp/entry/tnl:113494349) [K07509](https://www.genome.jp/entry/K07509) LOW QUALITY PROTEIN: trifunctional enzyme subunit beta, mitochondrial-like

[tnl:113497645](https://www.genome.jp/entry/tnl:113497645) [K00507](https://www.genome.jp/entry/K00507) acyl-CoA Delta(11) desaturase-like

[tnl:113498020](https://www.genome.jp/entry/tnl:113498020) [K00626](https://www.genome.jp/entry/K00626) acetyl-CoA acetyltransferase, mitochondrial

[tnl:113498344](https://www.genome.jp/entry/tnl:113498344) [K00665](https://www.genome.jp/entry/K00665) fatty acid synthase isoform X1

[tnl:113498903](https://www.genome.jp/entry/tnl:113498903) [K11262](https://www.genome.jp/entry/K11262) acetyl-CoA carboxylase isoform X1

[tnl:113498996](https://www.genome.jp/entry/tnl:113498996) [K08765](https://www.genome.jp/entry/K08765) carnitine O-palmitoyltransferase 1, liver isoform

[tnl:113506203](https://www.genome.jp/entry/tnl:113506203) [K07509](https://www.genome.jp/entry/K07509) trifunctional enzyme subunit beta, mitochondrial-like

[tnl:113507884](https://www.genome.jp/entry/tnl:113507884) [K00249](https://www.genome.jp/entry/K00249) probable medium-chain specific acyl-CoA dehydrogenase, mitochondrial isoform X1

**** [**tnl04330**](https://www.genome.jp/kegg-bin/show_pathway?166859291342340/tnl04330.args) **Notch signaling pathway - Trichoplusia ni (cabbage looper) (**[**10**](javascript:display('tnl04330'))**)**

[tnl:113492661](https://www.genome.jp/entry/tnl:113492661) [K06171](https://www.genome.jp/entry/K06171) nicastrin

[tnl:113494028](https://www.genome.jp/entry/tnl:113494028) [K02599](https://www.genome.jp/entry/K02599) neurogenic locus Notch protein

[tnl:113496483](https://www.genome.jp/entry/tnl:113496483) [K06067](https://www.genome.jp/entry/K06067) LOW QUALITY PROTEIN: histone deacetylase Rpd3-like

[tnl:113496752](https://www.genome.jp/entry/tnl:113496752) [K06067](https://www.genome.jp/entry/K06067) histone deacetylase Rpd3-like

[tnl:113497334](https://www.genome.jp/entry/tnl:113497334) [K06057](https://www.genome.jp/entry/K06057) protein numb isoform X1

[tnl:113497524](https://www.genome.jp/entry/tnl:113497524) [K06172](https://www.genome.jp/entry/K06172) gamma-secretase subunit Aph-1

[tnl:113502063](https://www.genome.jp/entry/tnl:113502063) [K06058](https://www.genome.jp/entry/K06058) protein deltex

[tnl:113506733](https://www.genome.jp/entry/tnl:113506733) [K04505](https://www.genome.jp/entry/K04505) presenilin homolog isoform X1

[tnl:113507914](https://www.genome.jp/entry/tnl:113507914) [K02353](https://www.genome.jp/entry/K02353) LOW QUALITY PROTEIN: segment polarity protein dishevelled homolog DVL-3-like

[tnl:113507920](https://www.genome.jp/entry/tnl:113507920) [K02353](https://www.genome.jp/entry/K02353) LOW QUALITY PROTEIN: segment polarity protein dishevelled homolog DVL-3-like

**** [**tnl03250**](https://www.genome.jp/kegg-bin/show_pathway?166859291342340/tnl03250.args) **Viral life cycle - HIV-1 - Trichoplusia ni (cabbage looper) (**[**10**](javascript:display('tnl03250'))**)**

[tnl:113491707](https://www.genome.jp/entry/tnl:113491707) [K07936](https://www.genome.jp/entry/K07936) GTP-binding nuclear protein Ran

[tnl:113491861](https://www.genome.jp/entry/tnl:113491861) [K12195](https://www.genome.jp/entry/K12195) charged multivesicular body protein 6-A

[tnl:113491902](https://www.genome.jp/entry/tnl:113491902) [K12182](https://www.genome.jp/entry/K12182) hepatocyte growth factor-regulated tyrosine kinase substrate

[tnl:113493341](https://www.genome.jp/entry/tnl:113493341) [K12200](https://www.genome.jp/entry/K12200) programmed cell death 6-interacting protein

[tnl:113496575](https://www.genome.jp/entry/tnl:113496575) [K12196](https://www.genome.jp/entry/K12196) vacuolar protein sorting-associated protein 4A-like

[tnl:113497709](https://www.genome.jp/entry/tnl:113497709) [K15436](https://www.genome.jp/entry/K15436) transportin-3

[tnl:113497767](https://www.genome.jp/entry/tnl:113497767) [K14290](https://www.genome.jp/entry/K14290) exportin-1

[tnl:113499278](https://www.genome.jp/entry/tnl:113499278) [K12194](https://www.genome.jp/entry/K12194) charged multivesicular body protein 4b

[tnl:113500207](https://www.genome.jp/entry/tnl:113500207) [K14398](https://www.genome.jp/entry/K14398) cleavage and polyadenylation specificity factor subunit CG7185

[tnl:113505472](https://www.genome.jp/entry/tnl:113505472) [K12183](https://www.genome.jp/entry/K12183) tumor susceptibility gene 101 protein

**** [**tnl00071**](https://www.genome.jp/kegg-bin/show_pathway?166859291342340/tnl00071.args) **Fatty acid degradation - Trichoplusia ni (cabbage looper) (**[**10**](javascript:display('tnl00071'))**)**

[tnl:113493061](https://www.genome.jp/entry/tnl:113493061) [K01897](https://www.genome.jp/entry/K01897) long-chain-fatty-acid--CoA ligase 4 isoform X1

[tnl:113493488](https://www.genome.jp/entry/tnl:113493488) [K07515](https://www.genome.jp/entry/K07515) trifunctional enzyme subunit alpha, mitochondrial

[tnl:113494349](https://www.genome.jp/entry/tnl:113494349) [K07509](https://www.genome.jp/entry/K07509) LOW QUALITY PROTEIN: trifunctional enzyme subunit beta, mitochondrial-like

[tnl:113497863](https://www.genome.jp/entry/tnl:113497863) [K00121](https://www.genome.jp/entry/K00121) alcohol dehydrogenase class-3

[tnl:113498020](https://www.genome.jp/entry/tnl:113498020) [K00626](https://www.genome.jp/entry/K00626) acetyl-CoA acetyltransferase, mitochondrial

[tnl:113498996](https://www.genome.jp/entry/tnl:113498996) [K08765](https://www.genome.jp/entry/K08765) carnitine O-palmitoyltransferase 1, liver isoform

[tnl:113500921](https://www.genome.jp/entry/tnl:113500921) [K00128](https://www.genome.jp/entry/K00128) retinal dehydrogenase 1-like

[tnl:113503573](https://www.genome.jp/entry/tnl:113503573) [K00128](https://www.genome.jp/entry/K00128) aldehyde dehydrogenase, mitochondrial

[tnl:113506203](https://www.genome.jp/entry/tnl:113506203) [K07509](https://www.genome.jp/entry/K07509) trifunctional enzyme subunit beta, mitochondrial-like

[tnl:113507884](https://www.genome.jp/entry/tnl:113507884) [K00249](https://www.genome.jp/entry/K00249) probable medium-chain specific acyl-CoA dehydrogenase, mitochondrial isoform X1

 [**tnl04146**](https://www.genome.jp/kegg-bin/show_pathway?166859291342340/tnl04146.args) **Peroxisome - Trichoplusia ni (cabbage looper) (**[**10**](javascript:display('tnl04146'))**)**

[tnl:113492710](https://www.genome.jp/entry/tnl:113492710) [K00031](https://www.genome.jp/entry/K00031) isocitrate dehydrogenase [NADP] cytoplasmic-like

[tnl:113493007](https://www.genome.jp/entry/tnl:113493007) [K13279](https://www.genome.jp/entry/K13279) peroxiredoxin 1 isoform X1

[tnl:113493061](https://www.genome.jp/entry/tnl:113493061) [K01897](https://www.genome.jp/entry/K01897) long-chain-fatty-acid--CoA ligase 4 isoform X1

[tnl:113495760](https://www.genome.jp/entry/tnl:113495760) [K03781](https://www.genome.jp/entry/K03781) catalase

[tnl:113497711](https://www.genome.jp/entry/tnl:113497711) [K11187](https://www.genome.jp/entry/K11187) peroxiredoxin-5, mitochondrial

[tnl:113497732](https://www.genome.jp/entry/tnl:113497732) [K00106](https://www.genome.jp/entry/K00106) xanthine dehydrogenase

[tnl:113502380](https://www.genome.jp/entry/tnl:113502380) [K13337](https://www.genome.jp/entry/K13337) peroxisomal biogenesis factor 19

[tnl:113504438](https://www.genome.jp/entry/tnl:113504438) [K00031](https://www.genome.jp/entry/K00031) isocitrate dehydrogenase [NADP] cytoplasmic isoform X1

[tnl:113504501](https://www.genome.jp/entry/tnl:113504501) [K04565](https://www.genome.jp/entry/K04565) superoxide dismutase [Cu-Zn] 2-like

[tnl:113505811](https://www.genome.jp/entry/tnl:113505811) [K04565](https://www.genome.jp/entry/K04565) superoxide dismutase [Cu-Zn]

**** [**tnl04341**](https://www.genome.jp/kegg-bin/show_pathway?166859291342340/tnl04341.args) **Hedgehog signaling pathway - fly - Trichoplusia ni (cabbage looper) (**[**9**](javascript:display('tnl04341'))**)**

[tnl:113491887](https://www.genome.jp/entry/tnl:113491887) [K04678](https://www.genome.jp/entry/K04678) E3 ubiquitin-protein ligase SMURF2

[tnl:113495152](https://www.genome.jp/entry/tnl:113495152) [K08291](https://www.genome.jp/entry/K08291) G protein-coupled receptor kinase 2 isoform X1

[tnl:113495537](https://www.genome.jp/entry/tnl:113495537) [K20245](https://www.genome.jp/entry/K20245) interference hedgehog-like

[tnl:113501076](https://www.genome.jp/entry/tnl:113501076) [K03869](https://www.genome.jp/entry/K03869) cullin-3 isoform X1

[tnl:113501710](https://www.genome.jp/entry/tnl:113501710) [K06225](https://www.genome.jp/entry/K06225) protein patched

[tnl:113503417](https://www.genome.jp/entry/tnl:113503417) [K08958](https://www.genome.jp/entry/K08958) casein kinase I isoform X1

[tnl:113506804](https://www.genome.jp/entry/tnl:113506804) [K04345](https://www.genome.jp/entry/K04345) cAMP-dependent protein kinase catalytic subunit

[tnl:113507076](https://www.genome.jp/entry/tnl:113507076) [K06229](https://www.genome.jp/entry/K06229) suppressor of fused homolog

[tnl:113508299](https://www.genome.jp/entry/tnl:113508299) [K03094](https://www.genome.jp/entry/K03094) S-phase kinase-associated protein 1

**** [**tnl00790**](https://www.genome.jp/kegg-bin/show_pathway?166859291342340/tnl00790.args) **Folate biosynthesis - Trichoplusia ni (cabbage looper) (**[**9**](javascript:display('tnl00790'))**)**

[tnl:113493465](https://www.genome.jp/entry/tnl:113493465) [K00079](https://www.genome.jp/entry/K00079) carbonyl reductase [NADPH] 1-like

[tnl:113494468](https://www.genome.jp/entry/tnl:113494468) [K00079](https://www.genome.jp/entry/K00079) carbonyl reductase [NADPH] 3-like

[tnl:113497302](https://www.genome.jp/entry/tnl:113497302) [K00072](https://www.genome.jp/entry/K00072) sepiapterin reductase

[tnl:113497936](https://www.genome.jp/entry/tnl:113497936) [K00079](https://www.genome.jp/entry/K00079) carbonyl reductase [NADPH] 1-like

[tnl:113501099](https://www.genome.jp/entry/tnl:113501099) [K00310](https://www.genome.jp/entry/K00310) pyrimidodiazepine synthase-like

[tnl:113502955](https://www.genome.jp/entry/tnl:113502955) [K15376](https://www.genome.jp/entry/K15376) gephyrin

[tnl:113503650](https://www.genome.jp/entry/tnl:113503650) [K01077](https://www.genome.jp/entry/K01077) alkaline phosphatase-like

[tnl:113504172](https://www.genome.jp/entry/tnl:113504172) [K00287](https://www.genome.jp/entry/K00287) dihydrofolate reductase isoform X1

[tnl:113505357](https://www.genome.jp/entry/tnl:113505357) [K00357](https://www.genome.jp/entry/K00357) dihydropteridine reductase

**** [**tnl00982**](https://www.genome.jp/kegg-bin/show_pathway?166859291342340/tnl00982.args) **Drug metabolism - cytochrome P450 - Trichoplusia ni (cabbage looper) (**[**9**](javascript:display('tnl00982'))**)**

[tnl:113494907](https://www.genome.jp/entry/tnl:113494907) [K00799](https://www.genome.jp/entry/K00799) glutathione S-transferase 1-like

[tnl:113496731](https://www.genome.jp/entry/tnl:113496731) [K00799](https://www.genome.jp/entry/K00799) uncharacterized protein LOC113496731

[tnl:113497008](https://www.genome.jp/entry/tnl:113497008) [K00799](https://www.genome.jp/entry/K00799) glutathione S-transferase 1-1-like

[tnl:113497009](https://www.genome.jp/entry/tnl:113497009) [K00799](https://www.genome.jp/entry/K00799) glutathione S-transferase 1-1-like isoform X1

[tnl:113497263](https://www.genome.jp/entry/tnl:113497263) [K04097](https://www.genome.jp/entry/K04097) glutathione S-transferase 2-like

[tnl:113497863](https://www.genome.jp/entry/tnl:113497863) [K00121](https://www.genome.jp/entry/K00121) alcohol dehydrogenase class-3

[tnl:113499552](https://www.genome.jp/entry/tnl:113499552) [K00799](https://www.genome.jp/entry/K00799) uncharacterized protein LOC113499552

[tnl:113507103](https://www.genome.jp/entry/tnl:113507103) [K00799](https://www.genome.jp/entry/K00799) glutathione S-transferase 1-1-like

[tnl:113508050](https://www.genome.jp/entry/tnl:113508050) [K00799](https://www.genome.jp/entry/K00799) glutathione S-transferase 1-1-like

**** [**tnl04320**](https://www.genome.jp/kegg-bin/show_pathway?166859291342340/tnl04320.args) **Dorso-ventral axis formation - Trichoplusia ni (cabbage looper) (**[**9**](javascript:display('tnl04320'))**)**

[tnl:113491757](https://www.genome.jp/entry/tnl:113491757) [K04371](https://www.genome.jp/entry/K04371) mitogen-activated protein kinase ERK-A

[tnl:113492015](https://www.genome.jp/entry/tnl:113492015) [K04364](https://www.genome.jp/entry/K04364) protein enhancer of sevenless 2B

[tnl:113493348](https://www.genome.jp/entry/tnl:113493348) [K03102](https://www.genome.jp/entry/K03102) RNA-binding protein squid isoform X1

[tnl:113493668](https://www.genome.jp/entry/tnl:113493668) [K04365](https://www.genome.jp/entry/K04365) raf homolog serine/threonine-protein kinase Raf isoform X1

[tnl:113493805](https://www.genome.jp/entry/tnl:113493805) [K07827](https://www.genome.jp/entry/K07827) GTPase HRas

[tnl:113494028](https://www.genome.jp/entry/tnl:113494028) [K02599](https://www.genome.jp/entry/K02599) neurogenic locus Notch protein

[tnl:113497222](https://www.genome.jp/entry/tnl:113497222) [K02156](https://www.genome.jp/entry/K02156) piwi-like protein Siwi

[tnl:113503523](https://www.genome.jp/entry/tnl:113503523) [K04368](https://www.genome.jp/entry/K04368) dual specificity mitogen-activated protein kinase kinase dSOR1

[tnl:113503612](https://www.genome.jp/entry/tnl:113503612) [K02104](https://www.genome.jp/entry/K02104) protein giant-lens

**** [**tnl00260**](https://www.genome.jp/kegg-bin/show_pathway?166859291342340/tnl00260.args) **Glycine, serine and threonine metabolism - Trichoplusia ni (cabbage looper) (**[**8**](javascript:display('tnl00260'))**)**

[tnl:113491873](https://www.genome.jp/entry/tnl:113491873) [K00600](https://www.genome.jp/entry/K00600) serine hydroxymethyltransferase, cytosolic isoform X1

[tnl:113496177](https://www.genome.jp/entry/tnl:113496177) [K00058](https://www.genome.jp/entry/K00058) D-3-phosphoglycerate dehydrogenase

[tnl:113497192](https://www.genome.jp/entry/tnl:113497192) [K00049](https://www.genome.jp/entry/K00049) glyoxylate reductase/hydroxypyruvate reductase-like

[tnl:113500574](https://www.genome.jp/entry/tnl:113500574) [K00831](https://www.genome.jp/entry/K00831) probable phosphoserine aminotransferase

[tnl:113502164](https://www.genome.jp/entry/tnl:113502164) [K01758](https://www.genome.jp/entry/K01758) cystathionine gamma-lyase-like

[tnl:113504890](https://www.genome.jp/entry/tnl:113504890) [K01697](https://www.genome.jp/entry/K01697) cystathionine beta-synthase-like

[tnl:113506529](https://www.genome.jp/entry/tnl:113506529) [K01834](https://www.genome.jp/entry/K01834) phosphoglycerate mutase 2-like

[tnl:113507140](https://www.genome.jp/entry/tnl:113507140) [K00049](https://www.genome.jp/entry/K00049) LOW QUALITY PROTEIN: glyoxylate reductase/hydroxypyruvate reductase-like

**** [**tnl00380**](https://www.genome.jp/kegg-bin/show_pathway?166859291342340/tnl00380.args) **Tryptophan metabolism - Trichoplusia ni (cabbage looper) (**[**8**](javascript:display('tnl00380'))**)**

[tnl:113492358](https://www.genome.jp/entry/tnl:113492358) [K00658](https://www.genome.jp/entry/K00658) dihydrolipoyllysine-residue succinyltransferase component of 2-oxoglutarate dehydrogenase complex, mitochondrial-like

[tnl:113492551](https://www.genome.jp/entry/tnl:113492551) [K01432](https://www.genome.jp/entry/K01432) kynurenine formamidase isoform X1

[tnl:113493488](https://www.genome.jp/entry/tnl:113493488) [K07515](https://www.genome.jp/entry/K07515) trifunctional enzyme subunit alpha, mitochondrial

[tnl:113494548](https://www.genome.jp/entry/tnl:113494548) [K00816](https://www.genome.jp/entry/K00816) kynurenine--oxoglutarate transaminase 3 isoform X1

[tnl:113495760](https://www.genome.jp/entry/tnl:113495760) [K03781](https://www.genome.jp/entry/K03781) catalase

[tnl:113498020](https://www.genome.jp/entry/tnl:113498020) [K00626](https://www.genome.jp/entry/K00626) acetyl-CoA acetyltransferase, mitochondrial

[tnl:113500921](https://www.genome.jp/entry/tnl:113500921) [K00128](https://www.genome.jp/entry/K00128) retinal dehydrogenase 1-like

[tnl:113503573](https://www.genome.jp/entry/tnl:113503573) [K00128](https://www.genome.jp/entry/K00128) aldehyde dehydrogenase, mitochondrial

**** [**tnl00051**](https://www.genome.jp/kegg-bin/show_pathway?166859291342340/tnl00051.args) **Fructose and mannose metabolism - Trichoplusia ni (cabbage looper) (**[**8**](javascript:display('tnl00051'))**)**

[tnl:113493924](https://www.genome.jp/entry/tnl:113493924) [K01803](https://www.genome.jp/entry/K01803) triosephosphate isomerase

[tnl:113494165](https://www.genome.jp/entry/tnl:113494165) [K00966](https://www.genome.jp/entry/K00966) mannose-1-phosphate guanyltransferase alpha-A

[tnl:113496310](https://www.genome.jp/entry/tnl:113496310) [K00850](https://www.genome.jp/entry/K00850) ATP-dependent 6-phosphofructokinase isoform X1

[tnl:113496554](https://www.genome.jp/entry/tnl:113496554) [K02377](https://www.genome.jp/entry/K02377) GDP-L-fucose synthase isoform X1

[tnl:113497947](https://www.genome.jp/entry/tnl:113497947) [K00844](https://www.genome.jp/entry/K00844) hexokinase type 2 isoform X1

[tnl:113503730](https://www.genome.jp/entry/tnl:113503730) [K01711](https://www.genome.jp/entry/K01711) GDP-mannose 4,6 dehydratase

[tnl:113505855](https://www.genome.jp/entry/tnl:113505855) [K01809](https://www.genome.jp/entry/K01809) mannose-6-phosphate isomerase

[tnl:113508098](https://www.genome.jp/entry/tnl:113508098) [K17497](https://www.genome.jp/entry/K17497) phosphomannomutase 2

**** [**tnl00590**](https://www.genome.jp/kegg-bin/show_pathway?166859291342340/tnl00590.args) **Arachidonic acid metabolism - Trichoplusia ni (cabbage looper) (**[**8**](javascript:display('tnl00590'))**)**

[tnl:113492686](https://www.genome.jp/entry/tnl:113492686) [K15730](https://www.genome.jp/entry/K15730) uncharacterized protein CG16817-like

[tnl:113493465](https://www.genome.jp/entry/tnl:113493465) [K00079](https://www.genome.jp/entry/K00079) carbonyl reductase [NADPH] 1-like

[tnl:113494468](https://www.genome.jp/entry/tnl:113494468) [K00079](https://www.genome.jp/entry/K00079) carbonyl reductase [NADPH] 3-like

[tnl:113496536](https://www.genome.jp/entry/tnl:113496536) [K15730](https://www.genome.jp/entry/K15730) uncharacterized protein CG16817-like

[tnl:113497263](https://www.genome.jp/entry/tnl:113497263) [K04097](https://www.genome.jp/entry/K04097) glutathione S-transferase 2-like

[tnl:113497936](https://www.genome.jp/entry/tnl:113497936) [K00079](https://www.genome.jp/entry/K00079) carbonyl reductase [NADPH] 1-like

[tnl:113504874](https://www.genome.jp/entry/tnl:113504874) [K01254](https://www.genome.jp/entry/K01254) leukotriene A-4 hydrolase isoform X1

[tnl:113507591](https://www.genome.jp/entry/tnl:113507591) [K15717](https://www.genome.jp/entry/K15717) prostamide/prostaglandin F synthase-like isoform X1

**** [**tnl00280**](https://www.genome.jp/kegg-bin/show_pathway?166859291342340/tnl00280.args) **Valine, leucine and isoleucine degradation - Trichoplusia ni (cabbage looper) (**[**7**](javascript:display('tnl00280'))**)**

[tnl:113493488](https://www.genome.jp/entry/tnl:113493488) [K07515](https://www.genome.jp/entry/K07515) trifunctional enzyme subunit alpha, mitochondrial

[tnl:113494349](https://www.genome.jp/entry/tnl:113494349) [K07509](https://www.genome.jp/entry/K07509) LOW QUALITY PROTEIN: trifunctional enzyme subunit beta, mitochondrial-like

[tnl:113498020](https://www.genome.jp/entry/tnl:113498020) [K00626](https://www.genome.jp/entry/K00626) acetyl-CoA acetyltransferase, mitochondrial

[tnl:113500921](https://www.genome.jp/entry/tnl:113500921) [K00128](https://www.genome.jp/entry/K00128) retinal dehydrogenase 1-like

[tnl:113503573](https://www.genome.jp/entry/tnl:113503573) [K00128](https://www.genome.jp/entry/K00128) aldehyde dehydrogenase, mitochondrial

[tnl:113506203](https://www.genome.jp/entry/tnl:113506203) [K07509](https://www.genome.jp/entry/K07509) trifunctional enzyme subunit beta, mitochondrial-like

[tnl:113507884](https://www.genome.jp/entry/tnl:113507884) [K00249](https://www.genome.jp/entry/K00249) probable medium-chain specific acyl-CoA dehydrogenase, mitochondrial isoform X1

**** [**tnl04130**](https://www.genome.jp/kegg-bin/show_pathway?166859291342340/tnl04130.args) **SNARE interactions in vesicular transport - Trichoplusia ni (cabbage looper) (**[**7**](javascript:display('tnl04130'))**)**

[tnl:113495661](https://www.genome.jp/entry/tnl:113495661) [K08516](https://www.genome.jp/entry/K08516) synaptobrevin homolog YKT6

[tnl:113503064](https://www.genome.jp/entry/tnl:113503064) [K08509](https://www.genome.jp/entry/K08509) synaptosomal-associated protein 29

[tnl:113503677](https://www.genome.jp/entry/tnl:113503677) [K08493](https://www.genome.jp/entry/K08493) vesicle transport through interaction with t-SNAREs homolog 1A

[tnl:113505867](https://www.genome.jp/entry/tnl:113505867) [K08488](https://www.genome.jp/entry/K08488) syntaxin-7

[tnl:113506778](https://www.genome.jp/entry/tnl:113506778) [K08491](https://www.genome.jp/entry/K08491) uncharacterized protein LOC113506778 isoform X1

[tnl:113507923](https://www.genome.jp/entry/tnl:113507923) [K08515](https://www.genome.jp/entry/K08515) vesicle-associated membrane protein 7

[tnl:113508365](https://www.genome.jp/entry/tnl:113508365) [K13504](https://www.genome.jp/entry/K13504) synaptobrevin-1-like

**** [**tnl04136**](https://www.genome.jp/kegg-bin/show_pathway?166859291342340/tnl04136.args) **Autophagy - other - Trichoplusia ni (cabbage looper) (**[**7**](javascript:display('tnl04136'))**)**

[tnl:113492520](https://www.genome.jp/entry/tnl:113492520) [K08339](https://www.genome.jp/entry/K08339) autophagy protein 5

[tnl:113493077](https://www.genome.jp/entry/tnl:113493077) [K08343](https://www.genome.jp/entry/K08343) ubiquitin-like-conjugating enzyme ATG3

[tnl:113495885](https://www.genome.jp/entry/tnl:113495885) [K08341](https://www.genome.jp/entry/K08341) gamma-aminobutyric acid receptor-associated protein

[tnl:113495905](https://www.genome.jp/entry/tnl:113495905) [K17907](https://www.genome.jp/entry/K17907) autophagy-related protein 9A

[tnl:113496598](https://www.genome.jp/entry/tnl:113496598) [K17606](https://www.genome.jp/entry/K17606) immunoglobulin-binding protein 1

[tnl:113499516](https://www.genome.jp/entry/tnl:113499516) [K04382](https://www.genome.jp/entry/K04382) serine/threonine-protein phosphatase 2A catalytic subunit beta isoform

[tnl:113507623](https://www.genome.jp/entry/tnl:113507623) [K07203](https://www.genome.jp/entry/K07203) serine/threonine-protein kinase Tor-like isoform X1

**** [**tnl04745**](https://www.genome.jp/kegg-bin/show_pathway?166859291342340/tnl04745.args) **Phototransduction - fly - Trichoplusia ni (cabbage looper) (**[**6**](javascript:display('tnl04745'))**)**

[tnl:113497125](https://www.genome.jp/entry/tnl:113497125) [K05858](https://www.genome.jp/entry/K05858) 1-phosphatidylinositol 4,5-bisphosphate phosphodiesterase classes I and II

[tnl:113499032](https://www.genome.jp/entry/tnl:113499032) [K00910](https://www.genome.jp/entry/K00910) G protein-coupled receptor kinase 1 isoform X1

[tnl:113500847](https://www.genome.jp/entry/tnl:113500847) [K04634](https://www.genome.jp/entry/K04634) guanine nucleotide-binding protein G(q) subunit alpha isoform X1

[tnl:113502491](https://www.genome.jp/entry/tnl:113502491) [K04515](https://www.genome.jp/entry/K04515) calcium/calmodulin-dependent protein kinase type II alpha chain

[tnl:113503987](https://www.genome.jp/entry/tnl:113503987) [K05858](https://www.genome.jp/entry/K05858) 1-phosphatidylinositol 4,5-bisphosphate phosphodiesterase isoform X1

[tnl:113505800](https://www.genome.jp/entry/tnl:113505800) [K02183](https://www.genome.jp/entry/K02183) calmodulin isoform X1

**** [**tnl00920**](https://www.genome.jp/kegg-bin/show_pathway?166859291342340/tnl00920.args) **Sulfur metabolism - Trichoplusia ni (cabbage looper) (**[**6**](javascript:display('tnl00920'))**)**

[tnl:113496966](https://www.genome.jp/entry/tnl:113496966) [K17285](https://www.genome.jp/entry/K17285) methanethiol oxidase

[tnl:113498047](https://www.genome.jp/entry/tnl:113498047) [K15759](https://www.genome.jp/entry/K15759) putative inositol monophosphatase 3

[tnl:113502400](https://www.genome.jp/entry/tnl:113502400) [K13811](https://www.genome.jp/entry/K13811) bifunctional 3'-phosphoadenosine 5'-phosphosulfate synthase isoform X1

[tnl:113504592](https://www.genome.jp/entry/tnl:113504592) [K17725](https://www.genome.jp/entry/K17725) uncharacterized protein LOC113504592

[tnl:113504708](https://www.genome.jp/entry/tnl:113504708) [K01082](https://www.genome.jp/entry/K01082) 3'(2'),5'-bisphosphate nucleotidase 1-like isoform X1

[tnl:113508032](https://www.genome.jp/entry/tnl:113508032) [K01082](https://www.genome.jp/entry/K01082) 3'(2'),5'-bisphosphate nucleotidase 1-like isoform X1

**** [**tnl00020**](https://www.genome.jp/kegg-bin/show_pathway?166859291342340/tnl00020.args) **Citrate cycle (TCA cycle) - Trichoplusia ni (cabbage looper) (**[**6**](javascript:display('tnl00020'))**)**

[tnl:113492358](https://www.genome.jp/entry/tnl:113492358) [K00658](https://www.genome.jp/entry/K00658) dihydrolipoyllysine-residue succinyltransferase component of 2-oxoglutarate dehydrogenase complex, mitochondrial-like

[tnl:113492710](https://www.genome.jp/entry/tnl:113492710) [K00031](https://www.genome.jp/entry/K00031) isocitrate dehydrogenase [NADP] cytoplasmic-like

[tnl:113495603](https://www.genome.jp/entry/tnl:113495603) [K01679](https://www.genome.jp/entry/K01679) fumarate hydratase, mitochondrial-like isoform X1

[tnl:113497832](https://www.genome.jp/entry/tnl:113497832) [K00025](https://www.genome.jp/entry/K00025) malate dehydrogenase, cytoplasmic

[tnl:113504438](https://www.genome.jp/entry/tnl:113504438) [K00031](https://www.genome.jp/entry/K00031) isocitrate dehydrogenase [NADP] cytoplasmic isoform X1

[tnl:113505697](https://www.genome.jp/entry/tnl:113505697) [K01648](https://www.genome.jp/entry/K01648) ATP-citrate synthase

**** [**tnl00511**](https://www.genome.jp/kegg-bin/show_pathway?166859291342340/tnl00511.args) **Other glycan degradation - Trichoplusia ni (cabbage looper) (**[**6**](javascript:display('tnl00511'))**)**

[tnl:113496200](https://www.genome.jp/entry/tnl:113496200) [K01191](https://www.genome.jp/entry/K01191) alpha-mannosidase 2

[tnl:113497289](https://www.genome.jp/entry/tnl:113497289) [K12373](https://www.genome.jp/entry/K12373) chitooligosaccharidolytic beta-N-acetylglucosaminidase

[tnl:113497787](https://www.genome.jp/entry/tnl:113497787) [K17108](https://www.genome.jp/entry/K17108) non-lysosomal glucosylceramidase

[tnl:113498601](https://www.genome.jp/entry/tnl:113498601) [K12373](https://www.genome.jp/entry/K12373) beta-hexosaminidase subunit beta-like isoform X1

[tnl:113505283](https://www.genome.jp/entry/tnl:113505283) [K20730](https://www.genome.jp/entry/K20730) probable beta-hexosaminidase fdl

[tnl:113508089](https://www.genome.jp/entry/tnl:113508089) [K01227](https://www.genome.jp/entry/K01227) uncharacterized protein LOC113508089

 [**tnl00310**](https://www.genome.jp/kegg-bin/show_pathway?166859291342340/tnl00310.args) **Lysine degradation - Trichoplusia ni (cabbage looper) (**[**6**](javascript:display('tnl00310'))**)**

[tnl:113492358](https://www.genome.jp/entry/tnl:113492358) [K00658](https://www.genome.jp/entry/K00658) dihydrolipoyllysine-residue succinyltransferase component of 2-oxoglutarate dehydrogenase complex, mitochondrial-like

[tnl:113493488](https://www.genome.jp/entry/tnl:113493488) [K07515](https://www.genome.jp/entry/K07515) trifunctional enzyme subunit alpha, mitochondrial

[tnl:113495745](https://www.genome.jp/entry/tnl:113495745) [K13647](https://www.genome.jp/entry/K13647) procollagen-lysine,2-oxoglutarate 5-dioxygenase isoform X1

[tnl:113498020](https://www.genome.jp/entry/tnl:113498020) [K00626](https://www.genome.jp/entry/K00626) acetyl-CoA acetyltransferase, mitochondrial

[tnl:113500921](https://www.genome.jp/entry/tnl:113500921) [K00128](https://www.genome.jp/entry/K00128) retinal dehydrogenase 1-like

[tnl:113503573](https://www.genome.jp/entry/tnl:113503573) [K00128](https://www.genome.jp/entry/K00128) aldehyde dehydrogenase, mitochondrial

**** [**tnl00513**](https://www.genome.jp/kegg-bin/show_pathway?166859291342340/tnl00513.args) **Various types of N-glycan biosynthesis - Trichoplusia ni (cabbage looper) (**[**6**](javascript:display('tnl00513'))**)**

[tnl:113496346](https://www.genome.jp/entry/tnl:113496346) [K12667](https://www.genome.jp/entry/K12667) dolichyl-diphosphooligosaccharide--protein glycosyltransferase subunit 2

[tnl:113497289](https://www.genome.jp/entry/tnl:113497289) [K12373](https://www.genome.jp/entry/K12373) chitooligosaccharidolytic beta-N-acetylglucosaminidase

[tnl:113498601](https://www.genome.jp/entry/tnl:113498601) [K12373](https://www.genome.jp/entry/K12373) beta-hexosaminidase subunit beta-like isoform X1

[tnl:113499077](https://www.genome.jp/entry/tnl:113499077) [K01231](https://www.genome.jp/entry/K01231) alpha-mannosidase 2 isoform X1

[tnl:113504454](https://www.genome.jp/entry/tnl:113504454) [K12670](https://www.genome.jp/entry/K12670) dolichyl-diphosphooligosaccharide--protein glycosyltransferase 48 kDa subunit

[tnl:113505283](https://www.genome.jp/entry/tnl:113505283) [K20730](https://www.genome.jp/entry/K20730) probable beta-hexosaminidase fdl

 [tnl00052](https://www.genome.jp/kegg-bin/show_pathway?166859291342340/tnl00052.args) Galactose metabolism - Trichoplusia ni (cabbage looper) ([6](javascript:display('tnl00052')))

[tnl:113496310](https://www.genome.jp/entry/tnl:113496310) [K00850](https://www.genome.jp/entry/K00850) ATP-dependent 6-phosphofructokinase isoform X1

[tnl:113497839](https://www.genome.jp/entry/tnl:113497839) [K12316](https://www.genome.jp/entry/K12316) lysosomal alpha-glucosidase-like

[tnl:113497947](https://www.genome.jp/entry/tnl:113497947) [K00844](https://www.genome.jp/entry/K00844) hexokinase type 2 isoform X1

[tnl:113501660](https://www.genome.jp/entry/tnl:113501660) [K00963](https://www.genome.jp/entry/K00963) UTP--glucose-1-phosphate uridylyltransferase isoform X1

[tnl:113503659](https://www.genome.jp/entry/tnl:113503659) [K01835](https://www.genome.jp/entry/K01835) phosphoglucomutase

[tnl:113503711](https://www.genome.jp/entry/tnl:113503711) [K01784](https://www.genome.jp/entry/K01784) UDP-glucose 4-epimerase-like

**** [**tnl00670**](https://www.genome.jp/kegg-bin/show_pathway?166859291342340/tnl00670.args) **One carbon pool by folate - Trichoplusia ni (cabbage looper) (**[**5**](javascript:display('tnl00670'))**)**

[tnl:113491873](https://www.genome.jp/entry/tnl:113491873) [K00600](https://www.genome.jp/entry/K00600) serine hydroxymethyltransferase, cytosolic isoform X1

[tnl:113500912](https://www.genome.jp/entry/tnl:113500912) [K00602](https://www.genome.jp/entry/K00602) bifunctional purine biosynthesis protein PURH-like

[tnl:113503426](https://www.genome.jp/entry/tnl:113503426) [K13403](https://www.genome.jp/entry/K13403) bifunctional methylenetetrahydrofolate dehydrogenase/cyclohydrolase, mitochondrial isoform X1

[tnl:113504172](https://www.genome.jp/entry/tnl:113504172) [K00287](https://www.genome.jp/entry/K00287) dihydrofolate reductase isoform X1

[tnl:113508501](https://www.genome.jp/entry/tnl:113508501) [K00560](https://www.genome.jp/entry/K00560) thymidylate synthase

**** [**tnl00740**](https://www.genome.jp/kegg-bin/show_pathway?166859291342340/tnl00740.args) **Riboflavin metabolism - Trichoplusia ni (cabbage looper) (**[**5**](javascript:display('tnl00740'))**)**

[tnl:113492846](https://www.genome.jp/entry/tnl:113492846) [K14410](https://www.genome.jp/entry/K14410) prostatic acid phosphatase-like isoform X1

[tnl:113496175](https://www.genome.jp/entry/tnl:113496175) [K14410](https://www.genome.jp/entry/K14410) prostatic acid phosphatase isoform X1

[tnl:113499353](https://www.genome.jp/entry/tnl:113499353) [K00861](https://www.genome.jp/entry/K00861) riboflavin kinase

[tnl:113501879](https://www.genome.jp/entry/tnl:113501879) [K14394](https://www.genome.jp/entry/K14394) low molecular weight phosphotyrosine protein phosphatase 1-like

[tnl:113504959](https://www.genome.jp/entry/tnl:113504959) [K05901](https://www.genome.jp/entry/K05901) flavin reductase (NADPH)

**** [**tnl00450**](https://www.genome.jp/kegg-bin/show_pathway?166859291342340/tnl00450.args) **Selenocompound metabolism - Trichoplusia ni (cabbage looper) (**[**5**](javascript:display('tnl00450'))**)**

[tnl:113494548](https://www.genome.jp/entry/tnl:113494548) [K00816](https://www.genome.jp/entry/K00816) kynurenine--oxoglutarate transaminase 3 isoform X1

[tnl:113502164](https://www.genome.jp/entry/tnl:113502164) [K01758](https://www.genome.jp/entry/K01758) cystathionine gamma-lyase-like

[tnl:113502400](https://www.genome.jp/entry/tnl:113502400) [K13811](https://www.genome.jp/entry/K13811) bifunctional 3'-phosphoadenosine 5'-phosphosulfate synthase isoform X1

[tnl:113504568](https://www.genome.jp/entry/tnl:113504568) [K22182](https://www.genome.jp/entry/K22182) thioredoxin reductase 1, mitochondrial isoform X1

[tnl:113507470](https://www.genome.jp/entry/tnl:113507470) [K01874](https://www.genome.jp/entry/K01874) methionine--tRNA ligase, cytoplasmic

**** [**tnl00760**](https://www.genome.jp/kegg-bin/show_pathway?166859291342340/tnl00760.args) **Nicotinate and nicotinamide metabolism - Trichoplusia ni (cabbage looper) (**[**5**](javascript:display('tnl00760'))**)**

[tnl:113495149](https://www.genome.jp/entry/tnl:113495149) [K06210](https://www.genome.jp/entry/K06210) nicotinamide/nicotinic acid mononucleotide adenylyltransferase 1

[tnl:113496055](https://www.genome.jp/entry/tnl:113496055) [K03783](https://www.genome.jp/entry/K03783) purine nucleoside phosphorylase-like isoform X1

[tnl:113497953](https://www.genome.jp/entry/tnl:113497953) [K01081](https://www.genome.jp/entry/K01081) cytosolic purine 5'-nucleotidase isoform X1

[tnl:113499192](https://www.genome.jp/entry/tnl:113499192) [K10524](https://www.genome.jp/entry/K10524) nicotinamide riboside kinase 2

[tnl:113502186](https://www.genome.jp/entry/tnl:113502186) [K00763](https://www.genome.jp/entry/K00763) nicotinate phosphoribosyltransferase isoform X1

**** [**tnl03060**](https://www.genome.jp/kegg-bin/show_pathway?166859291342340/tnl03060.args) **Protein export - Trichoplusia ni (cabbage looper) (**[**5**](javascript:display('tnl03060'))**)**

[tnl:113492041](https://www.genome.jp/entry/tnl:113492041) [K03107](https://www.genome.jp/entry/K03107) signal recognition particle subunit SRP68

[tnl:113494618](https://www.genome.jp/entry/tnl:113494618) [K09490](https://www.genome.jp/entry/K09490) endoplasmic reticulum chaperone BiP

[tnl:113498464](https://www.genome.jp/entry/tnl:113498464) [K12272](https://www.genome.jp/entry/K12272) signal recognition particle receptor subunit beta

[tnl:113498935](https://www.genome.jp/entry/tnl:113498935) [K03104](https://www.genome.jp/entry/K03104) signal recognition particle 14 kDa protein

[tnl:113500673](https://www.genome.jp/entry/tnl:113500673) [K03105](https://www.genome.jp/entry/K03105) signal recognition particle 19 kDa protein

**** [**tnl00350**](https://www.genome.jp/kegg-bin/show_pathway?166859291342340/tnl00350.args) **Tyrosine metabolism - Trichoplusia ni (cabbage looper) (**[**5**](javascript:display('tnl00350'))**)**

[tnl:113492488](https://www.genome.jp/entry/tnl:113492488) [K01800](https://www.genome.jp/entry/K01800) probable maleylacetoacetate isomerase 2 isoform X1

[tnl:113492937](https://www.genome.jp/entry/tnl:113492937) [K14454](https://www.genome.jp/entry/K14454) aspartate aminotransferase, cytoplasmic

[tnl:113494362](https://www.genome.jp/entry/tnl:113494362) [K07253](https://www.genome.jp/entry/K07253) macrophage migration inhibitory factor-like isoform X1

[tnl:113497863](https://www.genome.jp/entry/tnl:113497863) [K00121](https://www.genome.jp/entry/K00121) alcohol dehydrogenase class-3

[tnl:113499314](https://www.genome.jp/entry/tnl:113499314) [K01555](https://www.genome.jp/entry/K01555) fumarylacetoacetase

**** [**tnl00510**](https://www.genome.jp/kegg-bin/show_pathway?166859291342340/tnl00510.args) **N-Glycan biosynthesis - Trichoplusia ni (cabbage looper) (**[**5**](javascript:display('tnl00510'))**)**

[tnl:113496346](https://www.genome.jp/entry/tnl:113496346) [K12667](https://www.genome.jp/entry/K12667) dolichyl-diphosphooligosaccharide--protein glycosyltransferase subunit 2

[tnl:113499077](https://www.genome.jp/entry/tnl:113499077) [K01231](https://www.genome.jp/entry/K01231) alpha-mannosidase 2 isoform X1

[tnl:113504454](https://www.genome.jp/entry/tnl:113504454) [K12670](https://www.genome.jp/entry/K12670) dolichyl-diphosphooligosaccharide--protein glycosyltransferase 48 kDa subunit

[tnl:113505102](https://www.genome.jp/entry/tnl:113505102) [K05546](https://www.genome.jp/entry/K05546) neutral alpha-glucosidase AB-like isoform X1

[tnl:113506768](https://www.genome.jp/entry/tnl:113506768) [K05546](https://www.genome.jp/entry/K05546) LOW QUALITY PROTEIN: neutral alpha-glucosidase AB-like

**** [**tnl03020**](https://www.genome.jp/kegg-bin/show_pathway?166859291342340/tnl03020.args) **RNA polymerase - Trichoplusia ni (cabbage looper) (**[**5**](javascript:display('tnl03020'))**)**

[tnl:113491784](https://www.genome.jp/entry/tnl:113491784) [K21987](https://www.genome.jp/entry/K21987) uncharacterized protein LOC113491784

[tnl:113493260](https://www.genome.jp/entry/tnl:113493260) [K03010](https://www.genome.jp/entry/K03010) DNA-directed RNA polymerase II subunit RPB2

[tnl:113497881](https://www.genome.jp/entry/tnl:113497881) [K03007](https://www.genome.jp/entry/K03007) DNA-directed RNA polymerases I, II, and III subunit RPABC5

[tnl:113501782](https://www.genome.jp/entry/tnl:113501782) [K03009](https://www.genome.jp/entry/K03009) DNA-directed RNA polymerases I, II, and III subunit RPABC4

[tnl:113504882](https://www.genome.jp/entry/tnl:113504882) [K03027](https://www.genome.jp/entry/K03027) DNA-directed RNA polymerases I and III subunit RPAC1

**** [**tnl04215**](https://www.genome.jp/kegg-bin/show_pathway?166859291342340/tnl04215.args) **Apoptosis - multiple species - Trichoplusia ni (cabbage looper) (**[**5**](javascript:display('tnl04215'))**)**

[tnl:113492628](https://www.genome.jp/entry/tnl:113492628) [K08738](https://www.genome.jp/entry/K08738) cytochrome c

[tnl:113503670](https://www.genome.jp/entry/tnl:113503670) [K04440](https://www.genome.jp/entry/K04440) stress-activated protein kinase JNK isoform X1

[tnl:113504482](https://www.genome.jp/entry/tnl:113504482) [K20009](https://www.genome.jp/entry/K20009) caspase Dronc

[tnl:113504517](https://www.genome.jp/entry/tnl:113504517) [K04397](https://www.genome.jp/entry/K04397) caspase-1-like

[tnl:113504518](https://www.genome.jp/entry/tnl:113504518) [K04397](https://www.genome.jp/entry/K04397) caspase-1

 [**tnl04392**](https://www.genome.jp/kegg-bin/show_pathway?166859291342340/tnl04392.args) **Hippo signaling pathway - multiple species - Trichoplusia ni (cabbage looper) (**[**5**](javascript:display('tnl04392'))**)**

[tnl:113493261](https://www.genome.jp/entry/tnl:113493261) [K16682](https://www.genome.jp/entry/K16682) LIM domain-containing protein jub

[tnl:113493343](https://www.genome.jp/entry/tnl:113493343) [K06685](https://www.genome.jp/entry/K06685) MOB kinase activator-like 1

[tnl:113500953](https://www.genome.jp/entry/tnl:113500953) [K04412](https://www.genome.jp/entry/K04412) serine/threonine-protein kinase 4 isoform X1

[tnl:113502200](https://www.genome.jp/entry/tnl:113502200) [K16687](https://www.genome.jp/entry/K16687) transcriptional coactivator YAP1-like isoform X1

[tnl:113502799](https://www.genome.jp/entry/tnl:113502799) [K16684](https://www.genome.jp/entry/K16684) moesin/ezrin/radixin homolog 2 isoform X1

**** [**tnl03460**](https://www.genome.jp/kegg-bin/show_pathway?166859291342340/tnl03460.args) **Fanconi anemia pathway - Trichoplusia ni (cabbage looper) (**[**4**](javascript:display('tnl03460'))**)**

[tnl:113498438](https://www.genome.jp/entry/tnl:113498438) [K07466](https://www.genome.jp/entry/K07466) replication protein A 70 kDa DNA-binding subunit

[tnl:113499345](https://www.genome.jp/entry/tnl:113499345) [K15361](https://www.genome.jp/entry/K15361) WD repeat-containing protein 48 homolog

[tnl:113500421](https://www.genome.jp/entry/tnl:113500421) [K11832](https://www.genome.jp/entry/K11832) probable ubiquitin carboxyl-terminal hydrolase creB

[tnl:113503569](https://www.genome.jp/entry/tnl:113503569) [K10901](https://www.genome.jp/entry/K10901) Bloom syndrome protein homolog

 [**tnl03030**](https://www.genome.jp/kegg-bin/show_pathway?166859291342340/tnl03030.args) **DNA replication - Trichoplusia ni (cabbage looper) (**[**4**](javascript:display('tnl03030'))**)**

[tnl:113492828](https://www.genome.jp/entry/tnl:113492828) [K03469](https://www.genome.jp/entry/K03469) ribonuclease H1-like

[tnl:113495246](https://www.genome.jp/entry/tnl:113495246) [K04802](https://www.genome.jp/entry/K04802) proliferating cell nuclear antigen

[tnl:113498438](https://www.genome.jp/entry/tnl:113498438) [K07466](https://www.genome.jp/entry/K07466) replication protein A 70 kDa DNA-binding subunit

[tnl:113505129](https://www.genome.jp/entry/tnl:113505129) [K02326](https://www.genome.jp/entry/K02326) DNA polymerase epsilon subunit 3

 [**tnl00220**](https://www.genome.jp/kegg-bin/show_pathway?166859291342340/tnl00220.args) **Arginine biosynthesis - Trichoplusia ni (cabbage looper) (**[**4**](javascript:display('tnl00220'))**)**

[tnl:113492937](https://www.genome.jp/entry/tnl:113492937) [K14454](https://www.genome.jp/entry/K14454) aspartate aminotransferase, cytoplasmic

[tnl:113496121](https://www.genome.jp/entry/tnl:113496121) [K01915](https://www.genome.jp/entry/K01915) glutamine synthetase 2 cytoplasmic-like isoform X1

[tnl:113496832](https://www.genome.jp/entry/tnl:113496832) [K00261](https://www.genome.jp/entry/K00261) glutamate dehydrogenase, mitochondrial

[tnl:113498369](https://www.genome.jp/entry/tnl:113498369) [K00814](https://www.genome.jp/entry/K00814) alanine aminotransferase 1-like isoform X1

**** [**tnl00053**](https://www.genome.jp/kegg-bin/show_pathway?166859291342340/tnl00053.args) **Ascorbate and aldarate metabolism - Trichoplusia ni (cabbage looper) (**[**4**](javascript:display('tnl00053'))**)**

[tnl:113500261](https://www.genome.jp/entry/tnl:113500261) [K01053](https://www.genome.jp/entry/K01053) regucalcin-like isoform X1

[tnl:113500921](https://www.genome.jp/entry/tnl:113500921) [K00128](https://www.genome.jp/entry/K00128) retinal dehydrogenase 1-like

[tnl:113503573](https://www.genome.jp/entry/tnl:113503573) [K00128](https://www.genome.jp/entry/K00128) aldehyde dehydrogenase, mitochondrial

[tnl:113507917](https://www.genome.jp/entry/tnl:113507917) [K00012](https://www.genome.jp/entry/K00012) UDP-glucose 6-dehydrogenase

 [**tnl01210**](https://www.genome.jp/kegg-bin/show_pathway?166859291342340/tnl01210.args) **2-Oxocarboxylic acid metabolism - Trichoplusia ni (cabbage looper) (**[**4**](javascript:display('tnl01210'))**)**

[tnl:113492710](https://www.genome.jp/entry/tnl:113492710) [K00031](https://www.genome.jp/entry/K00031) isocitrate dehydrogenase [NADP] cytoplasmic-like

[tnl:113492937](https://www.genome.jp/entry/tnl:113492937) [K14454](https://www.genome.jp/entry/K14454) aspartate aminotransferase, cytoplasmic

[tnl:113498369](https://www.genome.jp/entry/tnl:113498369) [K00814](https://www.genome.jp/entry/K00814) alanine aminotransferase 1-like isoform X1

[tnl:113504438](https://www.genome.jp/entry/tnl:113504438) [K00031](https://www.genome.jp/entry/K00031) isocitrate dehydrogenase [NADP] cytoplasmic isoform X1

**** [**tnl03440**](https://www.genome.jp/kegg-bin/show_pathway?166859291342340/tnl03440.args) **Homologous recombination - Trichoplusia ni (cabbage looper) (**[**4**](javascript:display('tnl03440'))**)**

[tnl:113492382](https://www.genome.jp/entry/tnl:113492382) [K10683](https://www.genome.jp/entry/K10683) BRCA1-associated RING domain protein 1-like

[tnl:113498438](https://www.genome.jp/entry/tnl:113498438) [K07466](https://www.genome.jp/entry/K07466) replication protein A 70 kDa DNA-binding subunit

[tnl:113503569](https://www.genome.jp/entry/tnl:113503569) [K10901](https://www.genome.jp/entry/K10901) Bloom syndrome protein homolog

[tnl:113508016](https://www.genome.jp/entry/tnl:113508016) [K11864](https://www.genome.jp/entry/K11864) lys-63-specific deubiquitinase BRCC36-like

**** [**tnl00514**](https://www.genome.jp/kegg-bin/show_pathway?166859291342340/tnl00514.args) **Other types of O-glycan biosynthesis - Trichoplusia ni (cabbage looper) (**[**4**](javascript:display('tnl00514'))**)**

[tnl:113493984](https://www.genome.jp/entry/tnl:113493984) [K00710](https://www.genome.jp/entry/K00710) polypeptide N-acetylgalactosaminyltransferase 35A-like isoform X1

[tnl:113497274](https://www.genome.jp/entry/tnl:113497274) [K00710](https://www.genome.jp/entry/K00710) N-acetylgalactosaminyltransferase 7

[tnl:113498917](https://www.genome.jp/entry/tnl:113498917) [K00710](https://www.genome.jp/entry/K00710) putative polypeptide N-acetylgalactosaminyltransferase 9 isoform X1

[tnl:113507745](https://www.genome.jp/entry/tnl:113507745) [K00710](https://www.genome.jp/entry/K00710) polypeptide N-acetylgalactosaminyltransferase 5

**** [**tnl00512**](https://www.genome.jp/kegg-bin/show_pathway?166859291342340/tnl00512.args) **Mucin type O-glycan biosynthesis - Trichoplusia ni (cabbage looper) (**[**4**](javascript:display('tnl00512'))**)**

[tnl:113493984](https://www.genome.jp/entry/tnl:113493984) [K00710](https://www.genome.jp/entry/K00710) polypeptide N-acetylgalactosaminyltransferase 35A-like isoform X1

[tnl:113497274](https://www.genome.jp/entry/tnl:113497274) [K00710](https://www.genome.jp/entry/K00710) N-acetylgalactosaminyltransferase 7

[tnl:113498917](https://www.genome.jp/entry/tnl:113498917) [K00710](https://www.genome.jp/entry/K00710) putative polypeptide N-acetylgalactosaminyltransferase 9 isoform X1

[tnl:113507745](https://www.genome.jp/entry/tnl:113507745) [K00710](https://www.genome.jp/entry/K00710) polypeptide N-acetylgalactosaminyltransferase 5

**** [**tnl03420**](https://www.genome.jp/kegg-bin/show_pathway?166859291342340/tnl03420.args) **Nucleotide excision repair - Trichoplusia ni (cabbage looper) (**[**4**](javascript:display('tnl03420'))**)**

[tnl:113495246](https://www.genome.jp/entry/tnl:113495246) [K04802](https://www.genome.jp/entry/K04802) proliferating cell nuclear antigen

[tnl:113498438](https://www.genome.jp/entry/tnl:113498438) [K07466](https://www.genome.jp/entry/K07466) replication protein A 70 kDa DNA-binding subunit

[tnl:113505129](https://www.genome.jp/entry/tnl:113505129) [K02326](https://www.genome.jp/entry/K02326) DNA polymerase epsilon subunit 3

[tnl:113508021](https://www.genome.jp/entry/tnl:113508021) [K10839](https://www.genome.jp/entry/K10839) UV excision repair protein RAD23 homolog A

 [**tnl00531**](https://www.genome.jp/kegg-bin/show_pathway?166859291342340/tnl00531.args) **Glycosaminoglycan degradation - Trichoplusia ni (cabbage looper) (**[**4**](javascript:display('tnl00531'))**)**

[tnl:113494320](https://www.genome.jp/entry/tnl:113494320) [K10532](https://www.genome.jp/entry/K10532) heparan-alpha-glucosaminide N-acetyltransferase-like

[tnl:113497289](https://www.genome.jp/entry/tnl:113497289) [K12373](https://www.genome.jp/entry/K12373) chitooligosaccharidolytic beta-N-acetylglucosaminidase

[tnl:113498601](https://www.genome.jp/entry/tnl:113498601) [K12373](https://www.genome.jp/entry/K12373) beta-hexosaminidase subunit beta-like isoform X1

[tnl:113506141](https://www.genome.jp/entry/tnl:113506141) [K07964](https://www.genome.jp/entry/K07964) heparanase-like

**** [**tnl00830**](https://www.genome.jp/kegg-bin/show_pathway?166859291342340/tnl00830.args) **Retinol metabolism - Trichoplusia ni (cabbage looper) (**[**4**](javascript:display('tnl00830'))**)**

[tnl:113492848](https://www.genome.jp/entry/tnl:113492848) [K11153](https://www.genome.jp/entry/K11153) retinol dehydrogenase 13-like

[tnl:113497863](https://www.genome.jp/entry/tnl:113497863) [K00121](https://www.genome.jp/entry/K00121) alcohol dehydrogenase class-3

[tnl:113506824](https://www.genome.jp/entry/tnl:113506824) [K15734](https://www.genome.jp/entry/K15734) short-chain dehydrogenase/reductase family 16C member 6-like

[tnl:113507735](https://www.genome.jp/entry/tnl:113507735) [K15734](https://www.genome.jp/entry/K15734) short-chain dehydrogenase/reductase family 16C member 6-like isoform X1

 [**tnl00860**](https://www.genome.jp/kegg-bin/show_pathway?166859291342340/tnl00860.args) **Porphyrin metabolism - Trichoplusia ni (cabbage looper) (**[**4**](javascript:display('tnl00860'))**)**

[tnl:113494620](https://www.genome.jp/entry/tnl:113494620) [K01698](https://www.genome.jp/entry/K01698) delta-aminolevulinic acid dehydratase

[tnl:113498448](https://www.genome.jp/entry/tnl:113498448) [K14163](https://www.genome.jp/entry/K14163) bifunctional glutamate/proline--tRNA ligase isoform X1

[tnl:113504959](https://www.genome.jp/entry/tnl:113504959) [K05901](https://www.genome.jp/entry/K05901) flavin reductase (NADPH)

[tnl:113508348](https://www.genome.jp/entry/tnl:113508348) [K01599](https://www.genome.jp/entry/K01599) uroporphyrinogen decarboxylase

**** [**tnl00410**](https://www.genome.jp/kegg-bin/show_pathway?166859291342340/tnl00410.args) **beta-Alanine metabolism - Trichoplusia ni (cabbage looper) (**[**4**](javascript:display('tnl00410'))**)**

[tnl:113492664](https://www.genome.jp/entry/tnl:113492664) [K08660](https://www.genome.jp/entry/K08660) cytosolic non-specific dipeptidase

[tnl:113493488](https://www.genome.jp/entry/tnl:113493488) [K07515](https://www.genome.jp/entry/K07515) trifunctional enzyme subunit alpha, mitochondrial

[tnl:113500921](https://www.genome.jp/entry/tnl:113500921) [K00128](https://www.genome.jp/entry/K00128) retinal dehydrogenase 1-like

[tnl:113503573](https://www.genome.jp/entry/tnl:113503573) [K00128](https://www.genome.jp/entry/K00128) aldehyde dehydrogenase, mitochondrial

**** [**tnl00600**](https://www.genome.jp/kegg-bin/show_pathway?166859291342340/tnl00600.args) **Sphingolipid metabolism - Trichoplusia ni (cabbage looper) (**[**4**](javascript:display('tnl00600'))**)**

[tnl:113497126](https://www.genome.jp/entry/tnl:113497126) [K00654](https://www.genome.jp/entry/K00654) serine palmitoyltransferase 2

[tnl:113497289](https://www.genome.jp/entry/tnl:113497289) [K12373](https://www.genome.jp/entry/K12373) chitooligosaccharidolytic beta-N-acetylglucosaminidase

[tnl:113497787](https://www.genome.jp/entry/tnl:113497787) [K17108](https://www.genome.jp/entry/K17108) non-lysosomal glucosylceramidase

[tnl:113498601](https://www.genome.jp/entry/tnl:113498601) [K12373](https://www.genome.jp/entry/K12373) beta-hexosaminidase subunit beta-like isoform X1

**** [**tnl00564**](https://www.genome.jp/kegg-bin/show_pathway?166859291342340/tnl00564.args) **Glycerophospholipid metabolism - Trichoplusia ni (cabbage looper) (**[**4**](javascript:display('tnl00564'))**)**

[tnl:113495930](https://www.genome.jp/entry/tnl:113495930) [K06130](https://www.genome.jp/entry/K06130) acyl-protein thioesterase 1

[tnl:113500918](https://www.genome.jp/entry/tnl:113500918) [K00006](https://www.genome.jp/entry/K00006) glycerol-3-phosphate dehydrogenase [NAD(+)], cytoplasmic isoform X1

[tnl:113501133](https://www.genome.jp/entry/tnl:113501133) [K00967](https://www.genome.jp/entry/K00967) ethanolamine-phosphate cytidylyltransferase isoform X1

[tnl:113507902](https://www.genome.jp/entry/tnl:113507902) [K00968](https://www.genome.jp/entry/K00968) choline-phosphate cytidylyltransferase B-like isoform X1

 [**tnl00900**](https://www.genome.jp/kegg-bin/show_pathway?166859291342340/tnl00900.args) **Terpenoid backbone biosynthesis - Trichoplusia ni (cabbage looper) (**[**4**](javascript:display('tnl00900'))**)**

[tnl:113498020](https://www.genome.jp/entry/tnl:113498020) [K00626](https://www.genome.jp/entry/K00626) acetyl-CoA acetyltransferase, mitochondrial

[tnl:113498340](https://www.genome.jp/entry/tnl:113498340) [K05955](https://www.genome.jp/entry/K05955) protein farnesyltransferase/geranylgeranyltransferase type-1 subunit alpha

[tnl:113499532](https://www.genome.jp/entry/tnl:113499532) [K01597](https://www.genome.jp/entry/K01597) diphosphomevalonate decarboxylase-like

[tnl:113504766](https://www.genome.jp/entry/tnl:113504766) [K05954](https://www.genome.jp/entry/K05954) protein farnesyltransferase subunit beta

**** [**tnl00981**](https://www.genome.jp/kegg-bin/show_pathway?166859291342340/tnl00981.args) **Insect hormone biosynthesis - Trichoplusia ni (cabbage looper) (**[**4**](javascript:display('tnl00981'))**)**

[tnl:113499413](https://www.genome.jp/entry/tnl:113499413) [K10719](https://www.genome.jp/entry/K10719) juvenile hormone epoxide hydrolase-like

[tnl:113500921](https://www.genome.jp/entry/tnl:113500921) [K00128](https://www.genome.jp/entry/K00128) retinal dehydrogenase 1-like

[tnl:113501465](https://www.genome.jp/entry/tnl:113501465) [K10719](https://www.genome.jp/entry/K10719) LOW QUALITY PROTEIN: juvenile hormone epoxide hydrolase-like

[tnl:113503573](https://www.genome.jp/entry/tnl:113503573) [K00128](https://www.genome.jp/entry/K00128) aldehyde dehydrogenase, mitochondrial

**** [**tnl00040**](https://www.genome.jp/kegg-bin/show_pathway?166859291342340/tnl00040.args) **Pentose and glucuronate interconversions - Trichoplusia ni (cabbage looper) (**[**3**](javascript:display('tnl00040'))**)**

[tnl:113492142](https://www.genome.jp/entry/tnl:113492142) [K03331](https://www.genome.jp/entry/K03331) L-xylulose reductase-like

[tnl:113501660](https://www.genome.jp/entry/tnl:113501660) [K00963](https://www.genome.jp/entry/K00963) UTP--glucose-1-phosphate uridylyltransferase isoform X1

[tnl:113507917](https://www.genome.jp/entry/tnl:113507917) [K00012](https://www.genome.jp/entry/K00012) UDP-glucose 6-dehydrogenase

**** [**tnl00561**](https://www.genome.jp/kegg-bin/show_pathway?166859291342340/tnl00561.args) **Glycerolipid metabolism - Trichoplusia ni (cabbage looper) (**[**3**](javascript:display('tnl00561'))**)**

[tnl:113500107](https://www.genome.jp/entry/tnl:113500107) [K00864](https://www.genome.jp/entry/K00864) glycerol kinase

[tnl:113500921](https://www.genome.jp/entry/tnl:113500921) [K00128](https://www.genome.jp/entry/K00128) retinal dehydrogenase 1-like

[tnl:113503573](https://www.genome.jp/entry/tnl:113503573) [K00128](https://www.genome.jp/entry/K00128) aldehyde dehydrogenase, mitochondrial

**** [**tnl00730**](https://www.genome.jp/kegg-bin/show_pathway?166859291342340/tnl00730.args) **Thiamine metabolism - Trichoplusia ni (cabbage looper) (**[**3**](javascript:display('tnl00730'))**)**

[tnl:113496813](https://www.genome.jp/entry/tnl:113496813) [K00939](https://www.genome.jp/entry/K00939) adenylate kinase

[tnl:113501879](https://www.genome.jp/entry/tnl:113501879) [K14394](https://www.genome.jp/entry/K14394) low molecular weight phosphotyrosine protein phosphatase 1-like

[tnl:113503650](https://www.genome.jp/entry/tnl:113503650) [K01077](https://www.genome.jp/entry/K01077) alkaline phosphatase-like

**** [**tnl00061**](https://www.genome.jp/kegg-bin/show_pathway?166859291342340/tnl00061.args) **Fatty acid biosynthesis - Trichoplusia ni (cabbage looper) (**[**3**](javascript:display('tnl00061'))**)**

[tnl:113493061](https://www.genome.jp/entry/tnl:113493061) [K01897](https://www.genome.jp/entry/K01897) long-chain-fatty-acid--CoA ligase 4 isoform X1

[tnl:113498344](https://www.genome.jp/entry/tnl:113498344) [K00665](https://www.genome.jp/entry/K00665) fatty acid synthase isoform X1

[tnl:113498903](https://www.genome.jp/entry/tnl:113498903) [K11262](https://www.genome.jp/entry/K11262) acetyl-CoA carboxylase isoform X1

**** [**tnl00750**](https://www.genome.jp/kegg-bin/show_pathway?166859291342340/tnl00750.args) **Vitamin B6 metabolism - Trichoplusia ni (cabbage looper) (**[**3**](javascript:display('tnl00750'))**)**

[tnl:113498462](https://www.genome.jp/entry/tnl:113498462) [K00275](https://www.genome.jp/entry/K00275) pyridoxine-5'-phosphate oxidase-like

[tnl:113499034](https://www.genome.jp/entry/tnl:113499034) [K00868](https://www.genome.jp/entry/K00868) pyridoxal kinase

[tnl:113500574](https://www.genome.jp/entry/tnl:113500574) [K00831](https://www.genome.jp/entry/K00831) probable phosphoserine aminotransferase

**** [**tnl00340**](https://www.genome.jp/kegg-bin/show_pathway?166859291342340/tnl00340.args) **Histidine metabolism - Trichoplusia ni (cabbage looper) (**[**3**](javascript:display('tnl00340'))**)**

[tnl:113492664](https://www.genome.jp/entry/tnl:113492664) [K08660](https://www.genome.jp/entry/K08660) cytosolic non-specific dipeptidase

[tnl:113500921](https://www.genome.jp/entry/tnl:113500921) [K00128](https://www.genome.jp/entry/K00128) retinal dehydrogenase 1-like

[tnl:113503573](https://www.genome.jp/entry/tnl:113503573) [K00128](https://www.genome.jp/entry/K00128) aldehyde dehydrogenase, mitochondrial

**** [**tnl00640**](https://www.genome.jp/kegg-bin/show_pathway?166859291342340/tnl00640.args) **Propanoate metabolism - Trichoplusia ni (cabbage looper) (**[**3**](javascript:display('tnl00640'))**)**

[tnl:113493488](https://www.genome.jp/entry/tnl:113493488) [K07515](https://www.genome.jp/entry/K07515) trifunctional enzyme subunit alpha, mitochondrial

[tnl:113498903](https://www.genome.jp/entry/tnl:113498903) [K11262](https://www.genome.jp/entry/K11262) acetyl-CoA carboxylase isoform X1

[tnl:113506948](https://www.genome.jp/entry/tnl:113506948) [K00016](https://www.genome.jp/entry/K00016) L-lactate dehydrogenase-like isoform X1

 [**tnl03410**](https://www.genome.jp/kegg-bin/show_pathway?166859291342340/tnl03410.args) **Base excision repair - Trichoplusia ni (cabbage looper) (**[**3**](javascript:display('tnl03410'))**)**

[tnl:113495246](https://www.genome.jp/entry/tnl:113495246) [K04802](https://www.genome.jp/entry/K04802) proliferating cell nuclear antigen

[tnl:113505129](https://www.genome.jp/entry/tnl:113505129) [K02326](https://www.genome.jp/entry/K02326) DNA polymerase epsilon subunit 3

[tnl:113508536](https://www.genome.jp/entry/tnl:113508536) [K10771](https://www.genome.jp/entry/K10771) DNA-(apurinic or apyrimidinic site) lyase

**** [**tnl00062**](https://www.genome.jp/kegg-bin/show_pathway?166859291342340/tnl00062.args) **Fatty acid elongation - Trichoplusia ni (cabbage looper) (**[**3**](javascript:display('tnl00062'))**)**

[tnl:113493488](https://www.genome.jp/entry/tnl:113493488) [K07515](https://www.genome.jp/entry/K07515) trifunctional enzyme subunit alpha, mitochondrial

[tnl:113494349](https://www.genome.jp/entry/tnl:113494349) [K07509](https://www.genome.jp/entry/K07509) LOW QUALITY PROTEIN: trifunctional enzyme subunit beta, mitochondrial-like

[tnl:113506203](https://www.genome.jp/entry/tnl:113506203) [K07509](https://www.genome.jp/entry/K07509) trifunctional enzyme subunit beta, mitochondrial-like

**** [**tnl03430**](https://www.genome.jp/kegg-bin/show_pathway?166859291342340/tnl03430.args) **Mismatch repair - Trichoplusia ni (cabbage looper) (**[**2**](javascript:display('tnl03430'))**)**

[tnl:113495246](https://www.genome.jp/entry/tnl:113495246) [K04802](https://www.genome.jp/entry/K04802) proliferating cell nuclear antigen

[tnl:113498438](https://www.genome.jp/entry/tnl:113498438) [K07466](https://www.genome.jp/entry/K07466) replication protein A 70 kDa DNA-binding subunit

**** [**tnl04080**](https://www.genome.jp/kegg-bin/show_pathway?166859291342340/tnl04080.args) **Neuroactive ligand-receptor interaction - Trichoplusia ni (cabbage looper) (**[**2**](javascript:display('tnl04080'))**)**

[tnl:113497386](https://www.genome.jp/entry/tnl:113497386) [K04615](https://www.genome.jp/entry/K04615) gamma-aminobutyric acid type B receptor subunit 2

[tnl:113506351](https://www.genome.jp/entry/tnl:113506351) [K04237](https://www.genome.jp/entry/K04237) partitioning defective 3 homolog isoform X1

**** [**tnl00130**](https://www.genome.jp/kegg-bin/show_pathway?166859291342340/tnl00130.args) **Ubiquinone and other terpenoid-quinone biosynthesis - Trichoplusia ni (cabbage looper) (**[**2**](javascript:display('tnl00130'))**)**

[tnl:113493425](https://www.genome.jp/entry/tnl:113493425) [K01904](https://www.genome.jp/entry/K01904) luciferin 4-monooxygenase-like

[tnl:113504203](https://www.genome.jp/entry/tnl:113504203) [K01904](https://www.genome.jp/entry/K01904) 4-coumarate--CoA ligase 1-like

**** [**tnl00440**](https://www.genome.jp/kegg-bin/show_pathway?166859291342340/tnl00440.args) **Phosphonate and phosphinate metabolism - Trichoplusia ni (cabbage looper) (**[**2**](javascript:display('tnl00440'))**)**

[tnl:113501133](https://www.genome.jp/entry/tnl:113501133) [K00967](https://www.genome.jp/entry/K00967) ethanolamine-phosphate cytidylyltransferase isoform X1

[tnl:113507902](https://www.genome.jp/entry/tnl:113507902) [K00968](https://www.genome.jp/entry/K00968) choline-phosphate cytidylyltransferase B-like isoform X1

**** [**tnl00770**](https://www.genome.jp/kegg-bin/show_pathway?166859291342340/tnl00770.args) **Pantothenate and CoA biosynthesis - Trichoplusia ni (cabbage looper) (**[**2**](javascript:display('tnl00770'))**)**

[tnl:113500921](https://www.genome.jp/entry/tnl:113500921) [K00128](https://www.genome.jp/entry/K00128) retinal dehydrogenase 1-like

[tnl:113503573](https://www.genome.jp/entry/tnl:113503573) [K00128](https://www.genome.jp/entry/K00128) aldehyde dehydrogenase, mitochondrial

 [**tnl00604**](https://www.genome.jp/kegg-bin/show_pathway?166859291342340/tnl00604.args) **Glycosphingolipid biosynthesis - ganglio series - Trichoplusia ni (cabbage looper) (**[**2**](javascript:display('tnl00604'))**)**

[tnl:113497289](https://www.genome.jp/entry/tnl:113497289) [K12373](https://www.genome.jp/entry/K12373) chitooligosaccharidolytic beta-N-acetylglucosaminidase

[tnl:113498601](https://www.genome.jp/entry/tnl:113498601) [K12373](https://www.genome.jp/entry/K12373) beta-hexosaminidase subunit beta-like isoform X1

**** [**tnl00603**](https://www.genome.jp/kegg-bin/show_pathway?166859291342340/tnl00603.args) **Glycosphingolipid biosynthesis - globo and isoglobo series - Trichoplusia ni (cabbage looper) (**[**2**](javascript:display('tnl00603'))**)**

[tnl:113497289](https://www.genome.jp/entry/tnl:113497289) [K12373](https://www.genome.jp/entry/K12373) chitooligosaccharidolytic beta-N-acetylglucosaminidase

[tnl:113498601](https://www.genome.jp/entry/tnl:113498601) [K12373](https://www.genome.jp/entry/K12373) beta-hexosaminidase subunit beta-like isoform X1

**** [**tnl00565**](https://www.genome.jp/kegg-bin/show_pathway?166859291342340/tnl00565.args) **Ether lipid metabolism - Trichoplusia ni (cabbage looper) (**[**2**](javascript:display('tnl00565'))**)**

[tnl:113498502](https://www.genome.jp/entry/tnl:113498502) [K16794](https://www.genome.jp/entry/K16794) LOW QUALITY PROTEIN: lissencephaly-1 homolog

[tnl:113498503](https://www.genome.jp/entry/tnl:113498503) [K16794](https://www.genome.jp/entry/K16794) LOW QUALITY PROTEIN: lissencephaly-1 homolog

**** [**tnl00910**](https://www.genome.jp/kegg-bin/show_pathway?166859291342340/tnl00910.args) **Nitrogen metabolism - Trichoplusia ni (cabbage looper) (**[**2**](javascript:display('tnl00910'))**)**

[tnl:113496121](https://www.genome.jp/entry/tnl:113496121) [K01915](https://www.genome.jp/entry/K01915) glutamine synthetase 2 cytoplasmic-like isoform X1

[tnl:113496832](https://www.genome.jp/entry/tnl:113496832) [K00261](https://www.genome.jp/entry/K00261) glutamate dehydrogenase, mitochondrial

 [**tnl00360**](https://www.genome.jp/kegg-bin/show_pathway?166859291342340/tnl00360.args) **Phenylalanine metabolism - Trichoplusia ni (cabbage looper) (**[**2**](javascript:display('tnl00360'))**)**

[tnl:113492937](https://www.genome.jp/entry/tnl:113492937) [K14454](https://www.genome.jp/entry/K14454) aspartate aminotransferase, cytoplasmic

[tnl:113494362](https://www.genome.jp/entry/tnl:113494362) [K07253](https://www.genome.jp/entry/K07253) macrophage migration inhibitory factor-like isoform X1

 [**tnl00650**](https://www.genome.jp/kegg-bin/show_pathway?166859291342340/tnl00650.args) **Butanoate metabolism - Trichoplusia ni (cabbage looper) (**[**2**](javascript:display('tnl00650'))**)**

[tnl:113493488](https://www.genome.jp/entry/tnl:113493488) [K07515](https://www.genome.jp/entry/K07515) trifunctional enzyme subunit alpha, mitochondrial

[tnl:113498020](https://www.genome.jp/entry/tnl:113498020) [K00626](https://www.genome.jp/entry/K00626) acetyl-CoA acetyltransferase, mitochondrial

**** [**tnl00400**](https://www.genome.jp/kegg-bin/show_pathway?166859291342340/tnl00400.args) **Phenylalanine, tyrosine and tryptophan biosynthesis - Trichoplusia ni (cabbage looper) (**[**1**](javascript:display('tnl00400'))**)**

[tnl:113492937](https://www.genome.jp/entry/tnl:113492937) [K14454](https://www.genome.jp/entry/K14454) aspartate aminotransferase, cytoplasmic

**** [**tnl04122**](https://www.genome.jp/kegg-bin/show_pathway?166859291342340/tnl04122.args) **Sulfur relay system - Trichoplusia ni (cabbage looper) (**[**1**](javascript:display('tnl04122'))**)**

[tnl:113504523](https://www.genome.jp/entry/tnl:113504523) [K14168](https://www.genome.jp/entry/K14168) cytoplasmic tRNA 2-thiolation protein 1

 [**tnl01040**](https://www.genome.jp/kegg-bin/show_pathway?166859291342340/tnl01040.args) **Biosynthesis of unsaturated fatty acids - Trichoplusia ni (cabbage looper) (**[**1**](javascript:display('tnl01040'))**)**

[tnl:113497645](https://www.genome.jp/entry/tnl:113497645) [K00507](https://www.genome.jp/entry/K00507) acyl-CoA Delta(11) desaturase-like

 [**tnl00232**](https://www.genome.jp/kegg-bin/show_pathway?166859291342340/tnl00232.args) **Caffeine metabolism - Trichoplusia ni (cabbage looper) (**[**1**](javascript:display('tnl00232'))**)**

[tnl:113497732](https://www.genome.jp/entry/tnl:113497732) [K00106](https://www.genome.jp/entry/K00106) xanthine dehydrogenase

**** [**tnl00515**](https://www.genome.jp/kegg-bin/show_pathway?166859291342340/tnl00515.args) **Mannose type O-glycan biosynthesis - Trichoplusia ni (cabbage looper) (**[**1**](javascript:display('tnl00515'))**)**

[tnl:113492555](https://www.genome.jp/entry/tnl:113492555) [K21032](https://www.genome.jp/entry/K21032) beta-1,4-glucuronyltransferase 1
